# Intestinally-targeted TGR5 agonists equipped with quaternary ammonium have an improved hypoglycemic effect and reduced gallbladder filling effect

**DOI:** 10.1038/srep28676

**Published:** 2016-06-24

**Authors:** Hua Cao, Zhi-Xiang Chen, Kai Wang, Meng-Meng Ning, Qing-An Zou, Ying Feng, Yang-Liang Ye, Ying Leng, Jian-Hua Shen

**Affiliations:** 1 State Key Laboratory of Drug Research, Shanghai Institute of Materia Medica (SIMM), Chinese Academy of Sciences, Shanghai 201203, China; 2 College of pharmacy, Nanchang University, Nanchang 330006, China

## Abstract

TGR5 activation of enteroendocrine cells increases glucagon-like peptide 1 (GLP-1) release, which maintains glycemic homeostasis. However, TGR5 activation in the gallbladder and heart is associated with severe side effects. Therefore, intestinally-targeted TGR5 agonists were suggested as potential hypoglycemic agents with minimal side effects. However, until now no such compounds with robust glucose-lowering effects were reported, especially in diabetic animal models. Herein, we identify a TGR5 agonist, **26a**, which was proven to be intestinally-targeted through pharmacokinetic studies. **26a** was used as a tool drug to verify the intestinally-targeted strategy. **26a** displayed a robust and long-lasting hypoglycemic effect in *ob*/*ob* mice (once a day dosing (QD) and 18-day treatment) owing to sustained stimulation of GLP-1 secretion, which suggested that robust hypoglycemic effect could be achieved with activation of TGR5 in intestine alone. However, the gallbladder filling effect of **26a** was rather complicated. Although the gallbladder filling effect of **26a** was decreased in mice after once a day dosing, this side effect was still not eliminated. To solve the problem above, several research strategies were raised for further optimization.

TGR5 (Takeda G-protein-coupled receptor 5), also known as GPBAR1, M-BAR, or GPCR19, was identified first as a G protein-coupled receptor responsive to bile acids (BAs) in 2002^1^,^2^. It shows: high expression in the gallbladder; moderate expression in the intestine, spleen and placenta; and low expression in the lung, brown adipose tissue (BAT), skeletal muscle, and brain^3^,^4^,^5^. TGR5 activation in enteroendocrine cells^6^ increases the release of GLP-1 which maintains homeostasis of blood glucose by promoting glucose-induced insulin secretion, suppressing glucagon release, delaying gastric emptying, promoting satiety, and increasing glucose disposal in the peripheral tissues^7^,^8^. In brown adipose tissue and skeletal muscle TGR5 mediates energy expenditure through a BA–TGR5–cAMP–D2 signaling pathway^9^. Therefore, TGR5 activation provides a promising strategy for treatment of type 2 diabetes mellitus and associated metabolic disorders^10^,^11^,^12^. Thus TGR5 has drawn considerable attention from both academia and industry^13^,^14^,^15^,^16^,^17^,^18^.

However, TGR5 activation in other tissues can cause some side effects, of which those in the gallbladder and heart are the main concerns. Assays in mice have revealed that TGR5 activation in the epithelium of the gallbladder by administration of either bile acids derivatives (e. g. INT-777, **1** developed by Intercept Pharmaceuticals, Fig. 1) or synthetic small molecule TGR5 agonist (e. g. **2** developed by our team, Fig. 1) causes smooth-muscle relaxation, prevents bile secretion, and greatly increases gallbladder volume^14^,^19^,^20^. Several absorbed TGR5 agonists have been shown to change heart rate and blood pressure in dogs^15^,^21^,^22^. Therefore, it was suggested that localized activation of TGR5 within the intestinal tract while avoiding systemic exposure (i. e. intestinally-targeted) could be a promising anti-diabetes mellitus strategy with minimal side effects^23^,^24^. While no intestinally-targeted TGR5 agonists with robust activity were reported, especially in a diabetic model, there was still doubt about the validity of this strategy. The first concern was whether robust hypoglycemic efficacy could be achieved by TGR5 activation in the intestine alone without additional effects in the brown adipose tissue or skeletal muscle. The second concern was whether the possible side effects in gallbladder and heart could be eliminated by low systemic drug concentration. Our research team once found a PEG_8_ compound (**3**, Fig. 1) with low systemic exposure, and thus its gallbladder filling effect was reduced. It displayed a moderate hypoglycemic efficacy in normal mice (ICR (Institute of Cancer Research) mice)^25^; however no significant effect in diabetic model *ob*/*ob* mice was observed.

It was apparent that the side effect in heart could be minimized successfully as the drug concentration was decreased in plasma decreases. Therefore, although the gallbladder filling effect is more challenging, it becomes the main focus of our study and key parameter in drug discovery of TGR5 agonist.

Quaternary ammonium is present widely in bile acid sequestrants (BASs) such as cholestyramine (**4**, Fig. 2), colesevelam and colestilan^26^. BASs can bind to BAs in the intestine and act as cholesterol-lowering polymer drugs^27^. BASs are barely absorbed owing to their high molecular weight and positive charge^28^. Recent studies have revealed that BASs can improve glycemic control through induction of energy expenditure, enhance glucose utilization and indirect activation of TGR5^29^,^30^. Quaternary ammonium plays an important part in binding to BAs (so as to improve glycemic control) and the non-absorbed profile. Besides, quaternary ammonium was considered by Exelixis and Searle (now Pfizer) in the development of low absorbed (intestinally targeted) drugs^31^,^32^,^33^. Up to now, only dimer TGR5 agonist **3** with large molecular weight was reported to be low-absorbed, and no other approaches of reducing gallbladder filling effect were reported. We first introduced the quaternary ammonium of BASs to TGR5 agonist. We hypothesized that this approach would increase their hypoglycemic efficacy and greatly decrease systemic exposure to minimize the risk of gallbladder toxicity.

Herein we report the validation of the intestinally-targeted strategy with our newly found low-absorbed TGR5 agonist **26a**.

## Results

**Figure 2:**
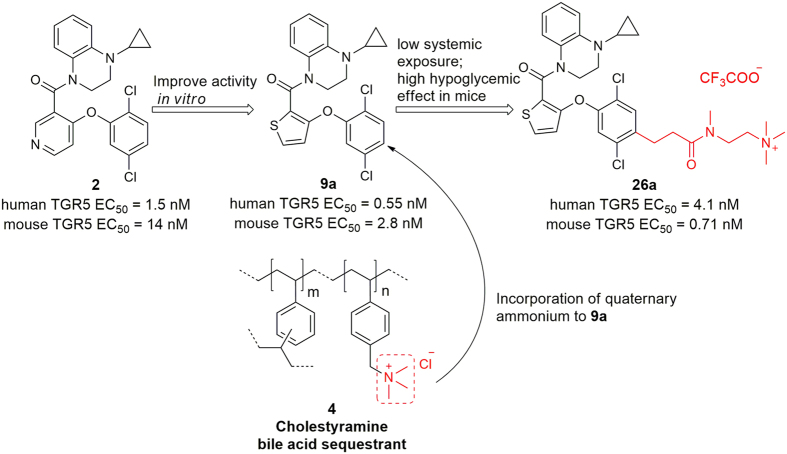
Design strategy of intestinal-targeted TGR5 agonists.

Medicinal chemistry work (including design, structure-activity relationships and biological assays *in vitro*) is available in Supplementary Information. The design strategy is illustrated in Fig. 2. By replacing the pyridine ring of **2** (an absorbed TGR5 agonist, human TGR5 EC_50_ (concentration for 50% of maximal effect) = 1.5 nM, mouse TGR5 EC_50_ = 14 nM) with a thiophene ring, we obtained **9a** with best activity *in vitro* (human TGR5 EC_50_ = 0.55 nM, mouse TGR5 EC_50_ = 2.8 nM). To our delight, **9a** (Papp = 0.55 × 10^−6 ^cm/s, Table 1) showed decreased Caco-2 cell permeability compared with **2** (Papp = 6.75 × 10^−6 ^cm/s). Quaternary ammonium was incorporated to **9a** to yield a series of TGR5 agonists, among which **26a** (human TGR5 EC_50_ = 4.1 nM, mouse TGR5 EC_50_ = 0.71 nM) displayed the best activity *in vitro* and extremely low Caco-2 cell permeability (Papp = 0.06 × 10^−6 ^cm/s). **26a** could be classified as a low-permeability agent.

**26a** met our primary design strategy: low permeability in cell membranes, and highly potent TGR5 activity. What’s more, **26a** exhibited low cLogP = 2.30 (*vs*. cLogP = 5.5 for **2**, calculated by ChemBioDraw Ultra 12.0) and high solubility in water (1.8 mg/ml *vs*.441 ng/ml for **2**, determined by LC-MS/MS). In addition, activation of the farnesyl X receptor (FXR) was not observed up to 100 μM of **26a**, thus suggested that **26a** was a highly selective TGR5 agonist. To investigate whether **26a** with a low-permeability profile could display a robust hypoglycemic effect and a reduced gallbladder filling effect, once a day oral dosing (QD) and long-term effect of **26a** in animal models were determined. A pharmacokinetic profile *in vivo* was also determined to reveal the absorption and distribution of **26a**.

### Evaluation of 26a in ICR Mice

**Figure 3:**
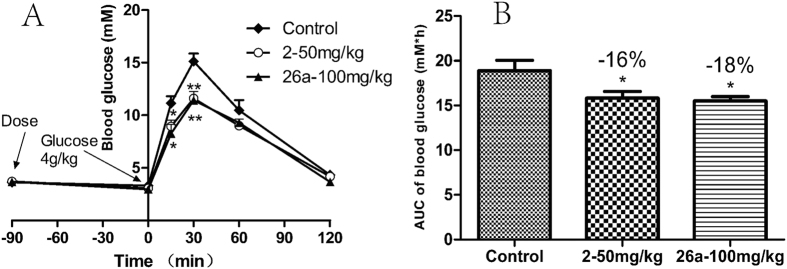
OGTT of 26a (100 mg/kg) and 2 (50 mg/kg) in ICR mice. (**A**) Blood glucose concentration after administration of **26a** and **2**; (**B**) blood glucose AUC_0–120 min_ after administration of **26a** and **2**. Compound **26a**, **2**, and 0.25% CMC (carboxymethyl cellulose sodium, control) were administered (p.o.) to ICR mice (n = 7–8) 1.5 h before oral glucose loading (4 g/kg). Blood glucose levels were measured berefore and after glucose loading. *p < 0.05, **p < 0.01 *vs*. control. Error bar indicateds SEM (standard error of the mean).

The activity of **26a** was investigated using an oral glucose tolerance test (OGTT) in ICR (Institute of Cancer Research) mice. Oral administration of **26a** and **2** resulted in a similar and significant decrease in blood glucose (Fig. 3A,B); the area under the glucose levels *vs*. time curve (AUC) was decreased by 18% and 16% for **26a** and **2**, respectively, compared with that of the control group.

**Figure 4:**
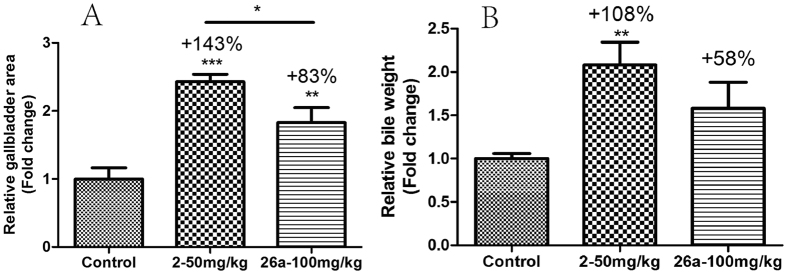
(**A**) Relative gallbladder area and (**B**) relative bile weight after once a day oral dose. After the OGTT experiment, mice were refed for 3 h, the gallbladder was removed and the area measured using a vernier caliper. The relative gallbladder area was calculated from the length multiplied by the width of the gallbladder. Bile weight was measured using analytical balances. *p < 0.05; **p < 0.01; ***p < 0.001. Error bar indicates SEM.

Next we evaluated the effect of **26a** in gallbladder filling in ICR mice (Fig. 4A,B). After once a day oral dose of **2** (50 mg/kg) to ICR mice, gallbladder area and bile weight increased 143% and 108%, respectively, compared with the control group. Encouragingly, the gallbladder filling effect was decreased in the group treated with **26a** (100 mg/kg), as gallbladder area increased 83% compared with the control group, while bile weight increased approximately 58% compared with the control group, which was not a significant difference.

### Evaluation of 26a in *ob*/*ob* Mice

**Figure 5:**
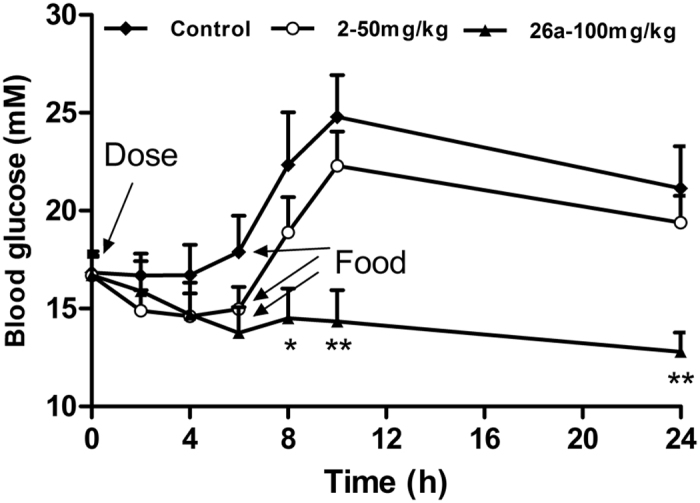
Effects of 26a on blood glucose levels in *ob*/*ob* mice. Compounds **2** (50 mg/kg), **26a** (100 mg/kg) and 0.25% CMC (control) were administered (p.o.) to 2 h-fasted *ob*/*ob* mice (male, n = 8), and blood glucose measured before dosing or 2, 4, 6, 8, 10, and 24 h after dosing. Mice were refed at 6 h after dosing. *p < 0.05, **p < 0.01 *vs*. control. Error bar indicates SEM.

To further assess efficacy, the hypoglycemic effect of **26a** was tested at once a day oral dose (QD) to *ob*/*ob* mice (a genetic type 2 diabetes model with impairment of leptin production). **26a** (100 mg/kg, Fig. 5) displayed a more robust glucose-lowering effect compared with **2** (50 mg/kg), and this effect was maintained until at least 24 h post-dosing.

**Figure 6:**
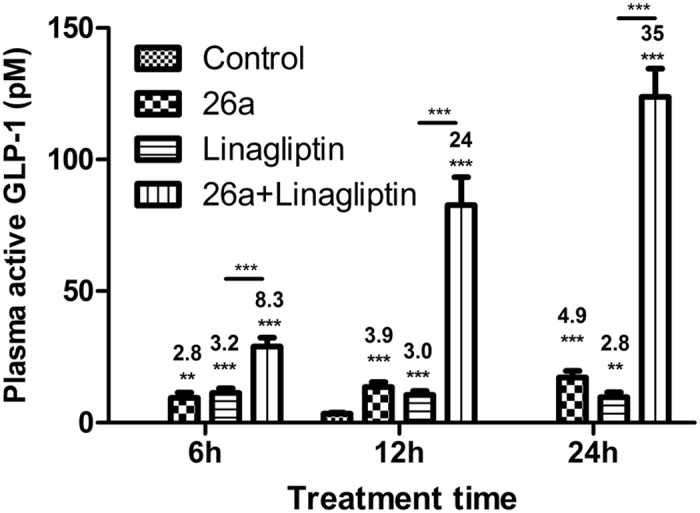
Study of GLP-1 secretion by 26a in *ob*/*ob* mice. Four groups of *ob*/*ob* mice (female, n = 8–9 for each time point per group) were administered 0.25% CMC (control), **26a** (100 mg/kg), linagliptin (3 mg/kg) and **26a** (100 mg/kg) plus linagliptin (3 mg/kg). Blood samples were collected after 6, 12, and 24 h. All the animals were fasted for 6 hours before collecting blood samples at the indicated time points. At other times, animals were free accessed to water and food. All time point shared the same control group collected after 12 h. **p < 0.01; ***p < 0.001 *vs*. control. Error bar indicates SEM. The number above the error bar displays the fold-change over that in the control group.

A GLP-1 secretion assay of **26a** in *ob*/*ob* mice was consistent with the robust and long-lasting hypoglycemic effect. Persistent stimulation of GLP-1 was found up to the 24 h of our experiment (Fig. 6): GLP-1 stimulation using **26a** was 2.8-, 3.9-, and 4.9- fold at 6, 12, and 24 h, respectively, over that observed in the control group. These data confirmed that the hypoglycemic effect of **26a** was reliant mainly on the effect of GLP-1 stimulation from enteroendocrine cells. In addition, the GLP-1 stimulation effect of **26a** was enhanced considerably by linagliptin (a dipeptidyl peptidase (DPP)-4 inhibitor that prevents GLP-1 degradation). Total effect of GLP-1 stimulation was 8.3-, 24-, and 35- fold at 6, 12, and 24 h, respectively, over that in the control group. These results highlighted the considerable therapeutic potential of combining TGR5 agonist **26a** with a DPP-4 inhibitor in treatment of type 2 diabetes.

**Figure 7:**
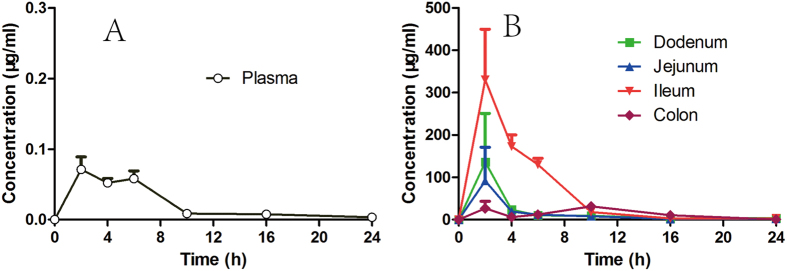
Concentration of 26a in plasma (**A**) and the intestinal tissue (duodenum, jejunum, ileum, colon, **B**) of *ob*/*ob* mice. Once a day oral dose of **26a** (100 mg/kg) was administered to 12 h-fasted *ob*/*ob* mice (male, n = 3). The blood and intestinal tissue samples were collected before dosing or 2, 4, 6, 10, 16, and 24 h after dosing. Density of intestinal tissue was taken as 1 g/ml. Error bar indicates SEM.

The robust hypoglycemic effect of **26a** in mice was consistent with our design purpose. Nevertheless, whether improvement in activity was owing to the BAs binding effect of quaternary ammonium was not known. In a subsequent 18-day treatment of **26a** in *ob*/*ob* mice (Part 8 of the Supplementary Information), a significant change in levels of total cholesterol was not observed, suggesting that **26a** could not chelate BAs. A pharmacokinetic (PK) study of **26a** was carried out to have a better understanding of its high efficacy. After once a day oral dose (QD) of **26a** (100 mg/kg) to *ob*/*ob* mice, plasma and intestinal tissue were collected and analyzed. **26a** exhibited relatively low systemic exposure in *ob*/*ob* mice plasma, with C_max_ (peak drug concentration) of 0.071 μg/mL at 2 h (Fig. 7A). Additionally, no accumulation was found as the drug concentration fell below 0.01 μg/mL after 10 h. The low-absorbed profile of **26a** could explain the reduced gallbladder filling effect, which was consistent with our design strategy. The drug level of **26a** in intestinal tissue (Fig. 7B) was relatively high with a C_max_ (at 2 h) of 136, 92, 331, and 27 μg/ml for the tissue of duodenum, jejunum, ileum, colon, respectively. The level of **26a** in intestinal tissue was greater than 0.8 μg/ml even at 24 h. The high concentration of **26a** in intestinal tissue could have accounted for the robust and long-lasting hypoglycemic effect and persistent GLP-1 stimulation in *ob/ob* mice. The intestinally-targeted profile of **26a** was proven by the high concentration ratio between intestinal tissue and plasma. As mentioned above, TGR5 has moderate expression in the intestinal tract and low expression in BAT and skeletal muscle; therefore, we could assume that the robust hypoglycemic effect was owing mainly to TGR5 activation in enteroendocrine cells. That is, local activation of TGR5 in the intestinal tract can elicit a long-lasting effect on glucose levels.

**Figure 8:**
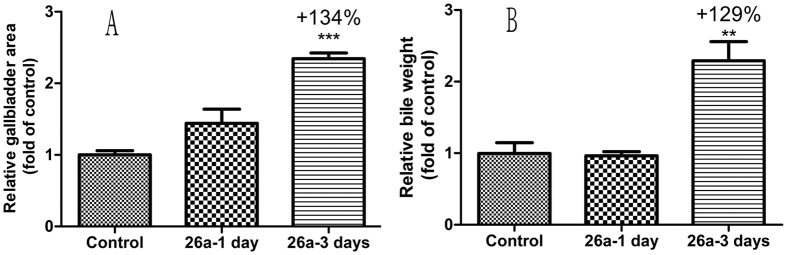
(**A**) Relative gallbladder area and (**B**) relative bile weight after once a day oral dose or 3-day treatment. 26a (100 mg/kg) or 0.25% CMC (control) were administered (p.o.) to overnight-fasted *ob*/*ob* mice (male, n = 3 for control group, n = 5–6 for group of **26a**) for once or 3 days. 1 h after the final dose, mice were re-fed for 3 h and then the gallbladder was removed and the area measured using a vernier caliper. The relative gallbladder area was calculated from the length multiplied by the width of the gallbladder. Bile weight was measured using analytical balances. **p < 0.01; ***p < 0.001. Error bar indicates SEM.

Finally, the gallbladder filling effect of **26a** was measured in *ob*/*ob* mice. Once a day oral dose of **26a** (100 mg/kg, Fig. 8A,B) did not increase gallbladder area or bile weight significantly. However, after the 3-day administration, gallbladder area and bile weight were increased significantly by 134% and 129%, respectively. These data suggested that treatment in *ob*/*ob* mice for 3 days is a more sensitive and suitable model to judge the gallbladder filling effect.

The gallbladder filling effect of *ob/ob* mice after 3-day treatment was not in accordance with the low systemic exposure in the pharmacokinetic study. Hence, further experimentation to ascertain the distribution of **26a** in the gallbladder was carried out. After the gallbladder filling assay, samples of plasma, bile, and gallbladder tissue were collected and evaluated for drug concentration. The concentration of **26a** in plasma was relatively low (18 ± 3 ng/mL), but was higher in the gallbladder and bile (112 ± 30 and 7805 ± 1851 ng/ml, respectively). High exposure in bile suggested that **26a** was excreted from serum to bile. We hypothesized that active transport had an important role in **26a** accumulation in bile. However, the search for corresponding transporters involved in hepatic uptake and biliary excretion was not successful (data not shown). Another possible factor contributing to high exposure of **26a** in bile was that the bile was highly concentrated in the gallbladder. Further studies were underway to have a better understanding of this phenomenon.

### Long-term Study in *ob*/*ob* mice

**Figure 9:**
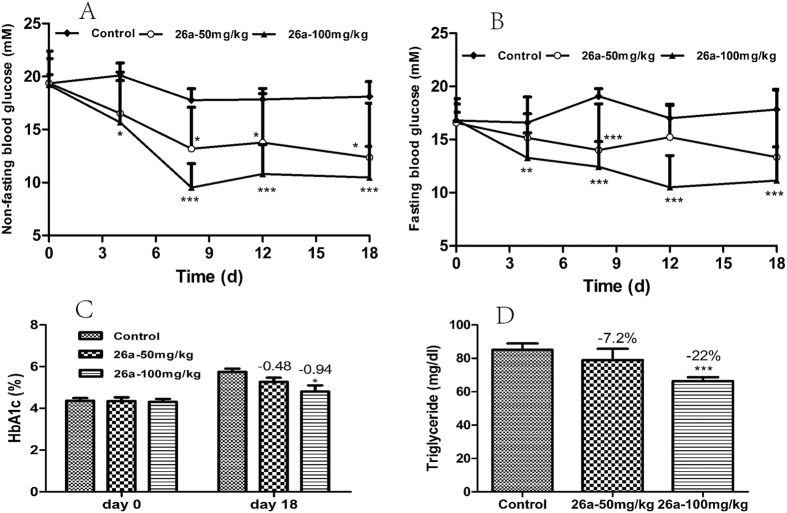
Long-term study of 26a in *ob/ob* mice. Compound **26a** (50 mg/kg or 100 mg/kg) and 0.25% CMC (control) were administered (p.o.) to *ob*/*ob* mice (male, n = 10) for 18 days. (**A**) Non-fasting blood glucose and (**B**) fasting blood glucose in a long-term study of **26a** in *ob*/*ob* mice. (**C**) HbA_1c_ level at day 0 (before dosing) and day 18 (after the final dose) in *ob*/*ob* mice. (**D**) Triglyceride level after the 18-day treatment. *p < 0.05, **p < 0.01, ***p < 0.001 *vs*. control. Error bar indicates SEM.

Because of its robust hypoglycemic effect and relatively low gallbladder filling effect, **26a** was evaluated further in a long-term study in *ob*/*ob* mice for 18 days. Persistent glucose-lowering effect was observed upon 18-day administration of **26a**. Non-fasting and fasting blood glucose were decreased dramatically upon 18-day treatment in *ob*/*ob* mice (Fig. 9A,B), and the glucose-lowering effect was dose-dependent. Hemoglobin A_1c_ (HbA_1c_) refers to glycated or glycosylated hemoglobin and can reflect the long-term blood glucose level. **26a** (100 mg/kg) could decrease the HbA_1c_ levels significantly by 0.94% on day 18 (Fig. 9C), whereas **26a** (50 mg/kg) could decrease the HbA_1c_ levels by 0.48%, but the difference was not significant. The triglyceride level (Fig. 9D) was decreased significantly by 22% after 18-day treatment of **26a** (100 mg/kg). These data suggested the potential therapeutic effect of **26a** in type 2 diabetes and metabolic diseases.

No significant changes in levels of alanine aminotransferase, aspartate aminotransferase, alkaline phosphatase, or total bilirubin were observed (data not shown). Besides, body weight of mice was not affected in the 18-day treatment, suggesting no other apparent toxicity of **26a**.

## Conclusion

We designed and synthesized a series of intestinally-targeted TGR5 agonists, among which **26a** displayed the best *in vitro* activity and extremely low Caco-2 cell permeability, thus it was chosen for validation of the intestinally-targeted strategy. The PK study confirmed its low absorption into systemic circulation and high concentration in the intestinal-tissue, which was in accordance with our design strategy. In the meantime, **26a** (100 mg/kg) exhibited robust and long-lasting hypoglycemic effect in ICR and *ob*/*ob* mice. Furthermore, **26a** exhibited a consistent hypoglycemic effect throughout the 18-day treatment of *ob*/*ob* mice. These data confirmed that a robust hypoglycemic effect could be achieved by intestinal activation of TGR5 independent of systemic exposure. Pleasingly, a decreased gallbladder filling effect was observed after once a day oral dose of **26a** (100 mg/kg) in ICR mice compared with that of absorbed TGR5 agonist **2**. However, after 3-day treatment with **26a** (100 mg/kg) in *ob/ob* mice, gallbladder area and bile weight were increased significantly by 134% and 129% compared with that of the control group, respectively. Further pharmacokinetic study revealed that the drug concentration ratio between bile and plasma of **26a** was relatively high, which suggested that low level of **26a** in plasma was secreted to gallbladder. However, the search for corresponding transporters involved in hepatic uptake and biliary secretion yield no good results. The high correlation between drug concentration in bile rather than plasma with gallbladder filling effect suggests that gallbladder-nonabsorbed instead of systemic-nonabsorbed profile is the key to later optimization, and our further studies on the reduction of the secretion from plasma to gallbladder of intestinally-targeted agonists are underway. Furthermore, the cooperative effect in stimulating GLP-1 secretion of **26a** with a DPP-4 inhibitor suggested that dose reduction of TGR5 agonist may be permitted when combined with DPP-4 inhibitors, thus the side effects might be minimized.

Taken together, our study revealed that TGR5 agonism in intestine alone could bring robust hypoglycemic activity. Although the gallbladder filling effect of **26a** was not eliminated, it was reduced compared with that of systemic absorbed TGR5 agonist. This strategy could provide a foundation for further studies on non-absorbed TGR5 agonists or other intestinally-targeted agents.

## Methods

Medicinal chemistry work and experimental procedure are available in Supplementary Information. Animal experiments were carried out according to the Guidelines for the Care and Use of Laboratory Animals and were approved by the Animal Care and Use Committee, Shanghai Institute of Materia Medica, Chinese Academy of Sciences.

## Additional Information

**How to cite this article**: Cao, H. *et al*. Intestinally-targeted TGR5 agonists equipped with quaternary ammonium have an improved hypoglycemic effect and reduced gallbladder filling effect. *Sci. Rep*. **6**, 28676; doi: 10.1038/srep28676 (2016).

## Supplementary Material

Supplementary Information

## Figures and Tables

**Figure 1:**
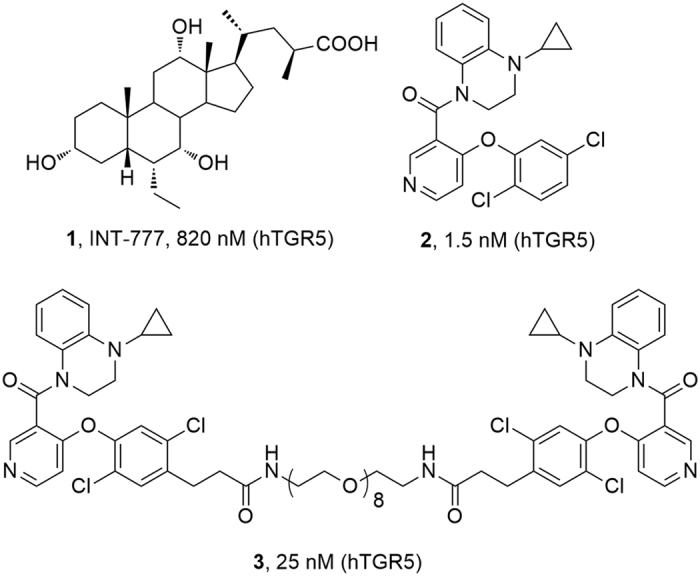
Structures of several TGR5 agonists and their EC_50_ (concentration for 50% of maximal effect) values on human TGR5 (hTGR5).

**Table 1:** Apparent Permeability (Papp) in Caco-2 Cells of 2, 9a, 26a.

Compd	A to B	B to A	Efflux ratio
	Papp (10^−6 ^cm/s)	Papp (10^−6 ^cm/s)	
**2**	6.75	5.58	0.8
**9a**	0.55	0.43	0.8
**26a**	0.06	0.8	14.1

‘A to B’ indicates the experiment from apical to basolateral side, ‘B to A’ indicates the experiment from basolateral to apical side.
