# Supplementary material for: Intestinally-targeted TGR5 agonists equipped with quaternary ammonium have an improved hypoglycemic effect and reduced gallbladder filling effect
Source: Sci Rep. 2016 Jun 24;6:28676. doi: 10.1038/srep28676 (PMC4919643; doi:10.1038/srep28676)

Supplementary Information

Intestinally-targeted TGR5 agonists equipped with quaternary ammonium have an improved hypoglycemic effect and reduced gallbladder filling effect

Hua Cao[a], Zhi-Xiang Chen[a,b], Kai Wang[a], Meng-Meng Ning[a], Qing-An Zou[a], Ying Feng[a], Yang-Liang Ye[a], Ying Leng*[,a] & Jian-Hua Shen*[,a]

[a]State Key Laboratory of Drug Research, Shanghai Institute of Materia Medica (SIMM), Chinese Academy of Sciences, Shanghai 201203, China.

[b]College of pharmacy, Nanchang University, Nanchang 330006, China.

[*]Corresponding author: Y.L. (email: yleng@simm.ac.cn) or J.-H.S (email: jhshen@mail.shcnc.ac.cn).

Content

Part 1. Medicinal chemistry **2**

Part 2. Reference **9**

Part 3. Chemistry **10**

Part 4. Experimental procedure (biological assay) **15**

Part 5. Experimental procedure (chemistry) **20**

Part 6. Synthesis of intermediate 36 and amine derivatives **38**

Part 7. Raw data *in vitro* leading to EC_{50} of 2, 9a, 26a **43**

Part 8. Total cholesterol level after the 18-day treatment of 26a in *ob/ob* mice **45**

Part 9. HPLC purity of the final compounds **46**

Part 10. Copies of ^{1}H NMR, ^{13}C NMR of the final compounds **47**

Part 1. Medicinal chemistry

Our initial goal was to find a new scaffold with higher activity compared with **2** *in vitro*. Extensive explorations of the structure-activity relationship (SAR) of tetrahydroquinoxaline and phenoxy moieties have been done. However, the middle six-membered ring (pyridine of **2**) was thought to be crucial for activity, and few modifications were carried out. In our on-going work, the middle pyridine of **2** was replaced with several five-membered rings. Among them, compound **9a** (Table S1) containing a thiophene ring was the most active. In addition, **9a** exhibited high logP and high liver microsomal clearance (1631/1390 mL/min/g for human/mice), which might decrease its systemic exposure according to studies on non-systemic drugs[1-4]. Therefore, a 3-phenoxythiophene-2-carboxamide scaffold was chosen for further SAR exploration.

On the basis of our initial SAR exploration, the tetrahydroquinoxaline moiety was critical for TGR5 activity and only 1-cyclopropyl-tetrahydroquinoxaline or 1-methyl-tetrahydroquinoxaline was tolerated. Substitutions at positon 4 or 5 of the thiophene ring were detrimental for activity (structure and data not shown). Hence, we focused on the phenoxy side chain and N4 positon of the tetrahydroquinoxaline. Human TGR5 (hTGR5) shares 83% amino acid sequence identity with mouse TGR5 (mTGR5)[5], therefore, hTGR5 and mTGR5 were determined for most compounds.

SAR of the phenoxy side chain and N4 positon of the piperazine ring. Replacement of the pyridine ring of **2** with thiophene increased the activity of hTGR5 and mTGR5 (**9a**,

Table S1). Removal of 2-Cl (**9b**) resulted in considerable reduction in potency, but replacement of 2-Cl of **9a** with 2-Br (**9c**) retained activity. No significant change of activity was found after attachment of an additional 4-Br to **9a** (**9f**). N-c-Pr derivatives displayed a 2–4-fold increase in activity compared with their N-Me derivatives (**9a** *vs.* **9d**, **9b** *vs.* **9e**). According to SAR exploration of compound **1** by Roche, the CF_3 group is beneficial for hTGR5 activity[6]. Therefore, 2,5-di-Cl-phenyl of **9a** and **9d** was replaced with 4-CF_3-Phenyl, but the resulting compound **9g** and **9h** showed decreased activity of hTGR5. Pleasingly, introduction of an additional 2-Br or 2-Cl substitution to **9g** or **9h** elicited a dramatic improvement in potency (**9i-k**), among which **9i** exhibited comparable activity with **9a**. In our previous work, introduction of a methyl group contributed to activity[7]. Therefore, the 2-Br group of **9i** was replaced with 2-methyl, but the corresponding compound **10** displayed a slight decrease in activity. Taken together, **9a**, **9c**, **9f**, and **9i** were the most potent in our series. However, compounds containing Br demonstrated poor solubility (**9c**, **9f**, **9i**, **9j**), which caused difficulty in syntheses and subsequent biological assay *in vivo*. Therefore, **9a** was chosen for further modification.

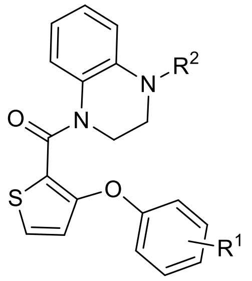

9a-k

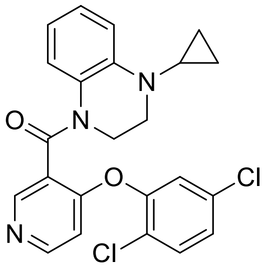

2

Compd	R^1	R^2	hTGR5 EC_{50} (nM)	mTGR5 EC_{50} (nM)
**9a**	2,5-di-Cl	*c*-Pr	0.55 ± 0.18	2.8 ± 0.74
**9b**	3-Cl	c-Pr	26 ± 0.96	–
**9c**	2-Br-5-Cl	*c*-Pr	0.58 ± 0.11	3.0 ± 0.69
**9d**	2,5-di-Cl	Me	2.0 ± 0.35	15 ± 2.9
**9e**	3-Cl	Me	54 ± 15	–
**9f**	4-Br-2,5-di-Cl	*c*-Pr	0.49 ± 0.14	1.7 ± 0.14
**9g**	4-CF_3	*c*-Pr	40 ± 4.0	–
**9h**	4-CF_3	Me	76 ± 9.8	–
**9i**	2-Br-4-CF_3	*c*-Pr	0.68 ± 0.09	1.3 ± 0.22
**9j**	2-Br-4-CF_3	Me	1.7 ± 0.30	5.6 ± 1.3
**9k**	2-Cl-4-CF_3	Me	2.7 ± 0.46	6.9 ± 1.2
**10**	2-methyl-4-CF_3	Me	6.6 ± 1.3	12 ± 1.3
**2**			1.5 ± 0.41	14 ± 6.6

Table S1. SAR exploration of 3-phenoxythiophene-2-carboxamide derivatives. EC_{50} values given are expressed as the mean ± SD of three independent experiments. –, not tested.

However, in a subsequent assay in Institute of Cancer Research (ICR) mice, **9a** increased the gallbladder area by 210% at once a day oral dosing of 50 mg/kg (data not

shown). This side effect was considered to be owing to its moderate membrane permeability (Papp = 0.55 $\times 10^{-6}$cm/s, Table S3), which might be decreased considerably by incorporation of quaternary ammonium. According to the SAR stated above, 2,5-dichloro substitution of the phenyl ring was critical for TGR5 activity, whereas the presence of 4-Br had little effect. Hence, we introduced the quaternary ammonium group to the 4-position of the phenyl ring, and a series of derivatives containing quaternary ammonium were synthesized (Table S2).

SAR of the linkers and substitution of quaternary ammonium derivatives. At first, the role of linker[a] was explored. When the amide was attached directly to the phenyl ring (**20**), TGR5 activity was almost lost compared with **9a**. Pleasingly, extension of linker[a] to a vinyl group (**23**) led to dramatic improvement in activity. Reduction of the double bond afforded the more active TGR5 agonist **26a**, with an EC_{50} of 4.1 and 0.71 nM for hTGR5 and mTGR5, respectively. However, further extension of linker[a] to allyl or propyl (**30** and **33**) resulted in a big decrease in activity. Then, replacement of N-methyl in the amide moiety with an ethyl group (**26b**) resulted in a slight decrease in mTGR5 activity, which suggested that substitution of the amide group with a large group was detrimental to activity.

Next, we turned our attention towards linker[b] and substitution of quaternary ammonium. Prolongation of linker[b] to a propyl group (**26c**) was unfavorable for mTGR5 activity. Cyclization of the amide N atom with quaternary ammonium gave compound

26d with slightly lower activity compared with **26a**. Further placement of quaternary ammonium outside the ring yielded a compound (**26e**) with comparable activity for hTGR5, but its activity in mTGR5 was decreased slightly. Finally, we further introduced high-polarity group (quaternary ammonium or carboxylic acid group) to **26c** and **26a**, which resulted in a large decrease in TGR5 activity (**26f**, **27a–b**).

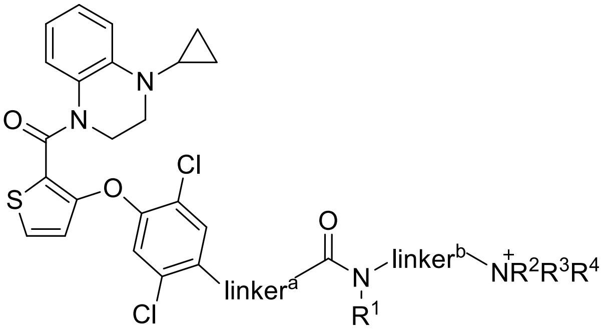

Compd	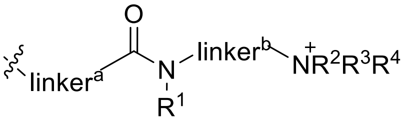	hTGR5 EC_{50} (nM)	mTGR5 EC_{50} (nM)
**20**	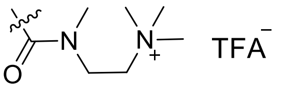	805 ± 217	215 ± 48
**23**	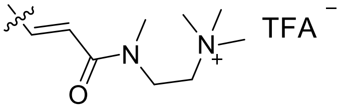	12 ± 1.9	16 ± 2.1
**26a**	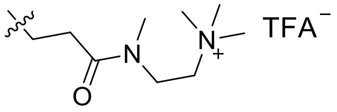	4.1 ± 1.1	0.71 ± 0.08
**26b**	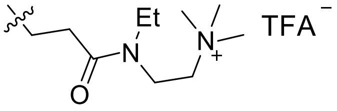	4.7 ± 1.2	3.1 ± 0.35
**26c**	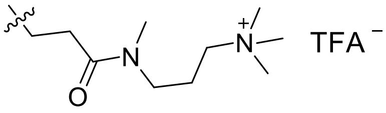	6.6 ± 1.8	4.9 ± 0.41
**26d**	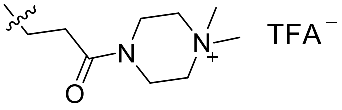	8.7 ± 2.8	1.2 ± 0.16

**26e**	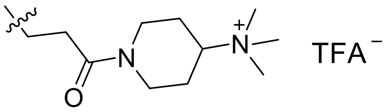	4.5 ± 2.8	2.8 ± 0.29
**26f**	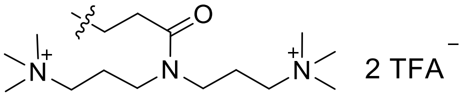	105 ± 9.2	175 ± 72
**27a**	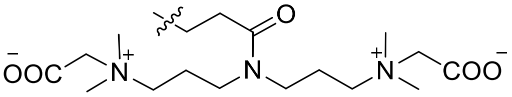	242 ± 29	290 ± 176
**27b**	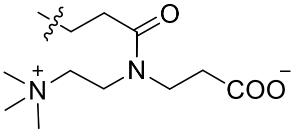	66 ± 31	16 ± 2.4
**30**	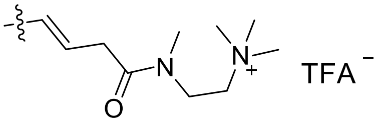	24 ± 4.7	13 ± 1.2
**33**	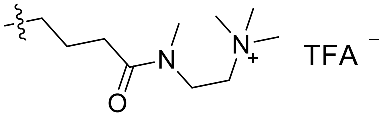	25 ± 2.7	8.7 ± 2.5

Table S2. SAR exploration of the linkers and substitution of quaternary ammonium derivatives. EC_{50} values given are expressed as the mean ± SD of three independent experiments; TFA^- denotes trifluoroacetate, which acts as a negative ion.

26a and **26e** were chosen for Caco-2 cell permeability assay for their best *in vitro* activity, whereas **2** and **9a** were used for comparison. (Table S3) Incorporation of quaternary ammonium into **9a** decreased permeability, especially **26a** (which belonged to a class of low-permeability compounds).

Compd	A to B	B to A	Efflux ratio
	Papp (10^{-6}cm/s)	Papp (10^{-6}cm/s)	

**2**	6.75	5.58	0.8
**9a**	0.55	0.43	0.8
**26a**	0.06	0.8	14.1
**26e**	0.2	0.84	4.1

Table S3. Apparent permeability in Caco-2 Cells of 2, 9a, 26a, and 26e. 'A to B' indicates the experiment from apical to basolateral side, 'B to A' indicates the experiment from basolateral to apical side.

Part 2. Reference

1. Elliott, R. L. *et al.* Discovery of N-benzyl-2-[(4S)-4-(1H-indol-3-ylmethyl)-5-oxo-1-phenyl-4,5-dihydro-6H-[1,2,4]triazolo[4,3-a][1,5]benzodiazepin-6-yl]-N-isopropylacetamide, an orally active, gut-selective CCK1 receptor agonist for the potential treatment of obesity. *Bioorg. Med. Chem. Lett.* **20,** 6797-6801 (2010).

2. Wang, X. Y. *et al.* Highly lipophilic 3-epi-betulinic acid derivatives as potent and selective TGR5 agonists with improved cellular efficacy. *Acta. Pharmacol. Sin.* **35,** 1463-1472 (2014).

3. Filipski, K. J. *et al.* Intestinal targeting of drugs: rational design approaches and challenges. *Curr. Top. Med. Chem.* **13,** 776-802 (2013).

4. Charmot, D. Non-Systemic Drugs: A Critical Review. *Curr. pharm. design* **18,** 1434-1445 (2012).

5. Kawamata, Y. *et al.* A G protein-coupled receptor responsive to bile acids. *J. Biol. Chem.* **278,** 9435-9440 (2003).

6. Martin, R. E. *et al.* 2-Phenoxy-nicotinamides are potent agonists at the bile acid receptor GPBAR1 (TGR5). *ChemMedChem* **8,** 569-576 (2013).

7. Duan, H. *et al.* Design, synthesis, and antidiabetic activity of 4-phenoxynicotinamide and 4-phenoxypyrimidine-5-carboxamide derivatives as potent and orally efficacious TGR5 agonists. *J. Med. Chem.* **55,** 10475-10489 (2012).

Part 3. Chemistry

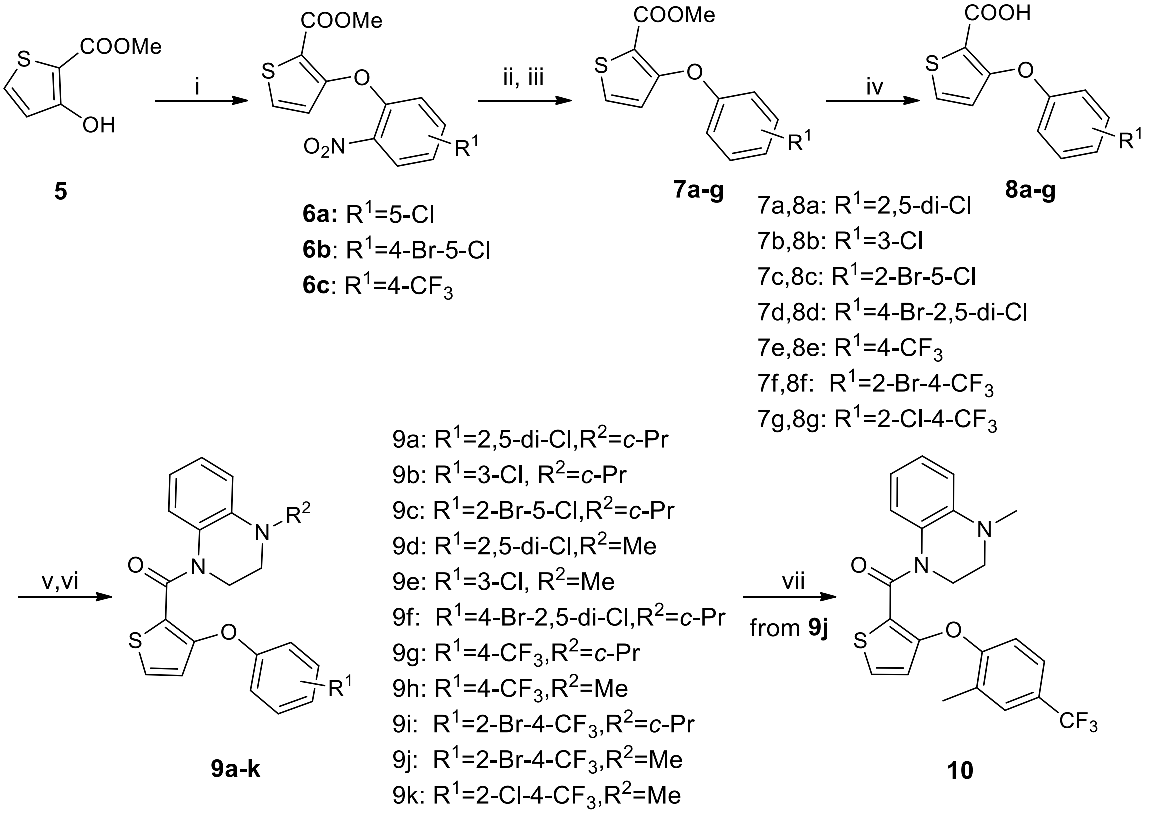

Figure S1. Synthesis of 3-phenoxythiophene-2-carboxamide derivatives. Reagents and conditions: (i) substituted 2-fluoronitrobenzene, K_2CO_3, DMF, rt; (ii) Fe, NH_4Cl, THF/H_2O; (iii) ^{t}BuONO, $CuCl_2$, MeCN, 60 ℃ for **7a, 7d,** and **7g**; ^{t}BuONO, $CuBr_2$, MeCN, 60 ℃ for **7c** and **7f**; ^{t}BuONO, DMF, 80 ℃ for **7b** and **7e**; (iv) NaOH, 1,4-dioxane/H_2O; (v) $(COCl)_2$, DMF, DCM, reflux; (vi) 1-cyclopropyl-1,2,3,4-tetrahydroquinoxaline or 1-methyl-1,2,3,4-tetrahydroquinoxaline, Et_3N, DCM, rt; (vii) $Zn(CH_3)_2$, $Pd(dppf)Cl_2 \cdot CH_2Cl_2$, 1,4-dioxane, 80 ℃.

The synthesis of 3-phenoxythiophene-2-carboxamide scaffold is outlined in Fig. S1. First, substituted 2-fluoronitrobeneze was nucleophilic attacked by hydroxythiophene-2-carboxylate (**5**) to provide the intermediate **6a**–**c**. Reduction of a nitro group with iron powder gave the corresponding amines, which were converted to 2-Cl, 2-Br, or 2-H intermediates **7a**–**g** by diazotization in the presence of $CuCl_2$, $CuBr_2$, or the solvent DMF, respectively. Subsequent hydrolysis of the ester **7a**–**g** yielded acids **8a**–**g**, which were coupled with 1-cyclopropyl-1,2,3,4-tetrahydroquinoxaline or 1-methyl-1,2,3,4-tetrahydroquinoxaline by the acyl chloride formation to afford TGR5 agonists **9a**–**k**. Additionally, the 2-Br-4-CF_3 compound **9j** was converted to 2-methyl-4-CF_3 (**10**) using Pd-catalyzed Negishi coupling.

Benzamide derivative **20** possessing a quaternary ammonium was synthesized following the route depicted in Fig. S2. Compound **5** was protected with a methoxyethoxymethyl (MEM) group followed by hydrolysis to give intermediate **12**. Intermediate **14** was obtained by protection of the carboxylic group with a 4-methoxybenzyl group and deprotection of a MEM group. Subsequent nucleophilic substitution to methyl 2-chloro-4-fluoro-5-nitrobenzoate yielded the 2-nitro intermediate **15**. Reduction of a nitro group with iron power gave the corresponding amine, which was converted to 2-Cl intermediate **16** by diazotization in the presence of $CuCl_2$. Subsequent deprotection of the PMB group with CF_3COOH yielded the acid **17**, which was condensed with 1-cyclopropyl-1,2,3,4-tetrahydroquinoxaline by the acyl chloride

formation to afford the ester **18**. Hydrolysis of ester 18 followed by condensation with N,N,N-trimethyl-2-(methylamino)ethanaminium chloride hydrochloride (**39a**, synthetic procedure available in part 6 of Supplementary Information) afforded the TGR5 agonist **20**.

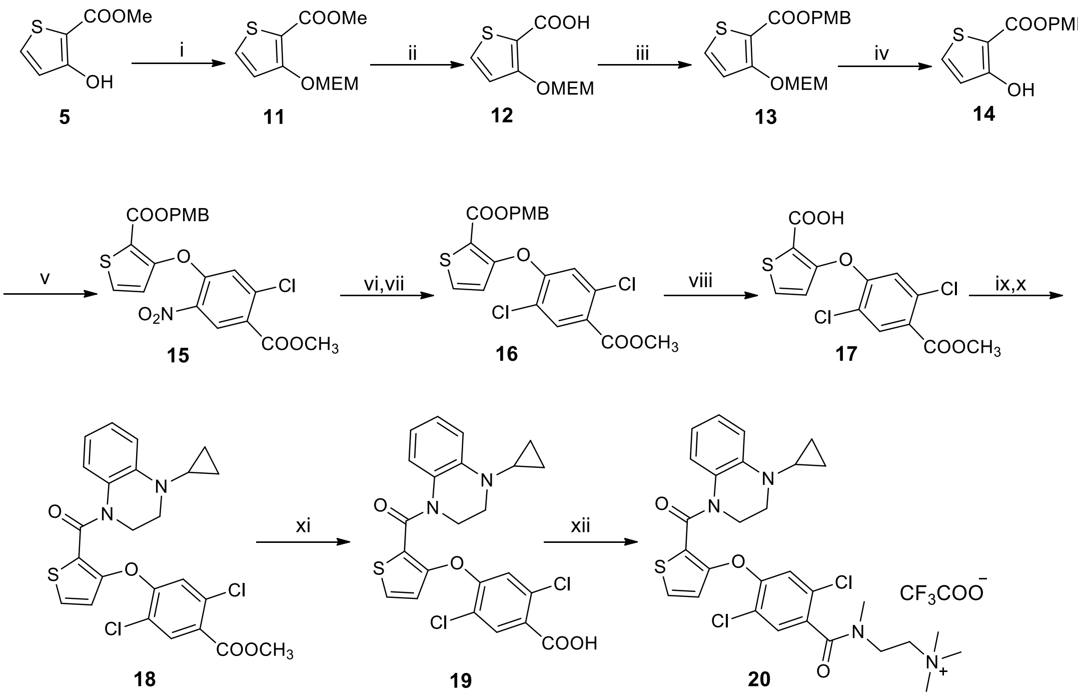

Figure S2. Synthesis of benzamide derivative 20. Reagents and conditions: (i) methoxyethoxymethyl chloride (MEMCl), DIPEA, DCM, rt; (ii) NaOH, MeOH/H_2O,40–50 ℃; (iii) 4-methoxybenzyl chloride (PMBCl), K_2CO_3, DMF, rt; (iv) Amberlyst 15,MeOH, 40–50 ℃ (v) methyl 2-chloro-4-fluoro-5-nitrobenzoate, K_2CO_3, DMF; (vi) Fe, NH_4Cl, THF/H_2O, 60 ℃; (vii) ^{t}BuONO, $CuCl_2$, MeCN, 60 ℃ (viii) CF_3COOH, DCM; (ix) $(COCl)_2$, DMF, DCM, reflux; (x) 1-cyclopropyl-1,2,3,4-tetrahydroquinoxaline, Et_3N, DCM, rt; (xi) NaOH, 1,4-dioxane/H_2O, rt; (xii) **39a**, HATU, DIPEA, DCM.

Preparation of intermediate **21** was accomplished by a microwave-assisted Heck reaction of **9f** with ethyl acrylate (Fig. S3). Subsequent hydrolysis and condensation with **39a** afforded cinnamamide derivatives **23**. The olefin moiety of intermediate **21** could be reduced with sodium borohydride ($NaBH_4$) and one equivalent copper(I) chloride to give **24**. Further hydrolysis and condensation with different amines afforded phenylpropanamide derivatives **26a–i**. Saponification of **26g–i** provided **27a–c**.

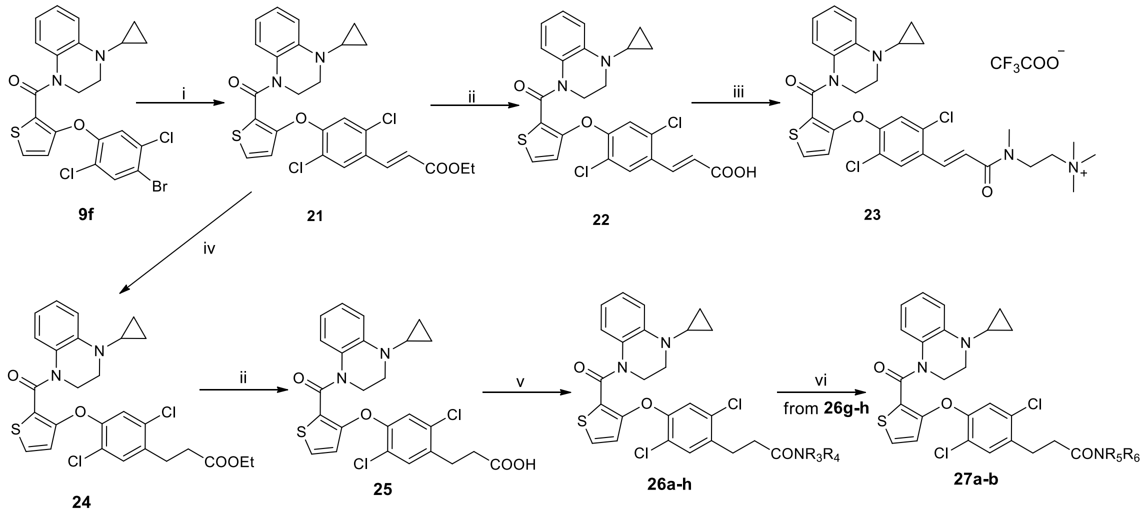

Figure S3. Synthesis of cinnamamide and phenylpropanamide derivatives containing quaternary ammonium. Reagents and conditions: (i) ethyl acrylate, $Pd(OAc)_2$, $P(o\text{-}MePh)_3$, LiCl, Et_3N, MeCN, microwave 140 ℃; (ii) NaOH, 1,4-dioxane/H_2O, rt; (iii) **39a**, HATU, DIPEA, DCM; (iv) $NaBH_4$, CuCl, MeOH/THF, 0 ℃; (v) amines, HATU, DIPEA, DCM, rt; (vi) NaOH, 1,4-dioxane/H_2O, rt.

The synthesis of (E)-4-phenylbut-3-enamide and phenylbutanamide derivatives is shown in Fig. S4. Heck reaction of **9f** with isobutyl vinylacetate gave intermediate **28**,

which could be hydrogenated to **31**. Hydrolysis of **28** and **31** and further condensation with **39a** yielded **30** and **33**, respectively.

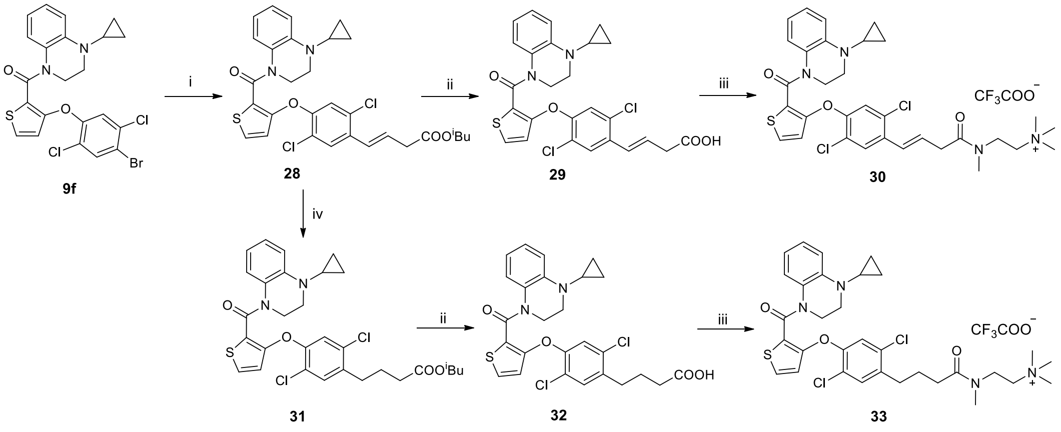

Figure S4. Synthesis of (E)-4-phenylbut-3-enamide and phenylbutanamide derivatives with quaternary ammonium. Reagents and conditions: (i) isobutyl vinylacetate, $Pd(OAc)_2$, $P(o\text{-}MePh)_3$, LiCl, Et_3N, MeCN, microwave 140 ℃; (ii) NaOH, 1,4-dioxane/H_2O, rt; (iii) **39a**, HATU, DIPEA, DCM; (iv) H_2, Pd/C, MeOH, rt.

Part 4. Experimental procedure (biological assay)

***In vitro* TGR5 assay.** *In vitro* TGR5 assay was carried out according to (*J. Med. Chem.* **2012**, *55*,10475-10489). hTGR5/CRE/HEK293 or mTGR5/CRE/HEK293 stable cell line was obtained by transfection of HEK293 cells with human or mouse TGR5 expression plasmid (hTGR5-pcDNA3.1 or mTGR5-pcDNA3.1) and CRE-driven luciferase reporter plasmid (pGL4.29, Promega, Madison, WI, USA) and employed to assess the activity of test compounds by reporter gene assay. Briefly, cells were seeded into 96-well plates and incubated overnight in DMEM supplemented with 10% FBS in 5% CO_2 at 37 ℃. Then, cells were incubated with fresh medium containing different concentrations of test compounds or 20 μM INT-777 as a positive control for 5.5 h. Luciferase activity in cell lysate was determined using the Steady-Glo Luciferase Assay System (Promega) according to the manufacturer's instructions.

FXR Assay. FXR activation was tested using a LanthaScreen® TRFRET Farnesoid X Receptor Coactivator Assay Kit. All of the procedures followed the manufacturer's instructions. The kit uses a terbium (Tb)-labeled anti-GST antibody, a fluorescein-labeled coactivator peptide, and an FXR ligand-binding domain (FXR-LBD) that is tagged with glutathione-S-transferase (GST), in a homogeneous, mix-and-read assay format.

Animals. Male ICR mice (7-8 weeks) were purchased from the Shanghai SLAC Laboratory Animal Co. Ltd. (Shanghai, China). B6.Cg-*Lep*^{ob}/J (*ob*/*ob*) mice (from Jackson Laboratory, Bar Harbor, ME, USA, stock number 000632) were bred at the Shanghai Institute of Materia Medica (SIMM), Chinese Academy of Sciences. The animals were maintained under a 12 h light−dark cycle with free access to water and food. Animal experiments were carried out according to the Guidelines for the Care and Use of Laboratory Animals and were approved by the Animal Care and Use Committee, Shanghai Institute of Materia Medica, Chinese Academy of Sciences.

Experimental method for LC-MS/MS:

MS instrument: Waters Xevo TQ-S MS/MS; Sofware: Masslynx 4.1 (Waters); Source : ESI; Scan Mode: MRM; Polarity:Positive; Ionspray Voltage: 4200 V; Temperature: 400 ℃; Declustering potential: 110 V.

Observed masses: Compound **2**: m/z 440.0 m/z 278.2 amu; Compound **9a**: m/z 445.1 m/z 270.9 amu; Compound **26a**: m/z 615.0 m/z 556.0 amu; Compound **26e**: m/z 641.3 m/z 84.1, 325.0 amu.

LC separation: Analytical column: Waters Acquity UPLC BEH C18 (1.7 μm, 2.1 × 50 mm); Mobile phase: A: 5 mM ammonium acetate (containing 0.1% formic acid); B:

Acetonitrile (containing 0.1% formic acid; C (MeOH with 1% TFA). Flow rate: 0.6 mL/min.

Time	A%	B%	C%	Curve
Initial	40	15	45	Initial
0.8	10	15	75	6
1.7	5	15	80	6
2.3	40	15	45	1

Table S4. Gradient for compound 2 and 9a

Time	A%	B%	C%	Curve
Initial	50	10	40	Initial
0.8	20	10	70	6
1.7	10	10	80	6
2.3	50	10	40	1

Table S5. Gradient for compound 26a and 26e

Data system: Masslynx 4.1 (Waters).

***In vitro* microsomal stability assay.** The test compound was dissolved in DMSO and diluted to the desired concentration with DMSO and 0.1% aqueous BSA. Liver microsomes were incubated in a 96-well plate containing 0.1 M Tris buffer (pH 7.4), 0.33 mg/mL microsomal protein, 0.1 mM test compound, 5.0 mM $MgCl_2$, 0.005% BSA, and 1.0 mM NADPH. Incubations were conducted at 37 ℃. An aliquot was removed at each time point and the enzymatic reaction was stopped by protein precipitation in MeOH. The loss of the test compound was determined by LC-MS/MS. The intrinsic clearance (CL_{int}, mL/min/g) was calculated from the following equation:

$$CL_{int} = 1000 \times (-k)/p$$

Where k (min^{-1}) is the slope of the line of the natural log of the percentage parent remaining versus the time of incubation; P is the microsomal protein concentration (mg/mL).

Caco-2 permeability determination. Caco-2 permeability determination was carried out according to (*Eur. J. Med. Chem.* **2014**, *82*, 1-15). Caco-2 cells were obtained from the ATCC (Cat#HTB-37) and maintained in Dulbecco's modified Eagle's Medium containing 10% fetal bovine serum, 1% glutamine, 1% nonessential amino acids, 100 μg/mL streptomycin, and 100 U/mL penicillin. Caco-2 cells were cultured at 37 ℃ in a 5% CO_2 and 90% relative humidity environment. Caco-2 cells were passaged every 7 days at a ratio of 1:10. Cells were used between passages 30 and 40. After 21 days of culture, the integrity of the cell monolayer was verified by measuring the transepithelial electrical resistance (TEER).

Drug transport from the apical side to the basolateral side (A-B) and from the basolateral side to the apical side was measured simultaneously under the same condition. Propranolol and atenolol were used as the hypertonic and hypotonic control, respectively. Digoxin was used as the positive control for Pgp-mediated drug efflux. In brief, the method was as follows. After washing the monolayer with HBSS three times, the compounds were diluted and added to the appropriate well (pH 6.8 for apical side and pH 7.4 for basolateral side). The plate was incubated at 37 ℃ for 95 min. Samples were collected from the donor side at 5 and 95 min, and from the receiver side at 35 and 95 min post-incubation. The concentration of samples was measured by LC-MS/MS. The P_{app} was calculated from the following equation:

$$P_{app} = (V_A/(SA \times T)) \times ([drug]_{acceptor} / [durg]_{initial\ donor})$$

Where V_A is the volume of the acceptor well, *SA* is the surface area of the membrane, *T* is the total transport time, $[drug]_{acceptor}$ is the drug level at the acceptor side, and [drug]initial donor is the drug level at the donor side at $T = 0$.

Oral glucose tolerance test (OGTT) and gallbladder filling assay in ICR mice. 100 mg/kg of **26a** or 0.25% CMC (control) was administered orally to overnight-fasted ICR mice (n = 7-8) 90 min prior to the oral glucose load (4 g/kg). Blood glucose levels were measured via blood drops obtained by clipping the tail of the mice using an Accu-Chek Advantage II Glucose Monitor (Roche, Indianapolis, IN, USA) before compound dosing and 0, 15, 30, 60, and 120 min after the glucose load. The area under the concentration−time curve from 0 to 120 min ($AUC_{0-120\ min}$, Glu) of blood glucose after the glucose load was calculated by the trapezoidal rule.

After the OGTT experiment, the fasted mice were refed for 3 h, gallbladders were then removed, and the area was measured using vernier caliper. The relative gallbladder area was calculated from the length multiplied by the width of the gallbladder. The bile weight was measured using Analytical Balances.

Acute efficacy in *ob*/*ob* mice. 7-week-old *ob*/*ob* mice (male) were divided into three groups (n = 8) based on 2 h-fasted blood glucose. Compound **2** (50 mg/kg), **26a** (100 mg/kg) and 0.25% CMC (control) was then orally administered to 2 h-fasted mice, and blood glucose was measured at 2, 4, 6, 8, 10, and 24 h after dosing. The animals were refed at 6 h after dosing.

GLP-1 secretion in *ob*/*ob* mice. The *ob*/*ob* mice (female) were divided into 4 groups and treated with 0.25% CMC (control), **26a** (100 mg/kg), DPP-4 inhibitor Linagliptin (3 mg/kg), **26a** (100 mg/kg) plus Linagliptin (3 mg /kg), while the latter three groups were then divided into 3 subgroup of different time point (n = 8-9 each subgroup). All the animals were fasted for 6 hours before collecting blood samples at the indicated time points. At other times, animals were free accessed to water and food. 6 h, 12 h, 24 h later, blood samples were collected and placed in Eppendorf tubes containing the DPP-4 inhibitor valine pyrrolidide (Linco Research, DPP-4-010) with a final concentration of 1% blood samples and 25 mg/mL EDTA. Concentrations of active GLP-1[7-36 amide] in the serum were measured using ELISA kit from Linco Research. All time point shared the same control group collected after 12 h.

Pharmacokinetics assay of 26a in *ob*/*ob* mice. Compound **26a** (100 mg/kg) was orally administered to 12 h-fasted *ob*/*ob* mice (n = 3 per time point). Blood and intestinal tissue samples were collected before dosing or 2 h, 4 h, 6 h, 10 h 16 h, 24 h after dosing. Blood samples were centrifuged at 11000 rpm for 5 min to isolate the plasma. 25 μL of the sample was mixed with 75 μL MeOH. After centrifugation at 11000 rpm for 5 mins, the supernatant was analyzed by LC-MS/MS.

The tissues were ground and homogenated with 0.9% NaCl solution (1:4, w/v), and then the homogenates were centrifuged at 12,000 rpm for 10 min at 4 ℃. The supernatant was collected, and 100 μL aliquot of supernatant was spiked with 300 μL methanol, and then treated as the same fashion as plasma sample. Density of intestinal tissue was taken as 1 g/ml.

Gallbladder filling assay in *ob*/*ob* mice. 100 mg/kg of **26a** or 0.25% CMC (control) was administered orally to overnight-fasted *ob*/*ob* mice (male, n = 3-6). 1 h after dosing, mice were refed for 3 hours, and then gallbladders were removed, and the area was measured using vernier caliper. The relative gallbladder area was calculated from the length multiplied by the width of the gallbladder. The bile weight was measured using Analytical Balances.

While in the three days gallbladder filling assay of **26a**, 2 groups of *ob*/*ob* mice were administered to 100 mg/kg of **26a** or 0.25% CMC (control) for 3 days, while the following procedure were the same as the once a day dosing assay. Blood, bile and gallbladder tissue samples in this assay were collected for further drug level test.

Drug levels test in plasma, bile, and gallbladder tissue after 3 days oral administration to *ob/ob* mice. Plasma, bile, and gallbladder were taken from experiments performed in accordance with the guidelines for the use of experimental animals in the Shanghai Laboratory Animal Administration (Shanghai, China) after three days treatment of **26a** to *ob*/*ob* mice. Blood samples were collected through cardiac puncture into heparinized tubes, and plasma was harvested by centrifugation. Bile and gallbladder tissue were collected from each mouse after blood withdrawal. To 10 mg of gallbladder was added 100 μL of MeOH, and the mixture was homogenized. A 15 μL aliquot of bile samples was diluted with 100 μL of MeOH and then mixed. Plasma, bile, and tissue homogenate were stored at −20 °C. These samples were protein precipitated with MeCN using clopidogrel as the internal standard, and the supernatant was injected onto a LC-MS/MS system for the quantification of **26a**. Chromatographic separation was performed on a Waters Acquity UPLC BEH C18 (1.7 μm, 2.1 × 50 mm) at a flow rate of 0.5 mL min^{-1} using ternary mobile phase: A (H_2O with 5 mM ammonium acetate and 0.5% TFA); B(MeCN: H_2O = 95:5 with 0.1% TFA); C (MeOH with 1% TFA). The MS/MS detection was carried out in MRM mode using a positive electrospray ionization interface.

Long-term study of 26a in *ob/ob* mice. Three groups of *ob*/*ob* mice (male) was orally administered to **26a** (50 mg/kg), **26a** (100 mg/kg) and 0.25% CMC (control) for 18 days. At day 0 (before dosing), 4, 8, 12 and 18, non-fasted blood glucose was measured before dosing. Mice were fasted for 6 h after dosing and then fasted blood glucose was measured. The serum level of HbA_{1c} was measured at day 0 (before dosing) and 18 by Adicon biochemical analyzer. The body weight of each mouse was recorded every day. The triglyceride and total cholesterol level was measured after the final dose.

Statistical analysis. All data were expressed as the mean ± SEM or mean ± SD. For the experiment including multiple time points, one-way ANOVA statistical analysis was used; otherwise, unpaired Student's *t* test was used. $P < 0.05$ was considered to be statistically significant.

Part 5. Experimental procedure (chemistry)

Synthetic materials and methods. All reagents were purchased from commercial suppliers and used without further purification. Microwave reactions were performed in a Biotage Initiator. Column chromatography was carried out on silica gel (200-300 mesh) or with pre-packed silica cartridges (4-40 g) from Bonna-Agela Technologies Inc. (Tianjin, China) and eluted with a CombiFlash@ Rf 200 from Teledyne Isco. Prep-HPLC separation was carried out in Unimicro Easysep-1010 series LC(UV 254 nM, 25 ℃, flow rate = 10 mL min^{-1}) with the column of Agilent Prep-C18 10 μm, 21.2 × 250 mm, while the mobile phase was 30-90% MeCN/H_2O (containing 0.1%TFA) in 1h. Melting point (mp) of target compounds was measured by SGWX-4 melting point apparatus (Shanghai Precision and Scientific Instrument Corporation, Shanghai, China). ^1H NMR and ^{13}C NMR spectra were recorded on a Varian-Mercury Plus-300 or a Bruker Avance III 400 or a Bruker Avance III 500 NMR spectrometer using tetramethylsilane or solvent signals as an internal reference. IR spectra were recorded on IS5 FT-IR (Thermo). High-resolution mass spectra (ESI) were obtained on a Q-TOF or Thermo Orbitrap Elite. The purity of tested compounds was determined by HPLC (Agilent LC1260, Agilent ChemStation, ZORBAX SB-C18, 5 μm, 4.6 × 150 mm, UV 254 nM, 30 ℃, flow rate = 1.0 mL min^{-1}). All of the assayed compounds possess >95% purity.

Methyl 3-hydroxythiophene-2-carboxylate (5). It was prepared according to (*Pestic. Sci*. **1996**, *48*, 351-358). ^1H NMR (300 MHz, $CDCl_3$): δ 9.57 (s, 1H), 7.37 (d, J = 5.7 Hz, 1H), 6.74 (d, J = 5.7 Hz, 1H), 3.89 (s, 3H).

General procedure for preparation of compound 6a–c. To a solution of **5** (1.20 g, 7.59 mmol) in dry DMF (25 mL) was added K_2CO_3 (1.15 g， 8.32 mmol) and the corresponding substituted 2-fluoronitrobenzene (7.59 mmol). The reaction mixture was stirred at room temperature overnight and then poured into water (50 mL) and extracted with ethyl acetate for three times. Organic layers were combined, washed with saturated brine for three times, dried over anhydrous magnesium sulfate and evaporated under vacuum. The residue was purified by flash column chromatography to yield the desired compound.

Methyl 3-(5-chloro-2-nitrophenoxy)thiophene-2-carboxylate (6a). The title compound was synthesized from **5** and 4-chloro-2-fluoro-1-nitrobenzene in yield of 85%. ^1H NMR (300 MHz, $CDCl_3$): δ 7.48 (d, J = 5.4 Hz, 1H), 7.38 (d, J = 8.4 Hz, 1H), 7.05 (dd, J = 8.4, 2.4 Hz, 1H), 6.88 (d, J = 2.4 Hz, 1H), 6.70 (d, J = 5.4 Hz, 1H), 3.82 (s, 3H).

Methyl 3-(4-bromo-5-chloro-2-nitrophenoxy)thiophene-2-carboxylate (6b). The title compound was synthesized from **5** and 1-bromo-2-chloro-4-fluoro-5-nitrobenzene (prepared according to US6331553 B1) in yield of 78%. ^1H NMR (400 MHz, $CDCl_3$): δ 7.71 (s, 1H), 7.51 (d, J = 5.4 Hz, 1H), 6.95 (s, 1H), 6.74 (d, J = 5.4 Hz, 1H), 3.82 (s, 3H).

Methyl 3-(2-nitro-4-(trifluoromethyl)phenoxy)thiophene-2-carboxylate (6c). The title compound was synthesized from **5** and 1-fluoro-2-nitro-4-(trifluoromethyl)benzene in yield of 88%. ^{1}H NMR (300 MHz, $CDCl_3$): δ 8.27 (d, J = 1.8 Hz, 1H), 7.71 (dd, J = 8.8, 1.8 Hz, 1H), 7.60 (d, J = 5.4 Hz, 1H), 6.99 (d, J = 8.8 Hz, 1H), 6.95 (d, J = 5.4 Hz, 1H), 3.75 (s, 3H).

General procedure for preparation of compound 7a–g. To a solution of the 2-nitro benzene intermediate **6a–c** (1.06 mmol) in THF (50 mL) and H_2O (50 mL) was added iron power (0.47 g, 8.4 mmol) and NH_4Cl (113 mg, 2.11 mmol). The resulting mixture was stirred at 65 ℃ overnight. Then the reaction mixture was cooled to room temperature, filtered and the filtrate evaporated in vacuum, diluted with water and extracted with ethyl acetate for three times. Organic layers were combined, washed with saturated brine for three times, dried over anhydrous magnesium sulfate and evaporated under vacuum to yield the crude product which was subjected to the next step without further purification.

A solution of the crude product obtained above in MeCN or DMF was added $CuCl_2$ (2.12 mmol) and ^{t}BuONO (2.12 mmol), or $CuBr_2$ (2.12 mmol) and ^{t}BuONO (2.12 mmol), or ^{t}BuONO (2.12 mmol). The resulting mixture was stirred at room temperature for 0.5 h and then heated at 60 ℃ or 80 ℃ overnight. Then the reaction mixture was cooled to room temperature and evaporated in vacuum, diluted with water and extracted with ethyl acetate for three times. Organic layers were combined, washed with saturated brine for three times, dried over anhydrous magnesium sulfate and evaporated under vacuum. The residue was purified by flash column chromatography to yield the desired compound.

Methyl 3-(2,5-dichlorophenoxy)thiophene-2-carboxylate (7a). The title compound was synthesized from **6a** with $CuCl_2$ and ^{t}BuONO in MeCN at 60 ℃ in yield of 68%. ^{1}H NMR (300 MHz, $CDCl_3$): δ 7.48 (d, J = 5.4 Hz, 1H), 7.38 (d, J = 8.4 Hz, 1H), 7.05 (dd, J = 8.4, 2.4 Hz, 1H), 6.88 (d, J = 2.4 Hz, 1H), 6.70 (d, J = 5.4 Hz, 1H), 3.82 (s, 3H).

Methyl 3-(3-chlorophenoxy)thiophene-2-carboxylate (7b). The title compound was synthesized from **6a** with ^{t}BuONO in DMF at 80 ℃ in yield of 73%.^{1}H NMR (300 MHz, $CDCl_3$): δ 7.45 (d, J = 5.4 Hz, 1H), 7.26 (m, 1H), 7.09 (m, 1H), 7.02 (t, J = 2.1 Hz, 1H), 6.93 (dd, J = 8.4, 1.5 Hz, 1H), 6.72 (d, J = 5.4 Hz, 1H), 3.82 (s, 3H)

Methyl 3-(2-bromo-5-chlorophenoxy)thiophene-2-carboxylate (7c). The title compound was synthesized from **6a** with $CuBr_2$ and ^{t}BuONO in MeCN at 60 ℃ in yield of 65%. ^{1}H NMR (300 MHz, $CDCl_3$): δ 7.54 (d, J = 8.4 Hz, 1H), 7.48 (d, J = 5.4 Hz, 1H), 6.99 (dd, J = 8.4, 2.4 Hz, 1H), 6.84 (d, J = 2.4 Hz, 1H), 6.71 (d, J = 5.4 Hz, 1H), 3.82 (s, 3H).

Methyl 3-(4-bromo-2,5-dichlorophenoxy)thiophene-2-carboxylate (7d). The title compound was synthesized from **6b** with $CuCl_2$ and ^{t}BuONO in MeCN at 60 ℃ in yield

of 68%. ^{1}H NMR (400 MHz, $CDCl_3$): δ 8.27 (s, 1H), 7.59 (d, J = 5.4 Hz, 1H), 6.99 (s, 1H), 6.92 (d, J = 5.4 Hz, 1H), 3.78 (s, 3H).

Methyl 3-(4-(trifluoromethyl)phenoxy)thiophene-2-carboxylate (7e). The title compound was synthesized from **6c** with ^{t}BuONO in DMF at 80 ℃ in yield of 79%. ^{1}H NMR (300 MHz, $CDCl_3$): δ 7.58 (d, J = 8.7 Hz, 2H), 7.49 (d, J = 5.7 Hz, 1H), 7.07 (d, J = 8.7 Hz, 2H), 6.77 (d, J = 5.7 Hz, 1H), 3.80 (s, 3H)

Methyl 3-(2-bromo-4-(trifluoromethyl)phenoxy)thiophene-2-carboxylate (7f). The title compound was synthesized from **6c** with $CuBr_2$ and ^{t}BuONO in MeCN at 60 ℃ in yield of 65%. ^{1}H NMR (400 MHz, $CDCl_3$): δ 7.90 (d, J = 1.8 Hz, 1H), 7.53 (d, J = 5.4 Hz, 1H), 7.47 (m, 1H), 6.86 (d, J = 8.6 Hz, 1H), 6.79 (d, J = 5.4 Hz, 1H), 3.79 (s, 3H).

Methyl 3-(2-chloro-4-(trifluoromethyl)phenoxy)thiophene-2-carboxylate (7g). The title compound was synthesized from **6c** with $CuCl_2$ and ^{t}BuONO in MeCN at 60 ℃ in yield of 63%. ^{1}H NMR (400 MHz, $CDCl_3$): δ 7.74 (d, J = 1.9 Hz, 1H), 7.52 (d, J = 5.4 Hz, 1H), 7.43 (m, 1H), 6.91 (d, J = 8.6 Hz, 1H), 6.78 (d, J = 5.4 Hz, 1H), 3.80 (s, 3H).

General procedure for preparation of compound 8a-g. To a solution of **7a–g** (0.66 mmol) in 1,4-dioxane (10 mL) and H_2O (10 mL) was added NaOH (1.32 mmol). The resulting mixture was stirred at room temperature overnight. The reaction solution was then concentrated under vacuum, diluted with water, acidified with HCl (4N). The solid was filtered and dried at 45 ℃ overnight to afford the desired acid **8a–g**.

3-(2,5-Dichlorophenoxy)thiophene-2-carboxylic acid (8a). The title compound was synthesized from **7a** in yield of 93%. ^{1}H NMR (300 MHz, DMSO-d_6): δ 13.12 (br, 1H), 7.60 (d, J = 7.2 Hz, 1H), 7.42 (d, J = 5.4 Hz, 1H), 7.30 (d, J = 1.2, 7.2 Hz, 1H), 7.12 (d, J = 1.2 Hz, 1H), 6.76 (d, J = 5.4 Hz, 1H)

3-(3-Chlorophenoxy)thiophene-2-carboxylic acid (8b). The title compound was synthesized from **7b** in yield of 91%. ^{1}H NMR (300 MHz, $CDCl_3$): δ 7.37 (d, J = 5.4 Hz, 1H), 7.17 (d, J = 8.1 Hz, 1H), 6.99 (m, 1H), 6.95 (t, J = 2.4 Hz, 1H), 6.86 (ddd, J = 8.1, 2.4, 0.9 Hz, 1H), 6.62 (d, J = 5.4 Hz, 1H)

3-(2-Bromo-5-chlorophenoxy)thiophene-2-carboxylic acid (8c). The title compound was synthesized from **7c** in yield of 89%. ^{1}H NMR (300 MHz, DMSO-d_6): δ 12.98 (br, 1H), 7.67 (d, J = 7.2 Hz, 1H), 7.45 (d, J = 5.4 Hz, 1H), 7.33 (d, J = 7.2, 1.2 Hz, 1H), 7.16 (d, J = 1.2 Hz, 1H), 6.84 (d, J = 5.4 Hz, 1H).

3-(4-Bromo-2,5-dichlorophenoxy)thiophene-2-carboxylic acid (8d). The title compound was synthesized from **7d** in yield of 95%. ^{1}H NMR (400 MHz, DMSO-d_6): δ 8.14 (s, 1H), 7.96 (d, J = 5.0 Hz, 1H), 7.23 (s, 1H), 7.00 (d, J = 5.4 Hz, 1H).

3-(4-(Trifluoromethyl)phenoxy)thiophene-2-carboxylic acid (8e). The title compound was synthesized from **7e** in yield of 86%. ^{1}H NMR (300 MHz, $CDCl_3$): δ 7.61 (d, J = 8.6 Hz, 2H), 7.56 (d, J = 5.5 Hz, 1H), 7.11 (d, J = 8.5 Hz, 2H), 6.74 (d, J = 5.5 Hz, 1H).

3-(2-Bromo-4-(trifluoromethyl)phenoxy)thiophene-2-carboxylic acid (8f). The title compound was synthesized from **7f** in yield of 94%. ^{1}H NMR (300 MHz, $CDCl_3$): δ 7.74 (s, 1H), 7.57 (d, J = 5.4 Hz, 1H), 7.46 (d, J = 8.7 Hz, 1H), 7.01 (d, J = 8.7 Hz, 1H), 6.69 (d, J = 5.4 Hz, 1H)

3-(2-Chloro-4-(trifluoromethyl)phenoxy)thiophene-2-carboxylic acid (8g). The title compound was synthesized from **7g** in yield of 89%. ^{1}H NMR (300 MHz, $CDCl_3$): δ 7.90 (d, J = 1.5 Hz, 1H), 7.57 (d, J = 5.4 Hz, 1H), 7.51 (dd, J = 1.5, 8.7 Hz, 1H), 6.97 (d, J = 8.7 Hz, 1H), 6.70 (d, J =5.4 Hz, 1H)

General procedure for preparation of compound 9a–k. To a solution of **8a–g** (3 mmol) in DCM was added DMF (3 drops) and $(COCl)_2$ (9 mmol). The resulting mixture was refluxed for 3 h. After the reaction was cooled to room temperature, the solvent and $(COCl)_2$ was removed under vacuum. Then the residue was dissolved in DCM before adding Et_3N (9 mmol) and 1-cyclopropyl-1,2,3,4-tetrahydroquinoxaline (3 mmol) or 1-methyl-1,2,3,4-tetrahydroquinoxaline (3 mmol). The resulting mixture was stirred at room temperature overnight, diluted with water, extracted with DCM. The organic layer was combined, washed with saturated brine for three times, dried over anhydrous magnesium sulfate, and evaporated under vacuum. The residue was purified by flash column chromatography to provide **9a–k** as target compounds.

(4-Cyclopropyl-3,4-dihydroquinoxalin-1(2H)-yl)(3-(2,5-dichlorophenoxy)thiophen-2-yl)methanone (9a). The title compound was obtained as a white solid from **8a** and 1-cyclopropyl-1,2,3,4-tetrahydroquinoxaline in yield of 76%. Mp 120 ℃. ^{1}H NMR (400 MHz, $CDCl_3$): δ 7.37 (d, J = 5.6 Hz, 1H), 7.21 (d, J = 8.0 Hz, 1H), 6.89 (m, 2H), 6.81 (d, J = 7.6 Hz, 1H), 6.74 (d, J = 7.6 Hz, 1H), 6.48 (dt, J = 7.6, 1.6 Hz, 1H), 6.44 (d, J = 5.2 Hz, 1H), 6.07 (s, 1H), 3.94 (t, J = 5.6 Hz, 2H), 3.47 (t, J = 5.2 Hz, 2H), 2.27 (m, 1H), 0.70 (m, 2H), 0.40 (m, 2H). ^{13}C NMR (126 MHz, $CDCl_3$): δ 160.59, 152.27, 148.91, 139.58, 132.80, 130.51, 128.00, 126.16, 125.41, 123.96, 123.00, 122.08, 121.86, 118.80, 117.95, 115.75, 112.98, 48.90, 41.18, 31.16, 7.90. IR (KBr) cm^{-1}: 1622.60, 1507.34, 1472.43, 1435.85, 1409.73, 1320.27, 1241.68. HRMS (ESI^{+}) m/z calcd for $C_{22}H_{19}Cl_2N_2O_2S^{+}$ $(M + H)^{+}$ 445.0539, found 445.0526.

(3-(3-Chlorophenoxy)thiophen-2-yl)(4-cyclopropyl-3,4-dihydroquinoxalin-1(2H)-yl) methanone (9b). The title compound was obtained as a light yellow solid from **8b** and 1-cyclopropyl-1,2,3,4-tetrahydroquinoxaline in yield of 82%. Mp 109 ℃. ^{1}H NMR (300 MHz, $CDCl_3$): δ 7.35 (d, J = 5.4 Hz, 1H), 7.09 (t, J = 8.0 Hz, 1H), 6.97 (m, 3H), 6.75 (d, J = 7.8 Hz, 1H), 6.48 (m, 4H), 3.92 (m, 2H), 3.28 (m, 2H), 2.30 (tt, J = 6.6, 3.7 Hz, 1H),

0.74 (td, J = 6.8, 4.8 Hz, 2H), 0.42 (m, 2H). ^{13}C NMR (126 MHz, $CDCl_3$): δ 160.82, 157.31, 149.82, 139.77, 134.69, 130.11, 127.90, 125.94, 125.89, 123.38, 122.78, 121.65, 119.56, 117.67, 116.11, 115.20, 113.04, 48.86, 41.39, 31.21, 7.95. IR (KBr) cm^{-1}: 1629.54, 1594.72, 1504.45, 1472.78, 1439.10, 1405.54, 1361.30, 1321.19, 1247.91, 1228.90, 741.76. HRMS (ESI$^+$) m/z calcd for $C_{22}H_{19}ClN_2NaO_2S^+$ (M + Na)$^+$ 433.0748, found 433.0759.

(3-(2-Bromo-5-chlorophenoxy)thiophen-2-yl)(4-cyclopropyl-3,4-dihydroquinoxalin-1(2H)-yl)methanone (9c). The title compound was obtained as a light yellow solid from **8c** and 1-cyclopropyl-1,2,3,4-tetrahydroquinoxaline in yield of 78%. Mp 107 ℃. ^{1}H NMR (300 MHz, DMSO-d_6): δ 7.76 (d, J = 5.5 Hz, 1H), 7.59 (d, J = 8.5 Hz, 1H), 7.03 (dd, J = 8.5, 2.3 Hz, 1H), 6.82 (td, J = 7.9, 7.3, 1.4 Hz, 1H), 6.71 (m, 1H), 6.65 (m, 2H), 6.42 (m, 1H), 5.89 (s, 1H), 3.79 (t, J = 4.9 Hz, 2H), 3.40 (m, 2H), 2.23 (dq, J = 6.3, 3.3 Hz, 1H), 0.66 (m, 2H), 0.28 (m, 2H). ^{13}C NMR (126 MHz, $CDCl_3$): δ 160.57, 153.34, 148.97, 139.56, 133.62, 133.44, 127.95, 126.19, 125.38, 124.41, 123.03, 122.06, 118.75, 117.89, 115.67, 112.95, 110.40, 48.95, 41.07, 31.21, 7.93. IR (KBr) cm^{-1}: 1621.72, 1502.03, 1467.40, 1432.18, 1402.89, 1235.25, 745.29. HRMS (ESI$^+$) m/z calcd for $C_{22}H_{18}BrClN_2NaO_2S^+$ (M + Na)$^+$ 510.9853, found 510.9850.

(3-(2,5-Dichlorophenoxy)thiophen-2-yl)(4-methyl-3,4-dihydroquinoxalin-1(2H)-yl)methanone (9d). The title compound was obtained as a light yellow solid from **8a** and 1-methyl-1,2,3,4-tetrahydroquinoxaline in yield of 85%. Mp 150 ℃. ^{1}H NMR (400 MHz, $CDCl_3$): δ 7.39 (d, J = 5.4 Hz, 1H), 7.20 (d, J = 8.5 Hz, 1H), 6.87 (dd, J = 8.5, 2.3 Hz, 1H), 6.82 (m, 1H), 6.70 (d, J = 7.7 Hz, 1H), 6.52 (d, J = 5.4 Hz, 1H), 6.41 (t, J = 7.4 Hz, 1H), 6.20 (d, J = 7.8 Hz, 1H), 6.11 (s, 1H), 3.99 (t, J = 5.2 Hz, 2H), 3.46 (t, J = 5.3 Hz, 2H), 2.75 (s, 3H). ^{13}C NMR (101 MHz, $CDCl_3$): δ 160.54, 152.16, 148.17, 139.39, 132.76, 130.35, 127.95, 126.60, 124.86, 123.45, 123.10, 122.78, 121.25, 119.15, 117.10, 114.95, 110.90, 50.80, 41.02, 37.67. IR (KBr) cm^{-1}: 1620.14, 1473.85, 1440.86, 1407.89, 1383.91, 1330.57, 1245.31, 739.88. HRMS (ESI$^+$) m/z calcd for $C_{20}H_{16}Cl_2N_2NaO_2S^+$ (M + Na)$^+$ 441.0202, found 441.0218.

(3-(3-Chlorophenoxy)thiophen-2-yl)(4-methyl-3,4-dihydroquinoxalin-1(2H)-yl)methanone (9e). The title compound was obtained as a light yellow solid from **8b** and 1-methyl-1,2,3,4-tetrahydroquinoxaline in yield of 87%. Mp 95 ℃. ^{1}H NMR (300 MHz, $CDCl_3$): δ 7.38 (d, J = 5.4 Hz, 1H), 7.09 (m, 1H), 6.95 (m, 2H), 6.71 (d, J = 7.7 Hz, 1H), 6.49 (m, 5H), 3.94 (m, 2H), 3.21 (t, J = 5.4 Hz, 2H), 2.81 (s, 3H). ^{13}C NMR (126 MHz, $CDCl_3$): δ 160.71, 157.47, 149.22, 139.51, 134.66, 130.04, 127.99, 126.40, 125.31, 123.04, 122.83, 122.27, 120.10, 117.21, 115.36, 114.48, 111.05, 50.82, 41.00, 37.97. IR (KBr) cm^{-1}: 1628.86, 1592.23, 1508.74, 1476.52, 1437.18, 1428.80, 1399.74, 1329.38, 1231.59, 740.93. HRMS (ESI$^+$) m/z calcd for $C_{20}H_{17}ClN_2NaO_2S^+$ (M + Na)$^+$ 407.0591, found 407.0608.

(3-(4-Bromo-2,5-dichlorophenoxy)thiophen-2-yl)(4-cyclopropyl-3,4-dihydroquinoxalin-1(2H)-yl)methanone (9f). The title compound was obtained as a light yellow solid from **8d** and 1-cyclopropyl-1,2,3,4-tetrahydroquinoxaline in yield of 73%. Mp 189 ℃. ^{1}H NMR (400 MHz, $CDCl_3$): δ 7.55 (s, 1H), 7.38 (d, J = 5.4 Hz, 1H), 6.88 (td, J = 7.8, 7.2, 1.3 Hz, 1H), 6.83 (d, J = 7.4 Hz, 1H), 6.72 (d, J = 7.7 Hz, 1H), 6.48 (m, 1H), 6.43 (d, J = 5.4 Hz, 1H), 6.17 (s, 1H), 3.93 (t, J = 5.3 Hz, 2H), 3.47 (t, J = 5.4 Hz, 2H), 2.29 (tt, J = 6.7, 3.7 Hz, 1H), 0.74 (m, 2H), 0.41 (m, 2H). ^{13}C NMR (126 MHz, $CDCl_3$): δ 160.35, 151.46, 148.44, 139.57, 133.52, 133.10, 128.19, 126.22, 125.32, 123.00, 122.60, 122.44, 118.80, 118.63, 115.73, 115.65, 112.98, 48.90, 41.06, 31.20, 7.95. IR (KBr) cm^{-1}: 1638.19, 1591.10, 1504.46, 1463.11, 1448.53, 1420.42, 1397.11, 1359.82, 1345.45, 1325.54, 1266.16, 1074.46. HRMS (ESI$^+$) m/z calcd for $C_{22}H_{17}BrCl_2N_2NaO_2S^+$ (M + Na)$^+$ 544.9463, found 544.9473.

(4-Cyclopropyl-3,4-dihydroquinoxalin-1(2H)-yl)(3-(4-(trifluoromethyl)phenoxy)thiophen-2-yl)methanone (9g). Mp 81 ℃. The title compound was obtained as a light yellow solid from **8e** and 1-cyclopropyl-1,2,3,4-tetrahydroquinoxaline in yield of 89%. ^{1}H NMR (300 MHz, $CDCl_3$): δ 7.42 (d, J = 8.6 Hz, 2H), 7.38 (d, J = 5.4 Hz, 1H), 6.96 (m, 2H), 6.74 (d, J = 7.9 Hz, 1H), 6.61 (d, J = 8.5 Hz, 2H), 6.48 (m, 2H), 3.89 (t, J = 5.5 Hz, 2H), 3.24 (t, J = 5.5 Hz, 2H), 2.27 (tt, J = 6.8, 3.7 Hz, 1H), 0.74 (td, J = 6.8, 4.9 Hz, 2H), 0.38 (m, 2H). ^{13}C NMR (126 MHz, $CDCl_3$): δ 160.61, 159.35, 149.24, 139.71, 128.08, 126.82 (q, J = 11.6 Hz), 125.92, 125.70, 125.16 (q, J = 32.8 Hz), 124.07 (q, J = 272.0 Hz), 122.80, 122.40, 119.86, 116.85, 116.20, 113.07, 48.79, 41.38, 31.21, 7.89. IR (KBr) cm^{-1}: 1630.29, 1608.25, 1509.20, 1436.46, 1324.56, 1235.52, 1165.64, 1123.83, 1066.86, 740.57. HRMS (ESI$^+$) m/z calcd for $C_{23}H_{19}F_3N_2NaO_2S^+$ (M + Na)$^+$ 467.1012, found 467.1010.

(4-Methyl-3,4-dihydroquinoxalin-1(2H)-yl)(3-(4-(trifluoromethyl)phenoxy)thiophen-2-yl)methanone (9h). The title compound was obtained as a light yellow solid from **8e** and 1-methyl-1,2,3,4-tetrahydroquinoxaline in yield of 92%. Mp 88 ℃. ^{1}H NMR (300 MHz, $CDCl_3$): δ 7.42 (m, 3H), 6.92 (t, J = 7.7 Hz, 1H), 6.70 (d, J = 7.9 Hz, 1H), 6.64 (d, J = 8.4 Hz, 2H), 6.55 (d, J = 5.3 Hz, 1H), 6.43 (d, J = 7.8 Hz, 2H), 3.92 (t, J = 5.3 Hz, 2H), 3.16 (t, J = 5.3 Hz, 2H), 2.78 (d, J = 1.0 Hz, 3H). ^{13}C NMR (126 MHz, $CDCl_3$): δ 160.50, 159.42, 148.70, 139.45, 128.17, 126.75 (q, J = 3.5 Hz), 126.42, 125.16, 124.85 (q, J = 32.8 Hz), 124.09 (q, J = 270.6 Hz), 122.90, 122.86, 120.32, 116.32, 115.44, 111.01, 50.72, 40.96, 37.86. IR (KBr) cm^{-1}: 1636.80, 1512.29, 1439.06, 1398.82, 1246.62, 1162.11, 1110.22, 1069.60. HRMS (ESI$^+$) m/z calcd for $C_{21}H_{17}F_3N_2NaO_2S^+$ (M + Na)$^+$ 441.0855, found 441.0859.

(3-(2-Bromo-4-(trifluoromethyl)phenoxy)thiophen-2-yl)(4-cyclopropyl-3,4-dihydroquinoxalin-1(2H)-yl)methanone (9i). The title compound was obtained as a light yellow solid from **8f** and 1-cyclopropyl-1,2,3,4-tetrahydroquinoxaline in yield of 83%. Mp 121 ℃. ^{1}H NMR (300 MHz, $CDCl_3$): δ 7.74 (s, 1H), 7.39 (d, J = 5.4 Hz, 1H), 7.23 (d, J =

8.6 Hz, 1H), 6.82 (d, *J* = 6.0 Hz, 2H), 6.74 (d, *J* = 7.9 Hz, 1H), 6.46 (m, 2H), 6.23 (d, *J* = 8.4 Hz, 1H), 3.93 (t, *J* = 5.3 Hz, 2H), 3.45 (t, *J* = 5.4 Hz, 2H), 2.29 (tt, *J* = 7.1, 3.9 Hz, 1H), 0.71 (dt, *J* = 6.7, 3.3 Hz, 2H), 0.42 (m, 2H). ^{13}C NMR (101 MHz, $CDCl_3$): δ 160.38, 155.73, 148.41, 139.55, 130.38 (q, *J* = 3.5 Hz), 128.03, 126.20 (q, *J* = 33.5 Hz), 126.01, 125.49 (q, *J* = 3.5 Hz), 125.26, 123.14 (q, *J* = 273.4 Hz), 122.98, 122.91, 119.17, 116.80, 115.83, 113.05, 112.24, 48.88, 41.19, 31.24, 7.89. IR (KBr) cm^{-1}: 1631.11, 1500.08, 1438.49, 1403.72, 1321.20, 1266.48, 1254.38, 1125.66, 1079.08, 737.60. HRMS (ESI$^+$) m/z calcd for $C_{23}H_{18}BrF_3N_2NaO_2S^+$ (M + Na)$^+$ 545.0117, found 545.0134

(3-(2-Bromo-4-(trifluoromethyl)phenoxy)thiophen-2-yl)(4-methyl-3,4-dihydroquinoxalin-1(2H)-yl)methanone (9j). The title compound was obtained as a light yellow solid from **8f** and 1-methyl-1,2,3,4-tetrahydroquinoxaline in yield of 76%. Mp 139 ℃. ^{1}H NMR (300 MHz, $CDCl_3$): δ 7.73 (d, *J* = 1.7 Hz, 1H), 7.42 (d, *J* = 5.4 Hz, 1H), 7.20 (m, 1H), 6.76 (m, 1H), 6.68 (d, *J* = 7.7 Hz, 1H), 6.55 (d, *J* = 5.4 Hz, 1H), 6.37 (m, 1H), 6.27 (d, *J* = 8.7 Hz, 1H), 6.18 (d, *J* = 8.1 Hz, 1H), 3.97 (t, *J* = 5.3 Hz, 2H), 3.43 (m, 2H), 2.77 (s, 3H). ^{13}C NMR (126 MHz, $CDCl_3$): δ 160.29, 155.61, 147.74, 139.36, 130.18 (q, *J* = 3.5 Hz), 128.03, 126.51, 125.77 (q, *J* = 33.4 Hz), 125.49 (q, *J* = 3.6 Hz), 124.82, 123.58, 123.19 (q, *J* = 341.8 Hz), 123.01, 119.60, 115.96, 115.06, 111.62, 110.91, 50.78, 40.99, 37.71. IR (KBr) cm^{-1}: 1642.62, 1605.25, 1438.73, 1400.55, 1324.18, 1266.31, 1252.39, 1114.12, 1078.46, 736.36. HRMS (ESI$^+$) m/z calcd for $C_{21}H_{16}BrF_3N_2NaO_2S^+$ (M + Na)$^+$ 518.9960, found 518.9975.

(3-(2-Chloro-4-(trifluoromethyl)phenoxy)thiophen-2-yl)(4-methyl-3,4-dihydroquinoxalin-1(2H)-yl)methanone (9k). The title compound was obtained as a light yellow solid from **8g** and 1-methyl-1,2,3,4-tetrahydroquinoxaline in yield of 80%. Mp 122 ℃. ^{1}H NMR (400 MHz, $CDCl_3$): δ 7.56 (d, *J* = 1.8 Hz, 1H), 7.42 (d, *J* = 5.4 Hz, 1H), 7.18 (m, 1H), 6.76 (m, 1H), 6.68 (d, *J* = 7.7 Hz, 1H), 6.56 (d, *J* = 5.4 Hz, 1H), 6.34 (m, 2H), 6.21 (d, *J* = 8.2 Hz, 1H), 3.97 (t, *J* = 5.3 Hz, 2H), 3.39 (m, 2H), 2.77 (s, 3H). ^{13}C NMR (126 MHz, $CDCl_3$): δ 160.28, 154.62, 147.64, 139.35, 128.06, 127.20 (q, *J* = 3.5 Hz), 126.50, 125.44 (q, *J* = 33.6 Hz), 124.79, 124.60 (q, *J* = 3.7 Hz), 123.59, 123.32 (q, *J* = 272.4 Hz), 123.10, 122.97, 119.65, 116.07, 115.10, 110.92, 50.73, 41.02, 37.64. IR (KBr) cm^{-1}: 1629.33, 1513.20, 1437.84, 1401.78, 1329.88, 1271.31, 1173.83, 1113.73, 1084.66. HRMS (ESI$^+$) m/z calcd for $C_{21}H_{16}ClF_3N_2NaO_2S^+$ (M + Na)$^+$ 475.0465, found 475.0470.

(4-Methyl-3,4-dihydroquinoxalin-1(2H)-yl)(3-(2-methyl-4-(trifluoromethyl)phenoxy)thiophen-2-yl)methanone (10). To a solution of **9j** (100 mg, 0.2 mmol) in dry 1,4-dioxane was added $Zn(CH_3)_2$ (333 μl, 0.4 mmol) and $Pd(dppf)Cl_2{\cdot}CH_2Cl_2$ (16 mg, 0.02mmol) under the nitrogen atmosphere. The resulting mixture was refluxed for 2 hours. Then the reaction was cooled to 0 ℃, quenched with MeOH, stirred for 10 mins and filtered. The filtrate was evaporated under vacuum. The residue was purified by flash column chromatography to provide 70 mg (81%) of **10** as a light yellow solid in yield of 81%. Mp 89 ℃. ^{1}H NMR (300 MHz, $CDCl_3$): δ 7.38 (d, *J* = 5.4 Hz, 1H), 7.34 (m, 1H),

7.18 (m, 1H), 6.87 (m, 1H), 6.74 (d, J = 7.8 Hz, 1H), 6.47 (d, J = 5.4 Hz, 1H), 6.42 (m, 1H), 6.35 (m, 2H), 3.97 (m, 2H), 3.26 (m, 2H), 2.76 (s, 3H), 2.11 (s, 3H). ^{13}C NMR (101 MHz, $CDCl_3$): δ 160.70, 157.34, 149.25, 139.37, 127.96, 127.91, 127.62 (q, J = 3.4 Hz), 126.36, 125.30, 124.88 (q, J = 3.7 Hz), 124.74 (q, J = 32.6 Hz), 124.16 (q, J = 271.1 Hz), 122.82, 122.05, 119.93, 115.53, 115.05, 111.19, 50.82, 41.00, 37.76, 15.96. IR (KBr) cm^{-1}: 1629.24, 1511.73, 1437.57, 1334.24, 1159.73, 1158.70, 1128.97, 1107.01, 750.03. HRMS (ESI$^+$) m/z calcd for $C_{22}H_{20}F_3N_2O_2S^+$ (M + H)$^+$ 433.1192, found 433.1198.

Methyl 3-((2-methoxyethoxy)methoxy)thiophene-2-carboxylate (11). To a solution of **5** (10.0 g, 63 mmol) and DIPEA (21 mL, 126 mmol) in DCM (250 mL) was slowly added MEMCl (10.9 mL, 95 mmol) at 0 ℃. The resulting mixture was stirred at room temperature overnight and then quenched with MeOH (10 mL). The solvent was evaporated under vacuum, and the residue was purified by flash column chromatography (petroleum ether/ethyl acetate = 8:1) to provide 12.3 g (79%) of **11** as a colorless oil. ^{1}H NMR (400 MHz, $CDCl_3$): δ 7.40 (d, J = 5.5 Hz, 1H), 7.04 (d, J = 5.6 Hz, 1H), 5.34 (s, 2H), 3.89 (m, 2H), 3.84 (s, 3H), 3.72 (m, 2H), 3.36 (s, 3H).

3-((2-Methoxyethoxy)methoxy)thiophene-2-carboxylic acid (12). To a solution of **11** (12.3 g, 50 mmol) in MeOH (250 mL) and H_2O (80 mL) was slowly added NaOH (5.0 g, 125 mmol). The resulting mixture was stirred at 40-50 ℃ overnight. Then the reaction mixture was cooled to room temperature, evaporated under vacuum, diluted with water and acidified with HCl (4N). The solid was filtered and dried at 45 ℃ overnight to afford 10.9 g (94%) of **12** as a light yellow solid. ^{1}H NMR (400 MHz, $CDCl_3$): δ 7.51 (d, J = 5.5 Hz, 1H), 7.06 (d, J = 5.5 Hz, 1H), 5.38 (s, 2H), 3.89 (m, 2H), 3.56 (m, 2H), 3.37 (s, 3H).

4-Methoxybenzyl 3-((2-methoxyethoxy)methoxy)thiophene-2-carboxylate (13). To a solution of **12** (5.7 g, 25 mmol) in DMF (150 mL) was added K_2CO_3 (10.2 g, 74 mmol) and PMBCl (5.0 mL, 37 mmol). The resulting mixture was stirred at room temperature overnight and then diluted with water, extracted with ethyl acetate for three times. The organic layer was combined, dried over anhydrous magnesium sulfate, and evaporated under vacuum. The residue was purified by was purified by flash column chromatography (petroleum ether/ethyl acetate = 5:1) to provide 7.8 g (90%) of **13** as a colorless oil. ^{1}H NMR (400 MHz, $CDCl_3$): δ 7.37 (m, 3H), 7.02 (d, J = 5.5 Hz, 1H), 6.89 (d, J = 8.6 Hz, 2H), 5.32 (s, 2H), 5.23 (s, 2H), 3.85 (m, 2H), 3.80 (s, 3H), 3.52 (m, 2H), 3.35 (s, 3H).

4-Methoxybenzyl 3-hydroxythiophene-2-carboxylate (14). To a solution of **13** (7.8 g, 22.5mmol) in MeOH was slowly added Amberlyst 15 (11.7 g, CAS 9037-24-5). The resulting mixture was stirred at 40-50 ℃ overnight. Then the reaction mixture cooled to room temperature, filtered with celite and evaporated under vacuum. The residue was purified by flash column chromatography (petroleum ether/ethyl acetate = 15:1) to provide 5.4 g (92%) of **14** as a light yellow solid. ^{1}H NMR (300 MHz, $CDCl_3$): δ 9.59 (s,

1H), 7.38 (m, 3H), 6.91 (d, J = 8.5 Hz, 2H), 6.73 (d, J = 5.4 Hz, 1H), 5.28 (s, 2H), 3.82 (s, 3H).

4-Methoxybenzyl 3-(5-chloro-4-(methoxycarbonyl)-2-nitrophenoxy)thiophene-2-carboxylate (15). To a solution of **14** (1.4 g, 5.3 mmol) in dry DMF (25 mL) was added K_2CO_3 (1.09 g，7.9 mmol) and methyl 2-chloro-4-fluoro-5-nitrobenzoate (**36**, 1.225 g, 5.3 mmol, synthesis procedure available in part 6 of Supplementary Information). The reaction mixture was stirred at room temperature overnight then poured into water (50 mL) and extracted with ethyl acetate for three times. Organic layers were combined, washed with saturated brine for three times, dried over anhydrous magnesium sulfate and evaporated under vacuum. The residue was purified by flash column chromatography (petroleum ether/ethyl acetate = 15:1) to provide 1.9 g (77%) of **15** as a brown solid. ^{1}H NMR (300 MHz, $CDCl_3$): δ 8.43 (s, 1H), 7.65 (d, J = 5.4 Hz, 1H), 7.08 (d, J = 8.5 Hz, 2H), 6.95 (d, J = 5.4 Hz, 1H), 6.78 (m, 3H), 5.08 (s, 2H), 3.96 (s, 3H), 3.81 (s, 3H).

4-Methoxybenzyl 3-(2,5-dichloro-4-(methoxycarbonyl)phenoxy)thiophene-2-carboxylate (16). To a solution of **15** (1.9 g, 4.0 mmol) in THF (80 mL) and H_2O (40 mL) was added iron power (1.8 g, 32 mmol) and NH_4Cl (0.43 g, 8.0 mmol). The resulting mixture was stirred at 60 ℃ overnight. Then the reaction mixture was cooled to room temperature, filtered, evaporated in vacuum, diluted with water and extracted with ethyl acetate for three times. Organic layers were combined, washed with saturated brine for three times, dried over anhydrous magnesium sulfate and evaporated under vacuum to yield the crude product which was subjected to the next step without further purification.

To a solution of the crude product obtained above in MeCN (50 mL) was added $CuCl_2$ (0.80 g, 6.0 mmol) and ^{t}BuONO (0.95 mL, 8.0 mmol), The resulting mixture was stirred at room temperature for 0.5 h and then heated at 60 ℃ overnight. Then the reaction mixture was cooled to room temperature, evaporated in vacuum, diluted with water and extracted with ethyl acetate for three times. Organic layers were combined, washed with saturated brine for three times, dried over anhydrous magnesium sulfate and evaporated under vacuum. The residue was purified by flash column chromatography (petroleum ether/ethyl acetate = 15:1) to provide 1.17 g (63%) of **16** as a brown solid. ^{1}H NMR (400 MHz, $CDCl_3$): δ 7.90 (s, 1H), 7.58 (d, J = 5.4 Hz, 1H), 7.15 (d, J = 8.6 Hz, 2H), 6.85 (d, J = 5.4 Hz, 1H), 6.81 (d, J = 8.7 Hz, 2H), 6.70 (s, 1H), 5.13 (s, 2H), 3.93 (s, 3H), 3.80 (s, 3H).

3-(2,5-Dichloro-4-(methoxycarbonyl)phenoxy)thiophene-2-carboxylic acid (17). To a solution of **16** (1.16 g, 25 mmol) in DCM (50 mL) was added CF_3COOH (1.5 mL). The resulting mixture was stirred at room temperature for 5 hours. The solvent was evaporated under vacuum, diluted with water, filtered and the solid dried at 45 ℃

overnight to afford **17** in quantitative yield as light yellow solid. ^{1}H NMR (400 MHz, $CDCl_3$): δ 8.03 (s, 1H), 7.61 (d, J = 5.4 Hz, 1H), 6.92 (s, 1H), 6.78 (d, J = 5.4 Hz, 1H), 3.93 (s, 3H).

Methyl 2,5-dichloro-4-((2-(4-cyclopropyl-1,2,3,4-tetrahydroquinoxaline-1-carbonyl)thiophen-3-yl)oxy)benzoate (18). To a solution of **17** (0.70 g, 2.0 mmol) in DCM was added DMF (1 drops) and $(COCl)_2$ (520 μl, 6.0 mmol). The resulting mixture was refluxed for 3 hours. After the reaction was cooled to room temperature, the solvent and $(COCl)_2$ was removed under vacuum. Then the residue was dissolved in DCM before adding DIPEA (0.99 mL, 6.0 mmol) and 1-cyclopropyl-1,2,3,4-tetrahydroquinoxaline (0.35 g, 2.0 mmol). The resulting mixture was stirred at room temperature overnight, then diluted with water, extracted with DCM. The organic layer was combined, washed with saturated brine for three times, dried over anhydrous magnesium sulfate, and evaporated under vacuum. The residue was purified by flash column chromatography (petroleum ether/ethyl acetate = 3:1) to provide 0.78 g (78%) of **16** as a brown solid. ^{1}H NMR (400 MHz, $CDCl_3$): δ 7.87 (s, 1H), 7.42 (d, J = 5.4 Hz, 1H), 6.84 (m, 1H), 6.76 (d, J = 7.8 Hz, 1H), 6.70 (d, J = 7.9 Hz, 1H), 6.51 (d, J = 5.4 Hz, 1H), 6.46 (m, 1H), 6.17 (s, 1H), 3.92 (m, 5H), 3.47 (t, J = 5.4 Hz, 2H), 2.27 (m, 1H), 0.72 (m, 2H), 0.44 (m, 2H).

2,5-Dichloro-4-((2-(4-cyclopropyl-1,2,3,4-tetrahydroquinoxaline-1-carbonyl)thiophen-3-yl)oxy)benzoic acid (19). To a solution of **18** (0.78 g, 1.55 mmol) in 1,4-dioxane (30 mL) and H_2O (10 mL) was added NaOH (124 mg, 3.1 mmol). The resulting mixture was stirred at room temperature for 3h. The organic layer was evaporated under vacuum, diluted with water and acidified with HCl (4N). The solid was filtered and dried at 45 ℃ overnight to afford 0.70 g (92%) of **19** as a brown solid. ^{1}H NMR (400 MHz, $CDCl_3$): δ 7.95 (s, 1H), 7.41 (d, J = 5.4 Hz, 1H), 6.84 (m, 1H), 6.77 (d, J = 8.0 Hz, 1H), 6.70 (d, J = 7.8 Hz, 1H), 6.50 (d, J = 5.4 Hz, 1H), 6.46 (m, 1H), 6.18 (s, 1H), 3.93 (t, J = 4.9 Hz, 2H), 3.47 (t, J = 5.3 Hz, 2H), 2.27 (tt, J = 6.7, 3.7 Hz, 1H), 0.66 (m, 2H), 0.41 (m, 2H).

2-(2,5-Dichloro-4-((2-(4-cyclopropyl-1,2,3,4-tetrahydroquinoxaline-1-carbonyl)thiophen-3-yl)oxy)-N-methylbenzamido)-N,N,N-trimethylethanaminium 2,2,2-trifluoroacetate (20). To a solution of **19a** (50 mg, 0.10 mmol) in DCM (10 mL) was added N,N,N-trimethyl-2-(methylamino)ethanaminium chloride hydrochloride (**39a**, 39 mg, 0.20 mmol, synthesis procedure available in part 6 of Supplementary Information), DIPEA (63 μl, 0.40 mmol) and HATU (74 mg, 0.20 mmol). The resulting mixture was stirred at room temperature overnight. Then the solvent was evaporated under vacuum. The residue was purified by preparative HPLC using 30-90% $MeCN/H_2O$ (containing 0.1% TFA) as the mobile phase to afford 64 mg (91%) of **20** as the light yellow solid. Mp 167 ℃. ^{1}H NMR (400 MHz, $CDCl_3$): δ 7.45 (d, J = 5.4 Hz, 1H), 7.26 (s, 1H), 6.85 (t, J = 7.5 Hz, 1H), 6.80 (d, J = 8.0 Hz, 1H), 6.72 (d, J = 7.7 Hz, 1H), 6.53 (d, J = 5.4 Hz, 1H), 6.47 (t, J = 7.4 Hz, 1H), 6.18 (s, 1H), 3.99 (s, 2H), 3.66 (s, 2H), 3.48 (s, 2H), 3.25 (s, 9H),

3.16 (m, 2H), 2.98 (s, 3H), 2.28 (s, 1H), 0.73 (d, J = 5.2 Hz, 2H), 0.43 (s, 2H). ^{13}C NMR (126 MHz, $CDCl_3$): δ 168.28, 160.66, 160.04 (q, J = 25.2 Hz), 152.95, 148.14, 139.68, 129.65, 128.89, 128.81, 128.60, 126.48, 124.82, 123.15, 122.47, 119.06, 117.73, 117.68(q, J = 286.0 Hz), 115.85, 114.29, 113.00, 62.06, 53.64, 48.77, 46.53, 41.81, 36.69, 31.26, 8.52, 7.87. IR (KBr) cm^{-1}: 1690.56, 1624.68, 1596.38, 1501.07, 1473.45, 1435.90, 1402.93, 1302.38, 837.27. HRMS (ESI$^+$) m/z calcd for $C_{29}H_{33}Cl_2N_4O_3S^+$ (M)$^+$ 587.1645, found 587.1631.

(E)-Ethyl 3-(2,5-dichloro-4-((2-(4-cyclopropyl-1,2,3,4-tetrahydroquinoxaline-1-carbonyl)thiophen-3-yl)oxy)phenyl)acrylate (21). To a suspension of **9f** (1.0 g, 1.91 mmol) in MeCN (15 mL), ethyl acrylate (625 µL, 5.73 mmol), palladium diacetate (43 mg, 0.19 mmol), tris(2-methylphenyl)phosphine (116 mg, 0.38 mmol), triethylamine (680 µL, 5.0 mmol) and LiCl (80 mg, 1.91 mmol) were added. The resulting mixture was heated using microwave reactor at 140 ℃ for 1 h. After cooling to ambient temperature, the reaction mixture was filtered, and the filtrate was concentrated under vacuum. The residue was purified by flash column chromatography (petroleum ether/ethyl acetate = 8:1) to afford 0.79 g (76%) of **21** as a light yellow solid. ^{1}H NMR (400 MHz, $CDCl_3$): δ 7.89 (d, J = 16 Hz, 1H), 7.56 (s, 1H), 7.41 (d, J = 5.6 Hz, 1H), 6.82 (m, 1H), 6.75 (d, J = 8.0 Hz, 1H), 6.70 (d, J = 7.6 Hz, 1H), 6.51 (d, J = 5.6 Hz, 1H), 6.47 (m, 1H), 6.34 (d, J = 16 Hz, 1H), 6.12 (s, 1H), 4.28 (q, J = 7.2 Hz, 2H), 3.93 (t, J = 5.2 Hz, 2H), 3.47 (t, J = 5.2 Hz, 2H), 2.27 (m, 1H), 1.35 (t, J = 7.2 Hz, 3H), 0.71 (m, 2H), 0.41 (m, 2H).

Ethyl 3-(2,5-dichloro-4-((2-(4-cyclopropyl-1,2,3,4-tetrahydroquinoxaline-1-carbonyl)thiophen-3-yl)oxy)phenyl)propanoate (24)

To a mixture of **21** (613 mg, 1.13 mmol) and cuprous chloride (168 mg, 1.70 mmol) in MeOH (7 mL) and THF (7 mL), $NaBH_4$ (214 mg, 5.65 mmol) was added at 0 ℃, and the resulting mixture was stirred at 0-5 ℃ for 2 h. Then the reaction was quenched by the addition of water. The resulting solid was filtered off, and the filtrate was concentrated under reduced pressure. The residue was diluted with water, extracted with ethyl acetate for three times. The organic layers were combined, dried over anhydrous magnesium sulfate and evaporated under reduced pressure. The residue was purified by flash column chromatography (petroleum ether/ethyl acetate = 8:1) to give 576 mg (92%) of **24** as a light yellow solid. ^{1}H NMR (300 MHz, $CDCl_3$): δ 7.35 (d, J = 5.4 Hz, 1H), 7.19 (s, 1H), 6.86 (m, 1H), 6.79 (d, J = 7.2 Hz, 1H), 6.74 (d, J = 7.8 Hz, 1H), 6.48 (m, 1H), 6.42 (d, J = 5.4 Hz, 1H), 6.07 (s, 1H), 4.15 (q, J = 7.2 Hz, 2H), 3.93 (t, J = 5.1 Hz, 2H), 3.48 (t, J = 5.1 Hz, 2H), 2.94 (t, J = 7.5 Hz, 2H), 2.58 (t, J = 7.5 Hz, 2H), 2.28 (m, 1H), 1.27 (t, J = 7.2 Hz, 3H), 0.70 (m, 2H), 0.39 (m, 2H).

(E)-Isobutyl 4-(2,5-dichloro-4-((2-(4-cyclopropyl-1,2,3,4-tetrahydroquinoxaline-1-carbonyl)thiophen-3-yl)oxy)phenyl)but-3-enoate (28). To a suspension of **9f** (0.50 g, 0.95 mmol) in MeCN (10 mL), isobutyl vinylacetate (460 μL, 2.9 mmol), palladium diacetate (21 mg, 0.094 mmol), tris(2-methylphenyl)phosphine (58 mg, 0.19 mmol), triethylamine (340 μL, 2.5 mmol) and LiCl (40 mg, 0.95 mmol) were added. The resulting mixture was heated using microwave reactor at 140 ℃ for 1 h. After cooling to ambient temperature, the reaction mixture was filtered, and the filtrate was concentrated under vacuum. The residue was purified by flash column chromatography (petroleum ether/ethyl acetate = 8:1) to afford 0.42 g (76%) of **28** as a light yellow solid. ^{1}H NMR (400 MHz, $CDCl_3$): δ 7.48 (s, 1H), 7.36 (d, J = 5.5 Hz, 1H), 6.87 (d, J = 8.3 Hz, 1H), 6.82 (m, 1H), 6.74 (d, J = 7.1 Hz, 1H), 6.70 (d, J = 16.0 Hz, 1H), 6.46 (d, J = 5.4 Hz, 1H), 6.44 (d, J = 5.4 Hz, 1H), 6.23 (dt, J = 15.7, 7.1 Hz, 1H), 6.08 (s, 1H), 3.94 (m, 4H), 3.47 (t, J = 5.5 Hz, 2H), 3.30 (dd, J = 7.1, 1.2 Hz, 2H), 2.28 (tt, J = 6.7, 3.5 Hz, 1H), 1.97 (tdd, J = 11.2, 7.4, 5.1 Hz, 1H), 0.96 (d, J = 6.7 Hz, 6H), 0.70 (ddd, J = 6.9, 4.2, 2.6 Hz, 2H), 0.41 (q, J = 4.6, 3.8 Hz, 2H).

Isobutyl 4-(2,5-dichloro-4-((2-(4-cyclopropyl-1,2,3,4-tetrahydroquinoxaline-1-carbonyl)thiophen-3-yl)oxy)phenyl)butanoate (31). 10% Pd/C (12 mg) was added to a solution of **28** (118 mg, 0.20 mmol) in MeOH (8 mL), and the resulting mixture was stirred under an atmosphere of hydrogen (1 atm) at room temperature for 2 h. The reaction mixture was filtered over celite and the filtrate was evaporated under vacuum. The residue was purified by flash column chromatography (petroleum ether/ethyl acetate = 8:1) to afford 104 mg (88%) of **31** as light yellow oil. ^{1}H NMR (400 MHz, $CDCl_3$): δ 7.36 (d, J = 5.4 Hz, 1H), 7.15 (s, 1H), 6.87 (t, J = 7.7 Hz, 1H), 6.80 (d, J = 8.1 Hz, 1H), 6.75 (d, J = 7.5 Hz, 1H), 6.48 (t, J = 7.5 Hz, 1H), 6.44 (d, J = 5.4 Hz, 1H), 6.07 (s, 1H), 3.94 (t, J = 5.0 Hz, 2H), 3.89 (d, J = 6.7 Hz, 2H), 3.47 (t, J = 5.4 Hz, 2H), 2.66 (m, 2H), 2.36 (t, J = 7.4 Hz, 2H), 2.28 (tt, J = 6.9, 3.6 Hz, 1H), 1.92 (m, 3H), 0.95 (d, J = 6.7 Hz, 6H), 0.70 (td, J = 6.7, 4.7 Hz, 2H), 0.39 (m, 2H).

General procedure for preparation of compound 22,25,29,32. To a solution of **21**, **24**, **28**, **31** (0.25 mmol) in 1,4-dioxane (7 mL) and H_2O (7 mL) was added NaOH (20 mg, 0.50 mmol). The resulting mixture was stirred at room temperature overnight. Then the reaction mixture was evaporated under vacuum, diluted with water and acidified with 4N HCl. The solid formed was filtered and dried at 45 ℃ overnight to afford the desired compound **22**, **25**, **29**, **32**.

(E)-3-(2,5-Dichloro-4-((2-(4-cyclopropyl-1,2,3,4-tetrahydroquinoxaline-1-carbonyl)thiophen-3-yl)oxy)phenyl)acrylic acid (22). The title compound was obtained as a white solid from **21** in yield of 93%. ^{1}H NMR (400 MHz, $CDCl_3$): δ 8.00 (d, J = 15.9 Hz, 1H), 7.59 (s, 1H), 7.42 (d, J = 5.4 Hz, 1H), 6.84 (t, J = 7.3 Hz, 1H), 6.77 (d, J = 8.2 Hz, 1H),

6.71 (d, J = 7.5 Hz, 1H), 6.50 (m, 2H), 6.36 (d, J = 15.9 Hz, 1H), 6.16 (s, 1H), 3.92 (t, J = 4.8 Hz, 2H), 3.48 (t, J = 5.3 Hz, 2H), 2.28 (m, 1H), 0.70 (m, 2H), 0.43 (m, 2H).

3-(2,5-Dichloro-4-((2-(4-cyclopropyl-1,2,3,4-tetrahydroquinoxaline-1-carbonyl)thiophen-3-yl)oxy)phenyl)propanoic acid (25). The title compound was obtained as a white solid from **24** in yield of 95%. ^{1}H NMR (300 MHz, $CDCl_3$): δ 7.35 (d, J = 5.4 Hz, 1H), 7.20 (s, 1H), 6.87 (m, 1H), 6.79 (d, J = 7.8, 1H), 6.74 (d, J = 7.8 Hz, 1H), 6.48 (m, 1H), 6.43 (d, J = 5.4 Hz, 1H), 6.09 (s, 1H), 3.93 (t, J = 5.1 Hz, 2H), 3.46 (t, J = 5.1 Hz, 2H), 2.94 (t, J = 7.5 Hz, 2H), 2.64 (t, J = 7.5 Hz, 2H), 2.28 (m, 1H), 0.67 (m, 2H), 0.39 (m, 2H).

(E)-4-(2,5-Dichloro-4-((2-(4-cyclopropyl-1,2,3,4-tetrahydroquinoxaline-1-carbonyl)thiophen-3-yl)oxy)phenyl)but-3-enoic acid (29). The title compound was obtained as a white solid from **28** in yield of 88%. ^{1}H NMR (400 MHz, $CDCl_3$): δ 7.48 (s, 1H), 7.37 (d, J = 5.5 Hz, 1H), 6.87 (d, J = 7.4 Hz, 1H), 6.84 (s, 1H), 6.79 (m, 1H), 6.72 (d, J = 15.8 Hz, 1H), 6.50 (m, 1H), 6.43 (d, J = 5.3 Hz, 1H), 6.21 (dt, J = 14.9, 7.1 Hz, 1H), 6.09 (s, 1H), 3.94 (m, 2H), 3.46 (m, 2H), 3.34 (d, J = 6.9 Hz, 2H), 2.28 (tt, J = 7.1, 3.8 Hz, 1H), 0.71 (d, J = 5.6 Hz, 2H), 0.41 (s, 2H).

4-(2,5-Dichloro-4-((2-(4-cyclopropyl-1,2,3,4-tetrahydroquinoxaline-1-carbonyl)thiophen-3-yl)oxy)phenyl)butanoic acid (32). The title compound was obtained as a white solid from **31** in yield of 92%. ^{1}H NMR (400 MHz, $CDCl_3$): δ 7.36 (d, J = 5.4 Hz, 1H), 7.15 (s, 1H), 6.88 (m, 1H), 6.81 (d, J = 8.0 Hz, 1H), 6.75 (d, J = 7.8 Hz, 1H), 6.50 (m, 1H), 6.44 (d, J = 5.4 Hz, 1H), 6.09 (s, 1H), 3.94 (t, J = 5.2 Hz, 2H), 3.47 (t, J = 5.4 Hz, 2H), 2.78 (t, J = 7.6 Hz,, 2H), 2.41 (t, J = 7.4 Hz, 2H), 2.28 (tt, J = 6.7, 3.8 Hz, 1H), 1.92 (p, J = 7.5 Hz, 2H), 0.70 (td, J = 6.7, 4.7 Hz, 2H), 0.36 (m, 2H).

General procedure for preparation of compound 23, 26a-h, 30, 33. To a solution of **22, 25, 29, 32** (0.10 mmol) in DCM (10 mL) was added amine derivatives (0.20 mmol), DIPEA (63 μl, 0.40 mmol) and HATU (74 mg, 0.20 mmol). The resulting mixture was stirred at room temperature overnight. Then the solvent was evaporated under vacuum. The residue was purified by preparative HPLC using 30-90% MeCN/H_2O (containing 0.1% TFA) as the mobile phase to afford **23, 26a-h, 30, 33.**

(E)-2-(3-(2,5-Dichloro-4-((2-(4-cyclopropyl-1,2,3,4-tetrahydroquinoxaline-1-carbonyl)thiophen-3-yl)oxy)phenyl)-N-methylacrylamido)-N,N,N-trimethylethanaminium 2,2,2-trifluoroacetate (23). The title compound was obtained as a light yellow solid from **22** and amine derivative **39a** (synthesis procedure available in part 6 of Supplementary Information) in yield of 89%. Mp 123 ℃. ^{1}H NMR (400 MHz, $CDCl_3$): δ 7.83 (d, J = 14.8 Hz, 1H), 7.60 (s, 1H), 7.41 (d, J = 5.0 Hz, 1H), 6.82 (m, 3H), 6.70 (d, J = 6.8 Hz, 1H), 6.50 (d, J = 4.9 Hz, 1H), 6.45 (t, J = 7.1 Hz, 1H), 6.17 (s, 1H), 3.91 (m, 4H), 3.59 (m, 2H), 3.45 (m, 2H), 3.26 (s, 3H), 3.22 (s, 9H), 2.28 (s, 1H), 0.71 (d, J = 5.6 Hz, 2H),

0.41 (s, 2H). ^{13}C NMR (126 MHz, $CDCl_3$): δ 167.15, 160.42, 159.86 (q, J = 30.2 Hz), 153.04, 148.24, 139.57, 138.25, 138.22, 133.56, 128.41, 128.34, 126.39, 124.89, 123.87 (q, J = 226.8 Hz), 123.07, 122.58, 122.28, 119.07, 118.54, 118.08, 115.75, 113.02, 62.57, 53.55, 48.79, 42.70, 41.26, 35.86, 31.21, 7.91. IR (KBr) cm^{-1}: 1647.49, 1471.90, 1435.64, 1400.62, 843.12. HRMS (ESI$^+$) m/z calcd for $C_{31}H_{35}Cl_2N_4O_3S^+$ (M)$^+$ 613.1801, found 613.1793.

2-(3-(2,5-Dichloro-4-((2-(4-cyclopropyl-1,2,3,4-tetrahydroquinoxaline-1-carbonyl)thiophen-3-yl)oxy)phenyl)-N-methylpropanamido)-N,N,N-trimethylethanaminium 2,2,2-trifluoroacetate (26a). The title compound was obtained as a light yellow solid from **25** and amine derivative **39a** (synthesis procedure available in part 6 of Supplementary Information) in yield of 79%. Mp 120 ℃. ^{1}H NMR (400 MHz, $CDCl_3$): δ 7.37 (d, J = 5.4 Hz, 1H), 7.22 (s, 1H), 6.89 (m, 2H), 6.76 (d, J = 7.6 Hz, 1H), 6.46 (m, 2H), 6.16 (s, 1H), 3.91 (m, 2H), 3.79 (m, 2H), 3.44 (m, 4H), 3.14 (s, 9H), 3.04 (s, 3H), 2.89 (m, 2H), 2.58 (m, 2H), 2.29 (s, 1H), 0.70 (d, J = 6.1 Hz, 2H), 0.39 (s, 2H). ^{13}C NMR (126 MHz, $CDCl_3$): δ 173.16, 160.90, 159.38 (q, J = 36.5 Hz), 150.75, 149.62, 139.71, 134.38, 132.23, 131.26, 128.31, 126.31, 125.13, 123.16, 121.92, 121.10, 118.97, 118.80, 115.83,115.26 (q, J = 287.3 Hz), 113.11, 62.55, 53.39, 48.81, 42.14, 41.72, 35.59, 32.94, 31.17, 28.07, 7.91. IR (KBr) cm^{-1}: 1633.41, 1504.09, 1477.25, 1435.50, 1402.59, 1373.38, 1206.15, 1143.39, 842.55, 558.21. HRMS (ESI$^+$) m/z calcd for $C_{31}H_{37}Cl_2N_4O_3S^+$ (M)$^+$ 615.1958, found 615.1938.

2-(3-(2,5-Dichloro-4-((2-(4-cyclopropyl-1,2,3,4-tetrahydroquinoxaline-1-carbonyl)thiophen-3-yl)oxy)phenyl)-N-ethylpropanamido)-N,N,N-trimethylethanaminium 2,2,2-trifluoroacetate (26b). The title compound was obtained as light yellow oil from **25** and amine derivative **39b** (synthesis procedure available in part 6 of Supplementary Information) in yield of 63%.^{1}H NMR (400 MHz, $CDCl_3$): δ 7.37 (d, J = 5.4 Hz, 1H), 7.21 (s, 1H), 6.91 (m, 2H), 6.79 (d, J = 7.7 Hz, 1H), 6.52 (m, 1H), 6.45 (d, J = 5.3 Hz, 1H), 6.19 (s, 1H), 3.93 (t, J = 5.1 Hz, 2H), 3.75 (m, 2H), 3.49 (m, 2H), 3.45 (m, 2H), 3.37 (d, J = 5.9 Hz, 2H), 3.20 (s, 9H), 2.94 (s, 2H), 2.59 (s, 2H), 2.30 (tt, J = 5.9, 3.4 Hz, 1H), 1.15 (t, J = 5.5 Hz, 3H), 0.71 (d, J = 5.5 Hz, 2H), 0.41 (s, 2H). ^{13}C NMR (126 MHz, $CDCl_3$): δ 172.73, 160.79, 158.31（q, J = 45.4 Hz）, 150.95, 149.55, 139.69, 134.07, 132.21, 131.31, 128.16, 126.21, 125.24, 123.19, 121.89, 121.40, 118.95, 118.77, 115.86, 114.80 (q, J = 213.6 Hz), 113.03, 62.88, 53.73, 48.85, 43.30, 41.62, 39.88, 32.34, 31.16, 28.42, 13.85, 7.89. IR (KBr) cm^{-1}: 2922.25, 1686.17, 1630.66, 1503.17, 1477.21, 1437.05, 1401.66, 1200.73, 1123.40, 1081.97, 844.12. HRMS (ESI$^+$) m/z calcd for $C_{32}H_{39}Cl_2N_4O_3S^+$ (M)$^+$ 629.2114, found 629.2098.

3-(3-(2,5-Dichloro-4-((2-(4-cyclopropyl-1,2,3,4-tetrahydroquinoxaline-1-carbonyl)thiophen-3-yl)oxy)phenyl)-N-methylpropanamido)-N,N,N-trimethylpropan-1-aminium 2,2,2-trifluoroacetate (26c). The title compound was obtained as a light yellow solid from **25** and amine derivative **39c** (synthesis procedure available in part 6 of

Supplementary Information) in yield of 79%. Mp 94 ℃. ^{1}H NMR (400 MHz, $CDCl_3$): δ 7.37 (d, J = 5.2 Hz, 1H), 7.24 (s, 1H), 6.89 (m, 2H), 6.77 (d, J = 7.4 Hz, 1H), 6.49 (d, J = 7.2 Hz, 1H), 6.44 (d, J = 5.2 Hz, 1H), 6.16 (s, 1H), 3.92 (m, 2H), 3.44 (m, 4H), 3.32 (m, 2H), 3.10 (s, 9H), 3.00 (m, 3H), 2.92 (m, 2H), 2.59 (m, 2H), 2.29 (m, 1H), 2.00 (m, 2H), 0.71 (d, J = 6.6 Hz, 2H), 0.40 (s, 2H). ^{13}C NMR (126 MHz, $CDCl_3$): δ 172.90, 160.80, 160.20（q, J = 37.8 Hz）, 150.66, 149.56, 139.68, 134.64, 132.23, 131.26, 128.18, 126.24, 125.16, 123.13, 121.92 (q, J = 263.3 Hz), 121.83, 121.21, 118.92, 118.71, 115.79, 113.07, 64.63, 53.20, 48.79, 44.97, 41.68, 35.57, 32.95, 31.15, 28.22, 21.22, 7.88. IR (KBr) cm^{-1}: 1631.14, 1503.77, 1477.27, 1436.74, 1402.15, 1187.38, 842.38. HRMS (ESI$^+$) m/z calcd for $C_{32}H_{39}Cl_2N_4O_3S^+$ (M)$^+$ 629.2114, found 629.2098.

4-(3-(2,5-Dichloro-4-((2-(4-cyclopropyl-1,2,3,4-tetrahydroquinoxaline-1-carbonyl)thiophen-3-yl)oxy)phenyl)propanoyl)-1,1-dimethylpiperazin-1-ium 2,2,2-trifluoroacetate (26d). The title compound was obtained as a light yellow solid from **25** and amine derivative **41** (synthesis procedure available in part 6 of Supplementary Information) in yield of 83%. Mp 131 ℃. ^{1}H NMR (400 MHz, DMSO-d_6): δ 7.78 (d, J = 5.4 Hz, 1H), 7.55 (s, 1H), 6.90 (m, 1H), 6.82 (d, J = 7.9 Hz, 1H), 6.71 (d, J = 7.7 Hz, 1H), 6.63 (d, J = 5.4 Hz, 1H), 6.46 (m, 1H), 6.11 (s, 1H), 3.82 (m, 6H), 3.45 (m, 2H), 3.38 (q, J = 6.2, 5.6 Hz, 4H), 3.17 (s, 6H), 2.84 (m, 2H), 2.64 (m, 2H), 2.27 (tt, J = 6.8, 3.7 Hz, 1H), 0.71 (dt, J = 6.5, 3.2 Hz, 2H), 0.31 (m, 2H). ^{13}C NMR (126 MHz, DMSO-d_6): δ 170.24, 160.31, 158.73 (q, J = 35.4 Hz), 150.49, 148.99, 139.78, 135.19, 132.07, 131.64, 129.85, 126.24, 125.31, 123.24, 121.67, 121.38, 119.41, 118.26, 116.50 (q, J = 292.8 Hz), 115.66, 113.11, 60.62, 60.50, 50.94, 48.86, 41.76, 39.25, 35.66, 32.21, 31.37, 27.77, 7.98. IR (KBr) cm^{-1}: 1654.09, 1629.59, 1475.12, 1437.30, 841.42, 558.16. HRMS (ESI$^+$) m/z calcd for $C_{31}H_{35}C_{l2}N_4O_3S^+$ (M)$^+$ 613.1801, found 613.1804.

1-(3-(2,5-Dichloro-4-((2-(4-cyclopropyl-1,2,3,4-tetrahydroquinoxaline-1-carbonyl)thiophen-3-yl)oxy)phenyl)propanoyl)-N,N,N-trimethylpiperidin-4-aminium 2,2,2-trifluoroacetate (26e). The title compound was obtained as a light yellow solid from **25** and amine derivative **44** (synthesis procedure available in part 6 of Supplementary Information) in yield of 73%. Mp 133 ℃. ^{1}H NMR (400 MHz, $CDCl_3$): δ 7.38 (m, 1H), 7.25 (s, 1H), 6.90 (m, 2H), 6.78 (m, 1H), 6.46 (m, 2H), 6.22 (s, 1H), 4.80 (s, 1H), 3.91 (s, 4H), 3.43 (s, 4H), 3.02 (m, 9H), 2.61 (s, 4H), 2.30 (s, 2H), 2.18 (m, 1H), 1.55 (m, 2H), 0.71 (m, 2H), 0.40 (m, 2H). ^{13}C NMR (126 MHz, $CDCl_3$): δ 170.73, 160.93, 160.66 (q, J = 34.0 Hz), 150.77, 149.75, 139.75, 134.48, 132.25, 131.41, 128.39, 126.40, 124.95, 123.25, 121.84, 121.02, 119.04, 118.70, 117.56 (q, J = 260.8 Hz), 115.86, 113.18, 72.06, 50.95, 48.76, 43.80, 41.89, 40.19, 32.48, 31.19, 28.44, 25.94, 25.37, 7.90. IR (KBr) cm^{-1}: 1632.14, 1475.88, 1436.68, 1208.86, 842.38. HRMS (ESI$^+$) m/z calcd for $C_{33}H_{39}Cl_2N_4O_3S^+$ (M)$^+$ 641.2114, found 641.2095.

(3,3'-((3-(2,5-Dichloro-4-((2-(4-cyclopropyl-1,2,3,4-tetrahydroquinoxaline-1-carbony

l)thiophen-3-yl)oxy)phenyl)propanoyl)azanediyl)bis(N,N,N-trimethylpropan-1-aminium)) di(2,2,2-trifluoroacetate) (26f). The title compound was obtained as a light yellow solid from **25** and amine derivative **47** (synthesis procedure available in part 6 of Supplementary Information) in yield of 65%. Mp 126 ℃. ^{1}H NMR (500 MHz, DMSO-d_6): δ 7.76 (d, J = 5.4 Hz, 1H), 7.51 (s, 1H), 6.88 (t, J = 8.3 Hz, 1H), 6.83 (d, J = 7.9 Hz, 1H), 6.72 (d, J = 7.7 Hz, 1H), 6.59 (d, J = 5.4 Hz, 1H), 6.45 (t, J = 8.1 Hz, 1H), 6.14 (s, 1H), 3.81 (s, 2H), 3.32 (m, 10H), 3.06 (s, 9H), 3.05 (s, 9H), 2.86 (m, 2H), 2.62 (m, 2H), 2.28 (tt, J = 6.7, 3.7 Hz, 1H), 1.91 (m, 4H), 0.70 (dt, J = 6.5, 3.2 Hz, 2H), 0.31 (m, 2H). ^{13}C NMR (126 MHz, DMSO-d_6): δ 171.39, 160.34, 158.62 (q, J = 47.8 Hz), 150.52, 149.16, 139.82, 135.49, 132.16, 131.67, 129.88, 126.23, 125.34, 123.28, 121.55, 121.44, 119.35, 119.35 (q, J = 226.5 Hz), 118.52, 115.71, 113.11, 63.65, 63.11, 52.81, 52.70, 48.86, 44.09, 42.17, 32.07, 31.37, 28.11, 22.37, 21.41, 7.98, 1.56. IR (KBr) cm^{-1}: 1631.93, 1477.97, 839.38, 558.26. HRMS (ESI^{+}) m/z calcd for ($C_{37}H_{51}Cl_2N_5O_3S^{2+}$)/2 $(M)^{2+}$/2 357.6539, found 357.6549.

(3,3'-((3-(2,5-Dichloro-4-((2-(4-cyclopropyl-1,2,3,4-tetrahydroquinoxaline-1-carbonyl)thiophen-3-yl)oxy)phenyl)propanoyl)azanediyl)bis(N-(2-ethoxy-2-oxoethyl)-N,N-dimethylpropan-1-aminium)) di(2,2,2-trifluoroacetate) (26g). The title compound was obtained as a light yellow solid from **25** and amine derivative **48** (synthesis procedure available in part 6 of Supplementary Information) in yield of 65%. ^{1}H NMR (400 MHz, $CDCl_3$): δ 7.36 (d, J = 5.2 Hz, 1H), 7.25 (s, 1H), 6.90 (m, 2H), 6.80 (m, 1H), 6.50 (m, 1H), 6.46 (d, J = 5.2 Hz, 1H), 6.21 (s, 1H), 4.28 (m, 8H), 3.93 (m, 2H), 3.65 (m, 4H), 3.38 (m, 18H), 2.93 (d, J = 7.6 Hz, 2H), 2.58 (d, J = 7.6 Hz, 2H), 2.30 (m, 1H), 2.12 (m, 4H), 1.29 (m, 6H), 0.72 (m, 2H), 0.42 (m, 2H).

2-(3-(2,5-Dichloro-4-((2-(4-cyclopropyl-1,2,3,4-tetrahydroquinoxaline-1-carbonyl)thiophen-3-yl)oxy)phenyl)-N-(3-ethoxy-3-oxopropyl)propanamido)-N,N,N-trimethylethanaminium 2,2,2-trifluoroacetate (26h). The title compound was obtained as a light yellow solid from **25** and amine derivative **52** (synthesis procedure available in part 6 of Supplementary Information) in yield of 72%. ^{1}H NMR (500 MHz, $CDCl_3$): δ 7.37 (d, J = 5.4 Hz, 1H), 7.21 (s, 1H), 6.89 (m, 2H), 6.78 (d, J = 7.8 Hz, 1H), 6.50 (d, J = 7.0 Hz, 1H), 6.47 (d, J = 5.4 Hz, 1H), 6.18 (s, 1H), 4.12 (q, J = 7.1 Hz, 2H), 3.93 (m, 2H), 3.78 (s, 2H), 3.64 (s, 2H), 3.48 (m, 4H), 3.20 (s, 9H), 2.92 (m, 2H), 2.69 (m, 2H), 2.58 (s, 2H), 2.00 (m, 1H), 1.23 (q, J = 7.2 Hz, 3H), 0.72 (d, J = 5.2 Hz, 2H), 0.41 (s, 2H).

(E)-2-(4-(2,5-Dichloro-4-((2-(4-cyclopropyl-1,2,3,4-tetrahydroquinoxaline-1-carbonyl)thiophen-3-yl)oxy)phenyl)-N-methylbut-3-enamido)-N,N,N-trimethylethanaminium 2,2,2-trifluoroacetate (30). The title compound was obtained as a light yellow solid from **29** and amine derivative **39a** (synthesis procedure available in part 6 of Supplementary Information) in yield of 82%. Mp 96 ℃. ^{1}H NMR (400 MHz, $CDCl_3$): δ

7.52 (s, 1H), 7.38 (d, J = 5.4 Hz, 1H), 6.88 (m, 2H), 6.76 (d, J = 7.3 Hz, 1H), 6.67 (d, J = 15.8 Hz, 1H), 6.49 (t, J = 6.8 Hz, 1H), 6.45 (d, J = 5.4 Hz, 1H), 6.22 (dt, J = 15.2, 6.9 Hz, 1H), 6.14 (s, 1H), 3.95 (m, 2H), 3.83 (m, 2H), 3.55 (m, 2H), 3.46 (t, J = 5.1 Hz, 2H), 3.35 (d, J = 5.8 Hz, 2H), 3.19 (s, 9H), 3.14 (s, 3H), 2.30 (m, 1H), 0.72 (d, J = 5.6 Hz, 2H), 0.42 (s, 2H). ^{13}C NMR (126 MHz, $CDCl_3$): δ 172.39, 160.78, 160.15 (q, J = 35.3 Hz), 151.26, 149.29, 139.68, 131.08, 130.99, 128.22, 128.01, 127.50, 126.30, 125.17, 125.14, 123.05, 122.40, 121.44, 120.08 (q, J = 281.0 Hz), 118.82, 118.57, 115.79, 113.11, 62.58, 53.58, 48.82, 42.31, 41.54, 37.38, 35.83, 31.17, 7.91. IR (KBr) cm^{-1}: 1687.98, 1632.67, 1503.15, 1473.15, 1435.39, 1401.38, 1200.69, 843.46. HRMS (ESI$^+$) m/z calcd for $C_{32}H_{37}Cl_2N_4O_3S^+$ (M)$^+$ 627.1958, found 627.1947.

2-(4-(2,5-Dichloro-4-((2-(4-cyclopropyl-1,2,3,4-tetrahydroquinoxaline-1-carbonyl)thiophen-3-yl)oxy)phenyl)-N-methylbutanamido)-N,N,N-trimethylethanaminium 2,2,2-trifluoroacetate (33). The title compound was obtained as a light yellow solid from **32** and amine derivative **39a** (synthesis procedure available in part 6 of Supplementary Information) in yield of 76%. Mp 97 ℃. ^{1}H NMR (400 MHz, $CDCl_3$): δ 7.37 (d, J = 5.4 Hz, 1H), 7.19 (s, 1H), 6.89 (m, 2H), 6.77 (d, J = 7.5 Hz, 1H), 6.49 (t, J = 7.3 Hz, 1H), 6.44 (d, J = 5.3 Hz, 1H), 6.14 (s, 1H), 3.93 (m, 2H), 3.81 (m, 2H), 3.51 (m, 2H), 3.45 (m, 2H), 3.18 (s, 9H), 3.07 (s, 3H), 2.66 (m, 2H), 2.39 (m, 2H), 2.30 (s, 1H), 1.88 (m, 2H), 0.71 (d, J = 5.6 Hz, 2H), 0.39 (s, 2H). ^{13}C NMR (126 MHz, $CDCl_3$): δ 173.97, 160.88, 160.12（q, J = 30.2 Hz）,150.41, 149.65, 139.68, 135.36, 132.21, 130.86, 128.14, 126.24, 125.24, 123.09, 121.95, 121.08, 120.08 (q, J = 250.7 Hz),118.83, 118.80, 115.78, 113.07, 62.67, 53.55, 48.83, 42.17, 41.63, 35.61, 32.48, 32.00, 31.13, 24.60, 7.89. IR (KBr) cm^{-1}: 1629.90, 1501.50, 1476.44, 1437.40, 1401.75, 842.69, 558.26. HRMS (ESI$^+$) m/z calcd for $C_{32}H_{39}Cl_2N_4O_3S^+$ (M)$^+$ 629.2114, found 629.2101.

General procedure for preparation of compound 27a–b. To a solution of **26g–h** (0.067 mmol) in 1,4-dioxane (2 mL) and H_2O (2 mL) was added LiOH (0.40 mmol). The resulting mixture was stirred at room temperature overnight. Then the reaction solution was concentrated under vacuum and the residue was purified by preparative HPLC using 30-90% MeCN/H_2O (containing 0.1% TFA) as the mobile phase to afford **27a–b**.

2,2'-((((3-(2,5-Dichloro-4-((2-(4-cyclopropyl-1,2,3,4-tetrahydroquinoxaline-1-carbonyl)thiophen-3-yl)oxy)phenyl)propanoyl)azanediyl)bis(propane-3,1-diyl))bis(dimethylammonionediyl))diacetate (27a). The title compound was obtained as a light yellow solid from **26g** in yield of 78%. Mp 55 ℃. ^{1}H NMR (500 MHz, Methanol-d_4): δ 7.55 (d, J = 5.4 Hz, 1H), 7.36 (s, 1H), 6.88 (m, 1H), 6.82 (d, J = 8.2 Hz, 1H), 6.65 (m, 1H), 6.41 (m, 2H), 5.97 (s, 1H), 3.84 (s, 2H), 3.57 (s, 2H), 3.52 (m, 4H), 3.36 (m, 10H), 3.21 (s, 6H), 3.20 (s, 6H), 2.91 (s, 2H), 2.25 (tt, J = 6.8, 3.8 Hz, 1H), 2.00 (m, 4H), 0.66 (m, 2H), 0.28 (m, 2H). ^{13}C NMR (126 MHz, Methanol-d_4): δ 172.88, 167.41, 167.38, 161.28, 150.36, 149.87, 139.95, 135.10, 132.13, 131.32, 128.65, 126.34, 125.24, 122.77, 121.87, 120.16, 118.84, 118.32, 115.34, 113.10, 72.33, 63.02, 61.80, 61.17, 50.34, 48.54, 48.47, 44.64,

42.75, 41.40, 30.77, 27.98, 27.92, 22.02, 21.08, 7.17. IR (KBr) cm^{-1}: 1629.81, 1475.93, 1436.22, 843.78, 558.61. HRMS (ESI^+) m/z calcd for $C_{39}H_{49}Cl_2N_5NaO_7S^+$ $(M + Na)^+$ 824.2622, found 824.2619.

3-(3-(2,5-Dichloro-4-((2-(4-cyclopropyl-1,2,3,4-tetrahydroquinoxaline-1-carbonyl)thiophen-3-yl)oxy)phenyl)-N-(2-(trimethylammonio)ethyl)propanamido)propanoate (27b). The title compound was obtained as light yellow oil from **26h** in yield of 73%. ^1H NMR (500 MHz, Methanol-d_4): δ 7.61 (d, J = 5.4 Hz, 1H), 7.40 (s, 1H), 6.92 (td, J = 7.3, 1.4 Hz, 1H), 6.86 (d, J = 8.1 Hz, 1H), 6.70 (d, J = 7.6 Hz, 1H), 6.46 (m, 2H), 6.02 (s, 1H), 3.90 (t, J = 5.1 Hz, 2H), 3.82 (m, 2H), 3.68 (m, 2H), 3.61 (s, 2H), 3.48 (m, 4H), 3.20 (s, 9H), 2.94 (s, 2H), 2.43 (t, J = 6.9 Hz, 2H), 2.29 (tt, J = 6.7, 3.7 Hz, 1H), 0.71 (dt, J = 6.6, 3.3 Hz, 2H), 0.34 (m, 2H). ^{13}C NMR (126 MHz, Methanol-d_4): δ 177.49, 173.26, 161.24, 150.42, 149.67, 139.91, 134.93, 132.07, 131.16, 128.57, 126.32, 125.24, 122.79, 121.80, 120.43, 118.65, 118.28, 115.32, 113.05, 62.96, 62.25, 52.55, 48.56, 48.47, 46.01, 40.15, 37.12, 30.77, 27.79, 7.13. IR (KBr) cm^{-1}: 1690.84, 1617.79, 1504.54, 1476.05, 1433.80, 1362.58, 1208.60, 1187.85, 844.83, 558.68. HRMS (ESI^+) m/z calcd for $C_{33}H_{38}Cl_2N_4NaO_5S^+$ $(M + Na)^+$ 695.1832, found 695.1846.

Part 6. Synthesis of intermediate 36 and amine derivatives

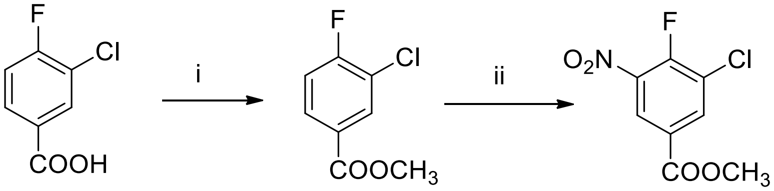

34 **35** **36**

Figure S5. Synthesis of intermediate 36. Reagents and conditions: (i) H_2SO_4, MeOH, 60 °C; (ii) NaOH, 1,4-dioxane/H_2O, rt ;

Methyl 3-chloro-4-fluorobenzoate (35). To a solution of **34** (4.4 g, 25mmol) in MeOH (50 mL) was slowly added H_2SO_4 (1.5 mL). The resulting mixture was stirred at 60 °C overnight. After the reaction mixture cooled to room temperature, the solution was evaporated under vacuum, diluted with water and extracted with ethyl acetate for three times. The organic layer was combined, dried over anhydrous magnesium sulfate, and evaporated under vacuum to afford 4.6 g (98%) of **35** as a colorless oil. ^{1}H NMR (400 MHz, $CDCl_3$): δ 7.90 (dd, J = 8.8, 6.2 Hz, 1H), 7.20 (dd, J = 8.6, 2.5 Hz, 1H), 7.03 (ddd, J = 8.7, 7.6, 2.5 Hz, 1H), 3.93 (s, 3H).

Methyl 3-chloro-4-fluoro-5-nitrobenzoate (36). To a mixture of **35** (4.6 g, 24 mmol) and H_2SO_4 (1.7 mL) was added the solution of H_2SO_4 (2.2 mL) and HNO_3 (2.2 mL) at 0 °C. The resulting mixture was stirred at room temperature overnight. Then the reaction mixture was poured into water, extracted with ethyl acetate, washed with aqueous $NaHCO_3$, H_2O and brine, dried over anhydrous magnesium sulfate, and evaporated under vacuum. The residue was purified by flash column chromatography (petroleum ether/ethyl acetate=50:1) to provide 2.5 g (43%) of **36** as a white solid. ^{1}H NMR (400 MHz, $CDCl_3$): δ 8.68 (d, J = 8.0 Hz, 1H), 7.46 (d, J = 10.2 Hz, 1H), 3.99 (s, 3H).

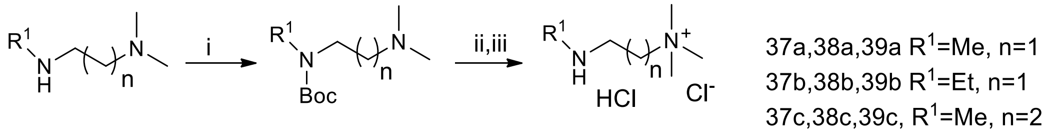

37a-c **38a-c** **39a-c**

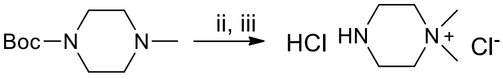

40 **41**

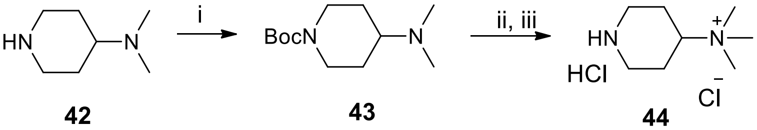

42 **43** **44**

Figure S6. Synthesis of 39a-c, 41, 44. Reagents and conditions: (i) Boc_2O, $NaHCO_3$, THF, H_2O, rt; (ii) MeI, MeCN, rt; (iii) 4N HCl/EA, DCM, rt

General procedure for preparation of compound 38a–c or 43. To a solution of **37a-c** or **42** (20 mmol) in THF (80 mL) and H_2O (80 mL) was added $NaHCO_3$ (2.52g, 30 mmol) followed by Boc_2O (5.13 mL, 30 mmol). The resulting mixture was stirred at room temperature overnight. Then the reaction mixture was evaporated under vacuum, diluted with water, extracted with DCM for three times. The organic layer was combined, dried over anhydrous magnesium sulfate and evaporated under vacuum. The residue was purified by flash column chromatography (DCM/MeOH=10:1) to provide **38a–c** or **43**.

Tert-butyl (2-(dimethylamino)ethyl)(methyl)carbamate (38a). The title compound was synthesized from **37a** according to the general procedure in yield of 81%. ^{1}H NMR (300 MHz, $CDCl_3$): δ 3.30 (m, 2H), 2.86 (s, 3H), 2.40 (m, 2H), 2.53 (s, 6H), 1.45 (s, 9H).

Tert-butyl (2-(dimethylamino)ethyl)(ethyl)carbamate (38b). The title compound was synthesized from **37b** according to the general procedure in yield of 52%. ^{1}H NMR (400 MHz, $CDCl_3$): δ 3.26 (m, 4H), 2.42 (m, 2H), 2.27 (s, 6H), 1.46 (s, 9H), 1.10 (t, J = 7.1 Hz, 3H).

Tert-butyl (3-(dimethylamino)propyl)(methyl)carbamate (38c). The title compound was synthesized from **37c** according to the general procedure in yield of 76%. ^{1}H NMR (400 MHz, $CDCl_3$): δ 3.24 (m, 2H), 2.85 (s, 3H), 2.29 (s, 2H), 2.25 (s, 6H), 1.70 (m, 2H), 1.46 (s, 9H).

Tert-butyl 4-(dimethylamino)piperidine-1-carboxylate (43). The title compound was synthesized from **42** according to the general procedure in yield of 75%. ^{1}H NMR (400 MHz, $CDCl_3$): δ 4.12 (m, 1H), 2.70 (m, 2H), 2.28 (s, 8H), 1.79 (m, 2H), 1.46 (s, 9H), 1.37 (qd, J = 12.5, 4.4 Hz, 2H).

General procedure for preparation of compound 39a–c, **41**, **44.** To a solution of **38a-c** or **40** or **43** (15 mmol) in MeCN (50 mL) was added CH_3I (1.87 mL, 30 mmol). The resulting mixture was stirred at room temperature overnight. Then the reaction mixture was evaporated under vacuum and dissolved in DCM to which 4N HCl/EA (20 mL) was added. The resulting mixture was stirred at room temperature overnight. The solid formed was filtered, washed with DCM, dried under vacuum to afford **39a–c**, **41**, **44**.

N,N,N-Trimethyl-2-(methylamino)ethanaminium chloride hydrochloride (39a). The title compound was synthesized from **38a** according to the general procedure in yield of 88%. ^{1}H NMR (300 MHz, $CDCl_3$): δ 3.72 (m, 2H), 3.42 (m, 2H), 3.16 (s, 9H), 2.58 (s, 3H).

2-(Ethylamino)-N,N,N-trimethylethanaminium chloride hydrochloride (39b). The title compound was synthesized from **38b** according to the general procedure in yield of 76%. ^{1}H NMR (400 MHz, $CDCl_3$): δ 3.91 (s, 2H), 3.71 (t, J = 6.4 Hz, 2H), 3.49 (s, 9H), 3.38 (q, J = 7.0 Hz, 2H), 1.15 (t, J = 7.0 Hz, 3H).

N,N,N-Trimethyl-3-(methylamino)propan-1-aminium chloride hydrochloride (39c). The title compound was synthesized from **38c** according to the general procedure in yield of 84%. ^{1}H NMR (400 MHz, Methanol-d_4): δ 3.52 (m, 2H), 3.22 (s, 9H), 3.11 (m, 2H), 2.77 (s, 3H), 2.24 (m, 2H).

1,1-Dimethylpiperazin-1-ium chloride hydrochloride (41). The title compound was synthesized from **40** according to the general procedure in yield of 76%. ^{1}H NMR (300 MHz, $CDCl_3$): δ 3.70 (m, 4H), 3.55 (m, 4H), 3.26 (s, 6H)

N,N,N-Trimethylpiperidin-4-aminium chloride hydrochloride (44). The title compound was synthesized from **43** according to the general procedure in yield of 63%.1H NMR (400 MHz, Methanol-d_4): δ 3.86 (dd, J = 13.6, 8.6 Hz, 1H), 3.67 (d, J = 13.3 Hz, 2H), 3.21 (m, 9H), 2.51 (d, J = 13.4 Hz, 2H), 2.08 (m, 2H).

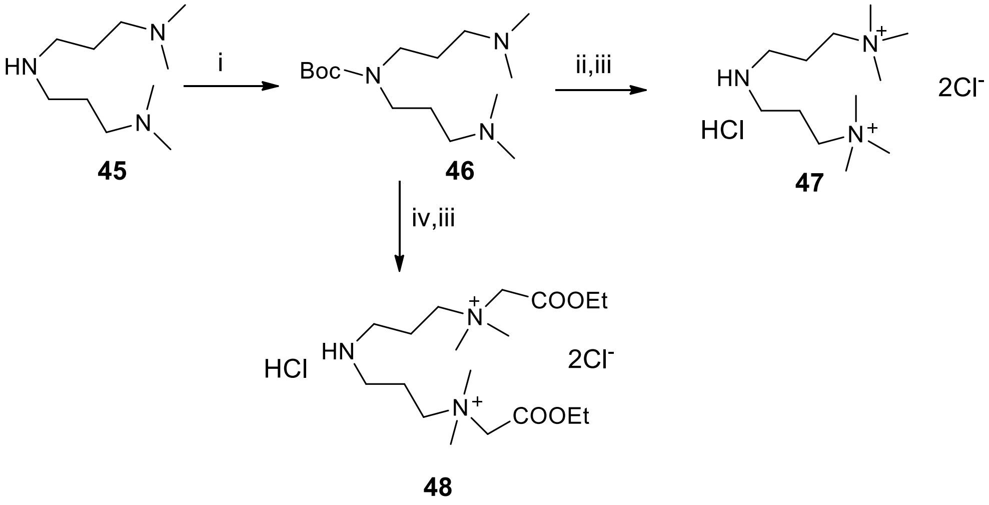

Figure S7. Synthesis of 47, 48. Reagents and conditions: (i) Boc_2O, $NaHCO_3$, THF, H_2O, rt; (ii) MeI, MeCN, rt; (iii) 4N HCl/EA, DCM, rt; (iv) ethyl 2-bromoacetate, MeCN, rt.

Tert-butyl bis(3-(dimethylamino)propyl)carbamate (46). To a solution of **45** (1 g, 5.3 mmol) in THF (15 mL) and H_2O (15 mL) was added $NaHCO_3$ (0.67 g, 8.0 mmol) followed by Boc_2O (1.47 mL, 6.4 mmol). The resulting mixture was stirred at room temperature overnight. Then the reaction mixture was evaporated under vacuum, diluted with water, extracted with DCM for three times. The organic layer was combined, dried over anhydrous magnesium sulfate and evaporated under vacuum. The residue was

purified by flash column chromatography (DCM/MeOH/ (saturate NH_3 in MeOH) =30:2:1) to provide **46** in a yield of 24%. ^{1}H NMR (400 MHz, $CDCl_3$): δ 3.21 (m, 4H), 2.26 (t, J = 7.4 Hz, 4H), 2.22 (s, 12H), 1.70 (m, 4H), 1.46 (s, 9H).

3,3'-Azanediylbis(N,N,N-trimethylpropan-1-aminium) dichloro hydrochloride (47). To a solution of **46** (0.19 g, 0.66 mmol) in MeCN (10 mL) was added CH_3I (162μl, 2.60 mmol). The resulting mixture was stirred at room temperature overnight. Then the reaction mixture was evaporated under vacuum and dissolved in DCM to which 4N HCl/EA (2 mL) was added. The resulting mixture was stirred at room temperature overnight. Then the reaction mixture was evaporated under vacuum, dissolved in DCM/MeCN and cooled to -10 ℃. The solid formed was filtered, washed with DCM, and dried under vacuum to afford **47** in yield of 65%.^{1}H NMR (400 MHz, Methanol-d_4): δ 3.62 (m, 4H), 3.26 (d, J = 7.9 Hz, 4H), 3.24 (s, 18H), 2.32 (m, 4H).

3,3'-Azanediylbis(N-(2-ethoxy-2-oxoethyl)-N,N-dimethylpropan-1-aminium) dichloro hydrochloride (48). To a solution of **46** (0.19 g,0.66 mmol) in MeCN (10 mL) was added ethyl 2-bromoacetate (300 μl, 2.60 mmol). The resulting mixture was stirred at room temperature overnight. Then the reaction mixture was evaporated under vacuum and dissolved in DCM to which 4N HCl/EA (2 mL) was added. The resulting mixture was stirred at room temperature overnight. Then the reaction mixture was evaporated under vacuum, dissolved in DCM/MeCN and cooled to -10 ℃. The solid formed was filtered, washed with DCM, and dried under vacuum to afford **48** in yield of 69%. ^{1}H NMR (400 MHz, DMSO-d_6): δ 4.55 (s, 4H), 4.24 (q, J = 6.5, 5.9 Hz, 4H), 3.71 (s, 4H), 3.27 (s, 12H), 3.00 (s, 4H), 2.18 (s, 4H), 1.26 (t, J = 7.0 Hz, 6H).

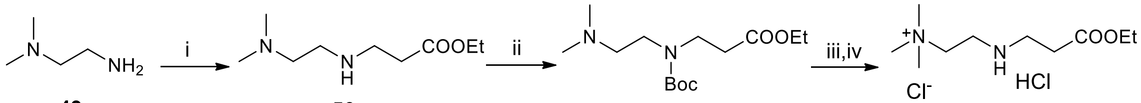

Figure S8. Synthesis of 52. Reagents and conditions: (i) Ethyl acrylate, DCM, rt; (ii) Boc_2O, $NaHCO_3$, THF, H_2O, rt; (iii) MeI, MeCN, rt; (iv) 4N HCl/EA, DCM, rt.

Ethyl 3-((2-(dimethylamino)ethyl)amino)propanoate (50). To a solution of **49** (2 g, 22.7 mmol) in DCM (150 mL) was slowly added ethyl acrylate (1.21 mL, 11.4 mmol). The resulting mixture was stirred overnight. Then the reaction mixture was evaporated under vacuum to give **50** in quantitative yield. ^{1}H NMR (400 MHz, $CDCl_3$): δ 4.15 (q, J = 7.1 Hz, 2H), 2.94 (t, J = 6.6 Hz, 2H), 2.75 (t, J = 6.1 Hz, 2H), 2.57 (t, J = 6.6 Hz, 2H), 2.47 (t, J = 6.1 Hz, 2H), 2.26 (s, 6H), 1.26 (t, J = 7.1 Hz, 3H).

Ethyl 3-((tert-butoxycarbonyl)(2-(dimethylamino)ethyl)amino)propanoate (51). To a solution of **50** (2.1 g, 11.2 mmol) in THF (75 mL) and H_2O (75 mL) was added $NaHCO_3$ (1.4 g, 16.7 mmol) followed by Boc_2O (3.1 mL, 13.4 mmol). The resulting mixture was

stirred at room temperature overnight. Then the reaction mixture was evaporated under vacuum, diluted with water, extracted with DCM for three times. The organic layer was combined, dried over anhydrous magnesium sulfate and evaporated under vacuum. The residue was purified by flash column chromatography (DCM/MeOH =10:1) to provide **51** in a yield of 46%. ^{1}H NMR (400 MHz, $CDCl_3$): δ 4.13 (q, J = 7.1 Hz, 2H), 3.49 (s, 2H), 3.31 (s, 2H), 2.57 (s, 2H), 2.42 (s, 2H), 2.25 (s, 6H), 1.46 (s, 9H), 1.26 (t, J = 7.1 Hz, 3H).

2-((3-Ethoxy-3-oxopropyl)amino)-N,N,N-trimethylethanaminium chloride hydrochloride (52). To a solution of **51** (1.2 g, 4.2 mmol) in MeCN (50 mL) was added CH_3I (520 µl, 8.4 mmol). The resulting mixture was stirred at room temperature overnight. The reaction mixture was then evaporated under vacuum and dissolved in DCM to which 4N HCl/EA (20 mL) was added. The resulting mixture was stirred at room temperature overnight. Then the reaction mixture was evaporated under vacuum, dissolved in DCM/MeCN and cooled to -10 ℃. The solid formed was filtered, washed with DCM, and dried under vacuum to afford **52** in yield of 69%. ^{1}H NMR (400 MHz, Methanol-d_4): δ 4.24 (q, J = 7.1 Hz, 2H), 3.90 (m, 2H), 3.75 (m, 2H), 3.49 (t, J = 6.7 Hz, 2H), 3.35 (s, 9H), 2.94 (m, 2H), 1.31 (t, J = 7.1 Hz, 3H).

Part 7. Raw data *in vitro* leading to EC_{50} of 2, 9a, 26a

For **2**:

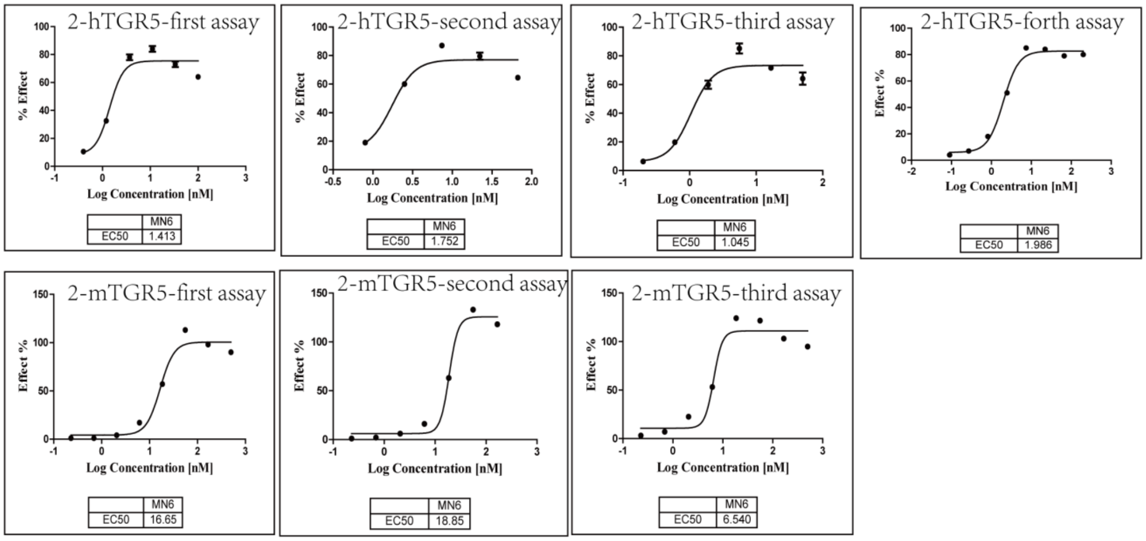

For **9a**:

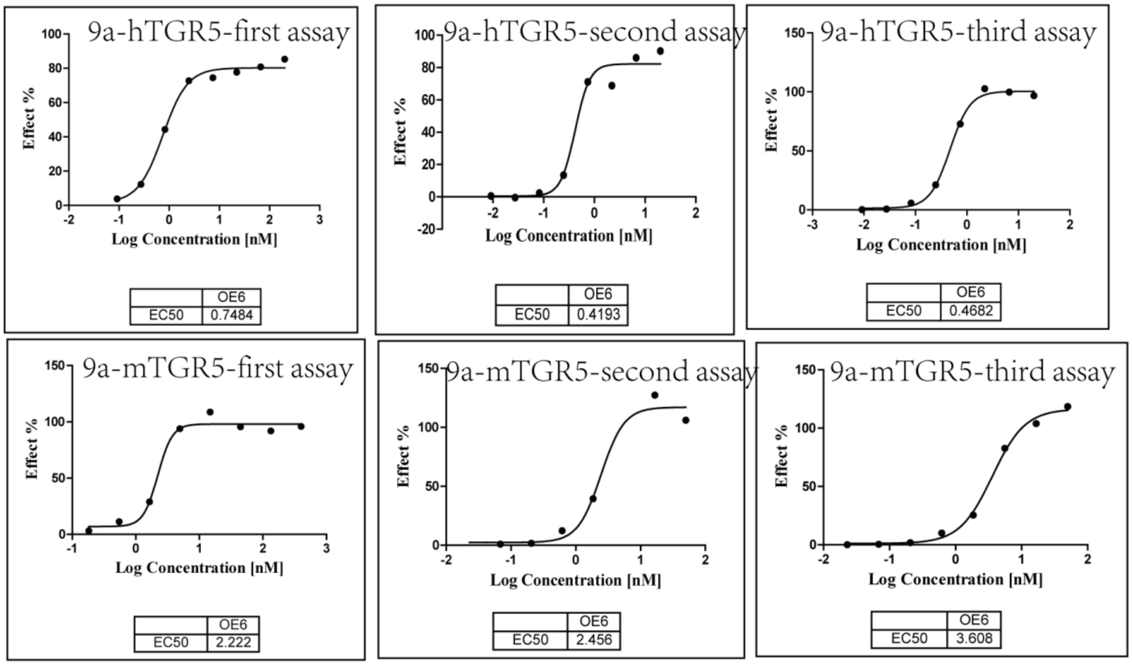

For **26a**:

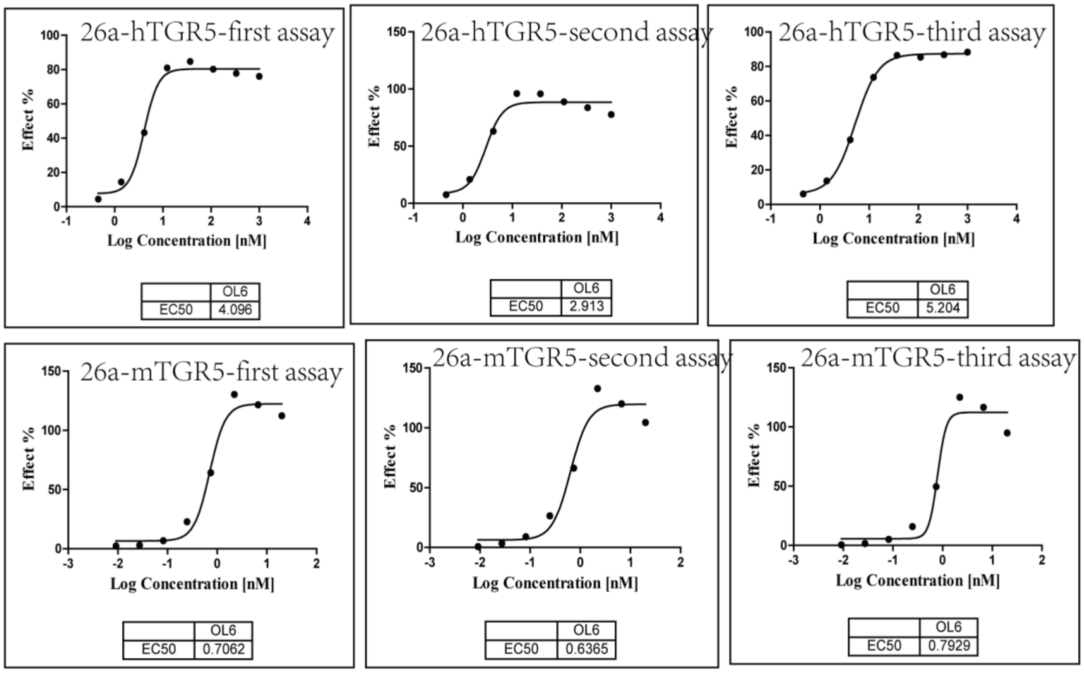
26a-hTGR5-first assay
Effect %
Log Concentration [nM]
OL6
EC50 4.096
26a-hTGR5-second assay
Effect %
Log Concentration [nM]
OL6
EC50 2.913
26a-hTGR5-third assay
Effect %
Log Concentration [nM]
OL6
EC50 5.204
26a-mTGR5-first assay
Effect %
Log Concentration [nM]
OL6
EC50 0.7062
26a-mTGR5-second assay
Effect %
Log Concentration [nM]
OL6
EC50 0.6365
26a-mTGR5-third assay
Effect %
Log Concentration [nM]
OL6
EC50 0.7929

Part 8. Total cholesterol level after the 18-day treatment of 26a in *ob/ob* mice.

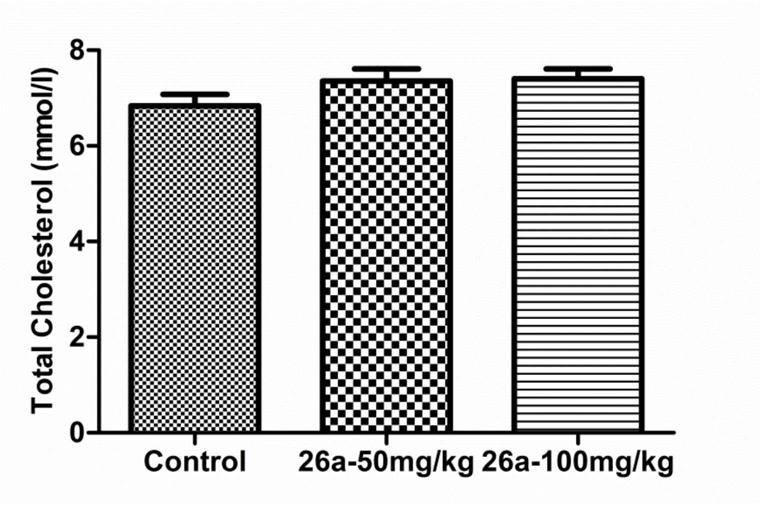

Figure S9. Total cholesterol level after the 18-day treatment of 26a in ob/ob mice. Compound 26a (50 mg/kg or 100 mg/kg) and 0.25% CMC (control) were administered (p.o.) to ob/ob mice (male, n = 10) for 18 days. Error bar indicates SEM.

Part 9. HPLC purity of the final compounds

Compd	Purity (%)	t_R(min)	Compd	Purity (%)	t_R(min)
**9a**	98.27	17.72	**20**	97.57	11.48
**9b**	99.21	17.47	**23**	98.37	12.80
**9c**	96.37	17.86	**26a**	96.61	13.30
**9d**	97.79	16.93	**26b**	95.18	13.63
**9e**	99.42	16.58	**26c**	98.30	13.28
**9f**	97.96	19.15	**26d**	95.26	13.06
**9g**	99.46	17.33	**26e**	98.19	13.07
**9h**	99.21	16.48	**26f**	100	11.24
**9i**	98.70	18.01	**27a**	96.49	12.18
**9j**	98.71	17.32	**27b**	95.80	12.96
**9k**	97.04	17.18	**30**	95.66	13.34
**10**	98.45	17.09	**33**	99.40	13.53

Table S6. HPLC purity of the final compounds. HPLC analyses were performed on an Agilent 1200 series LC system (Agilent ChemStation, ZORBAX SB-C18, 5 μM, 4.6 × 150 mm, 30 ℃, UV 240 or 254 nm, 1.0 mL/min). MeOH/H_2O (0.1%TFA) gradient: 30-90%, 0-15min; 90%, 15-25min.

Part 10. Copies of ^{1}H NMR, ^{13}C NMR of the final compounds

^{1}H NMR of **9a**

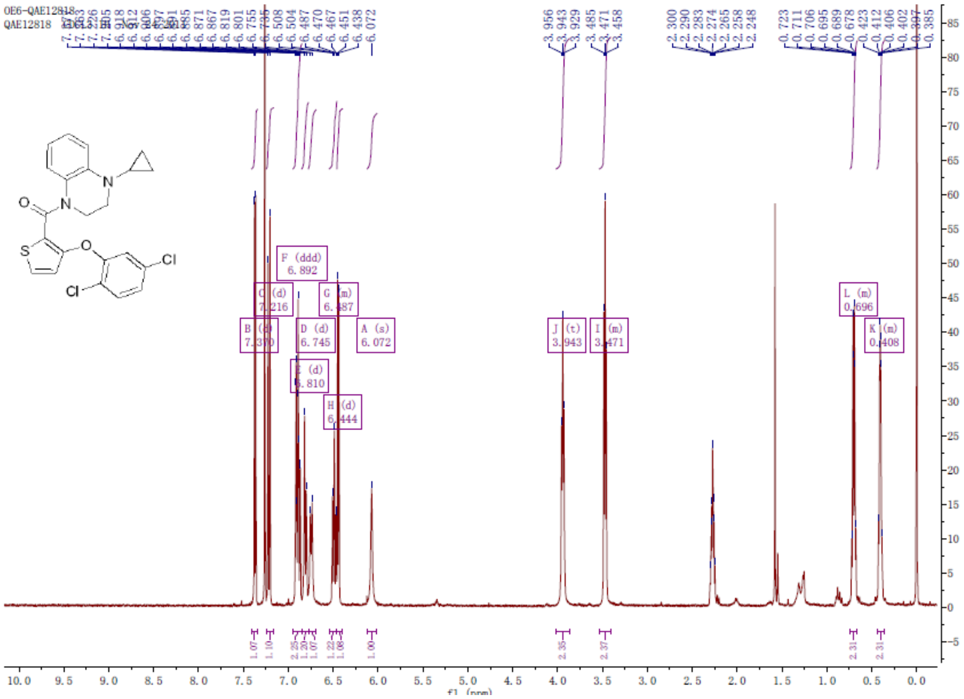

^{13}C NMR of **9a**

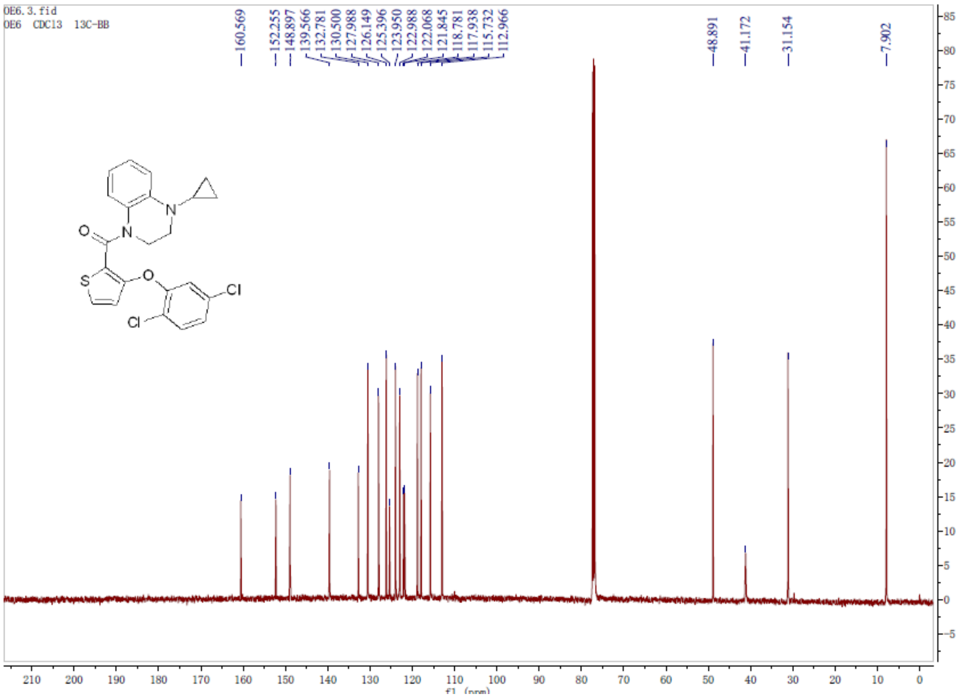

^{1}H NMR of **9b**

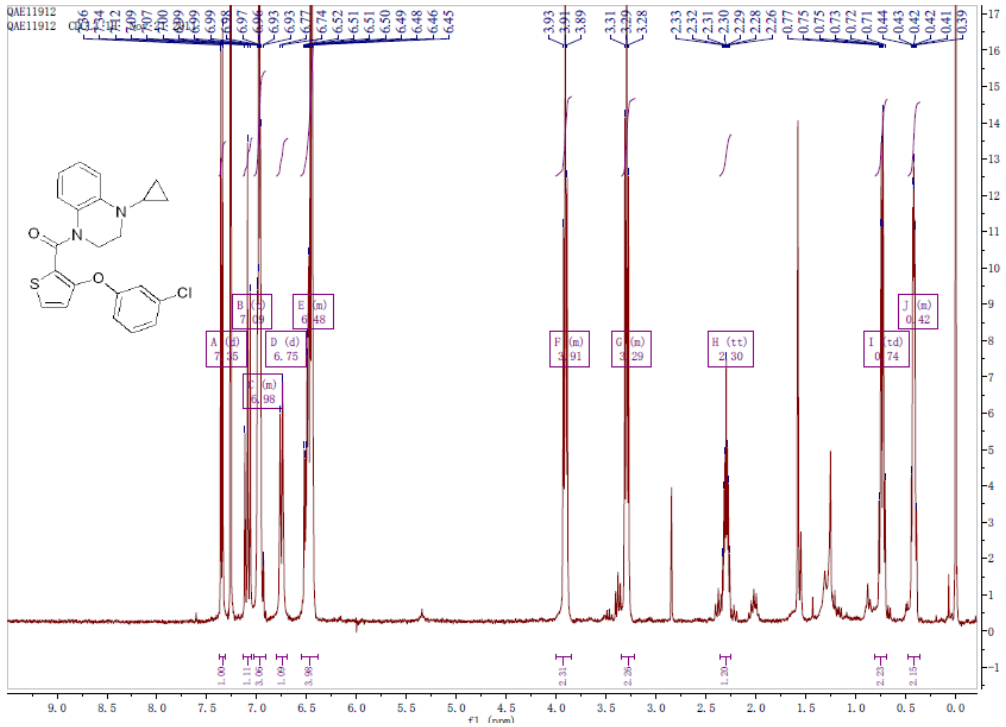

^{13}C NMR of **9b**

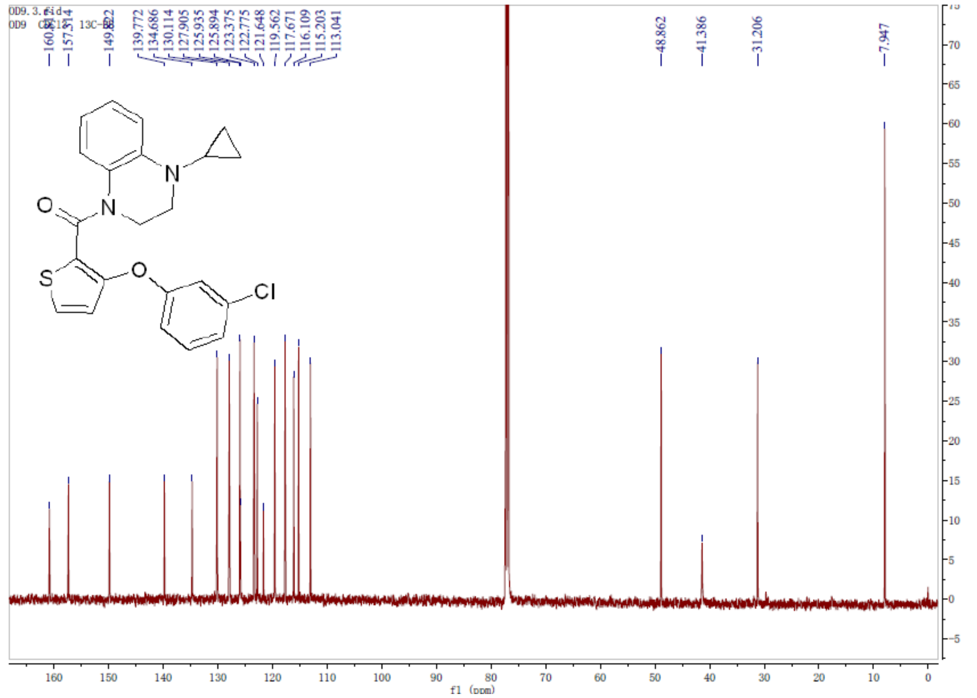

^{1}H NMR of **9c**

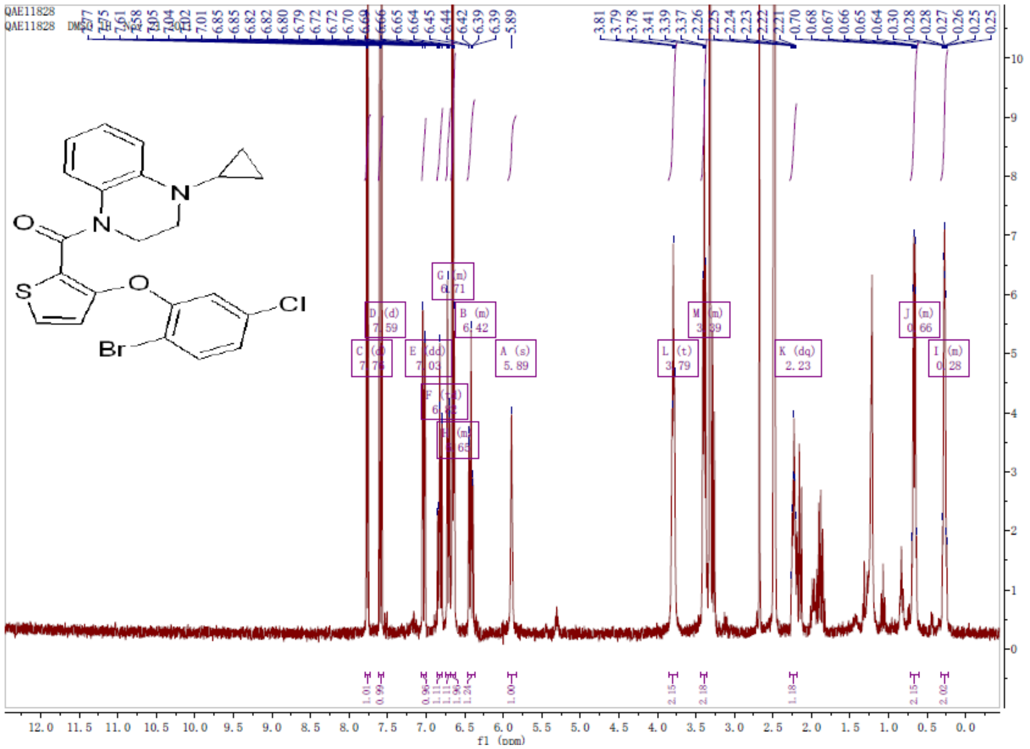

^{13}C NMR of **9c**

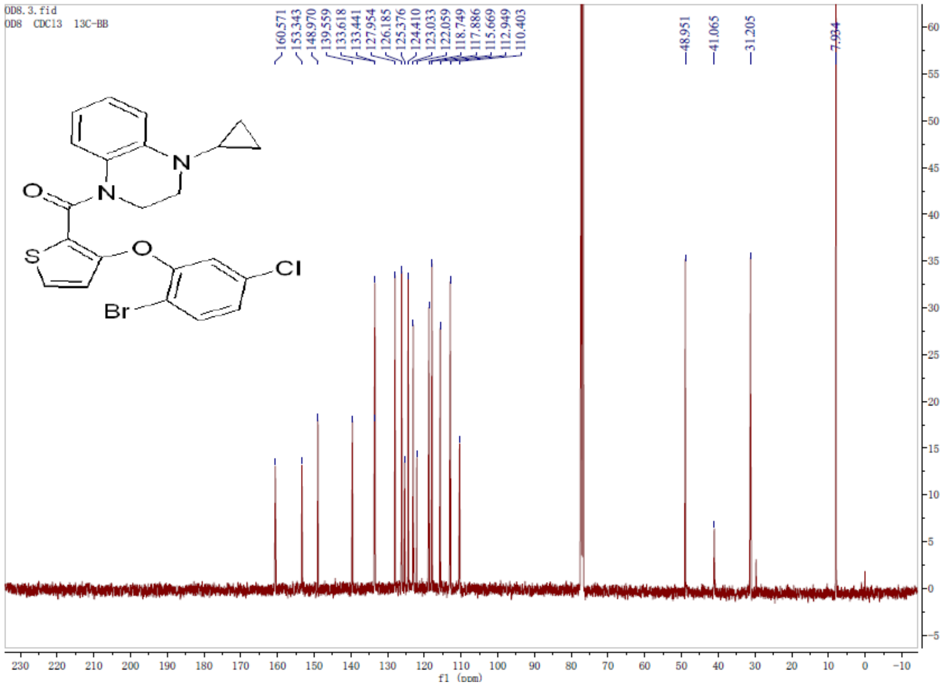

^{1}H NMR of **9d**

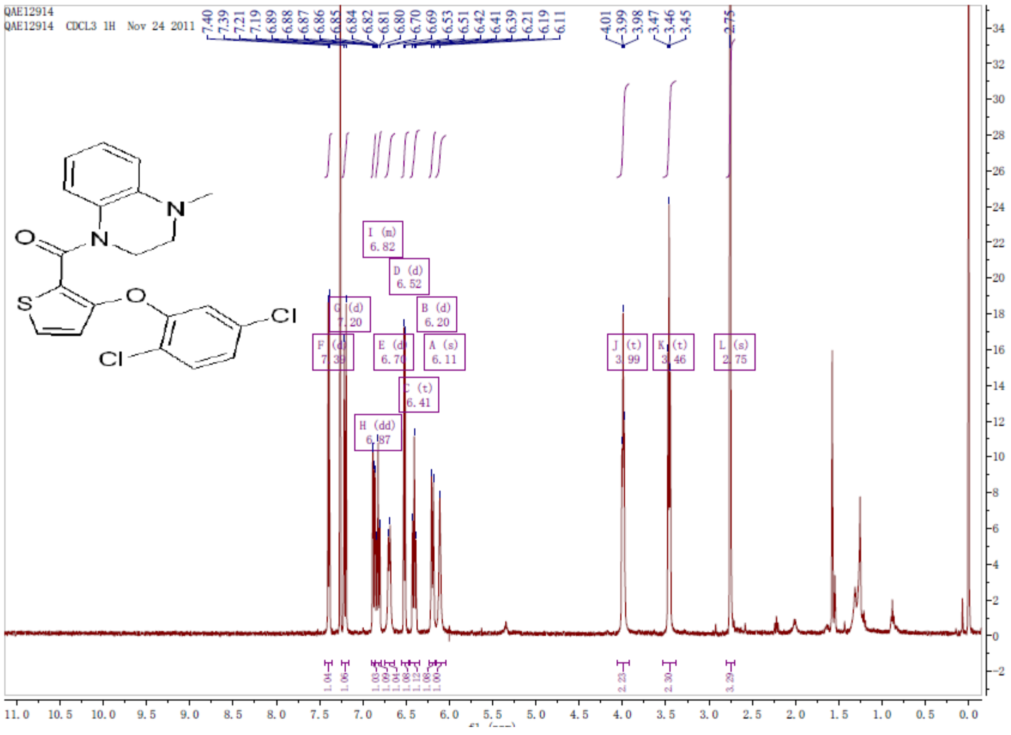

^{13}C NMR of **9d**

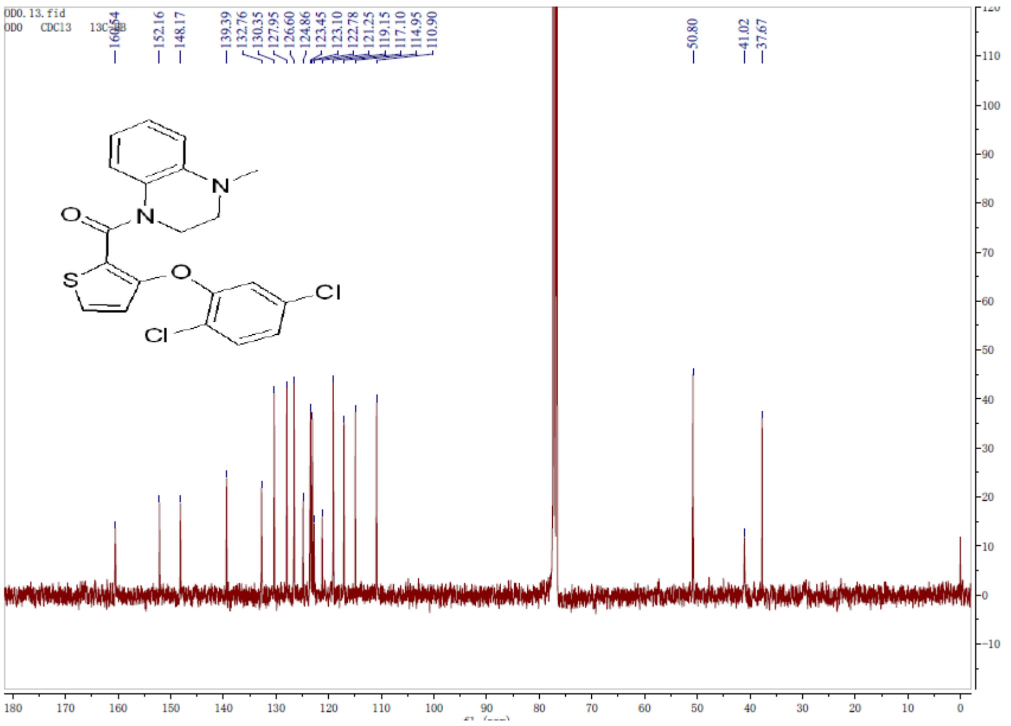

^{1}H NMR of **9e**

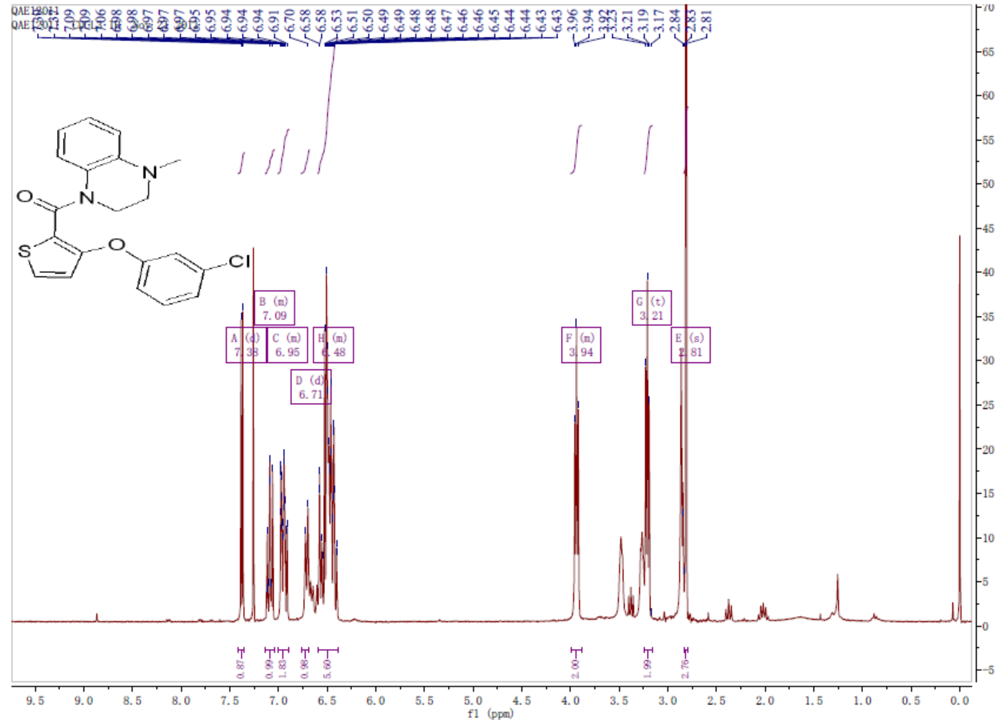

^{13}C NMR of **9e**

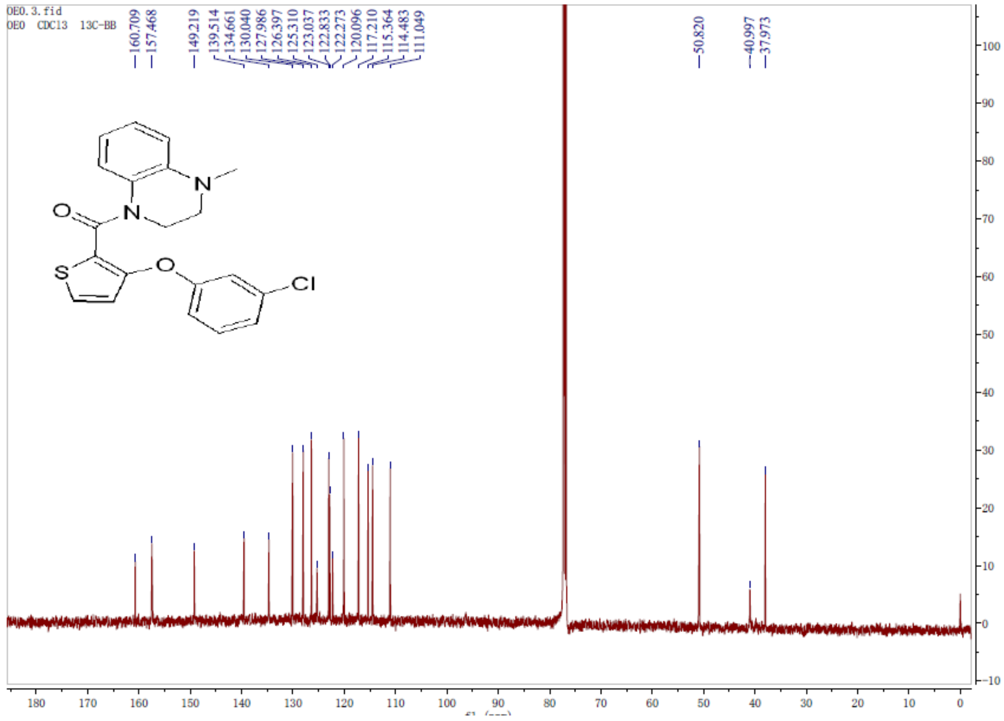

^{1}H NMR of **9f**

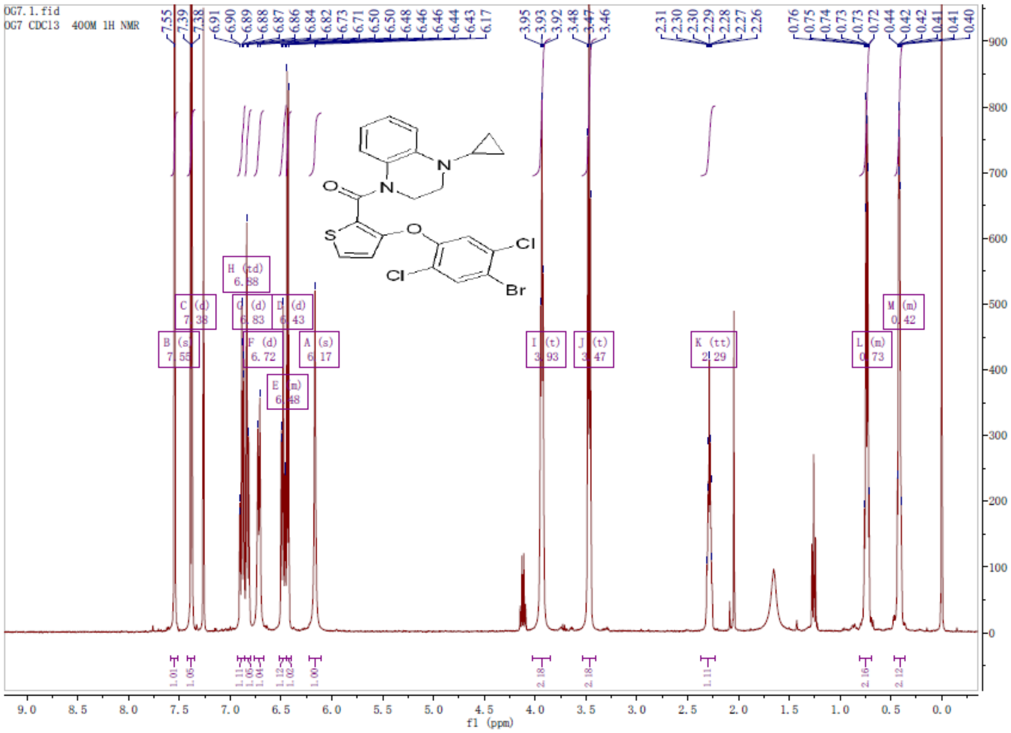

^{13}C NMR of **9f**

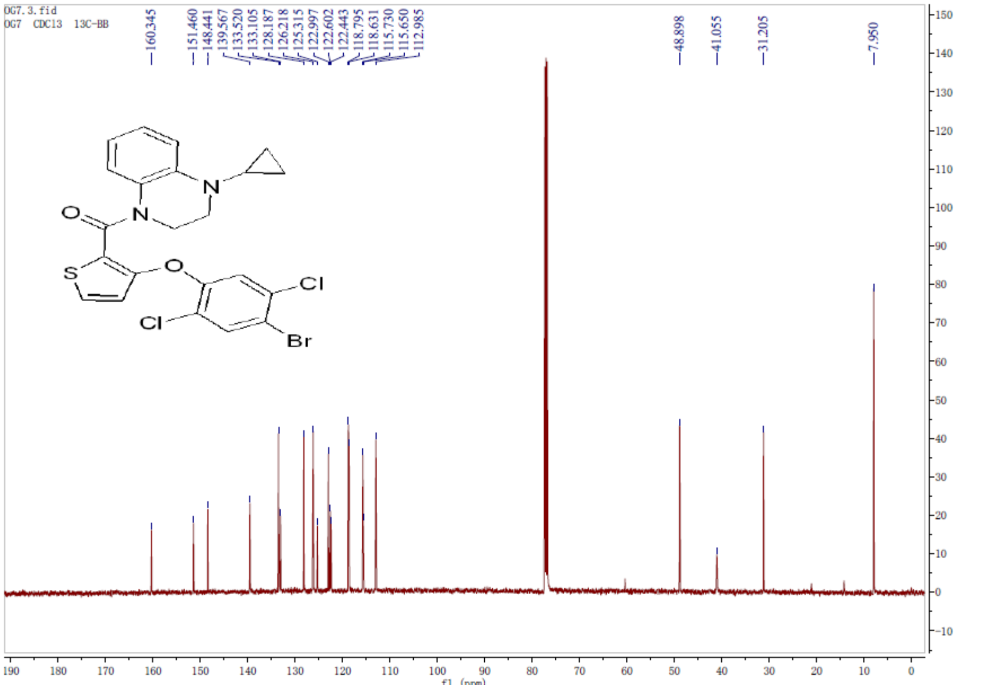

^{1}H NMR of **9g**

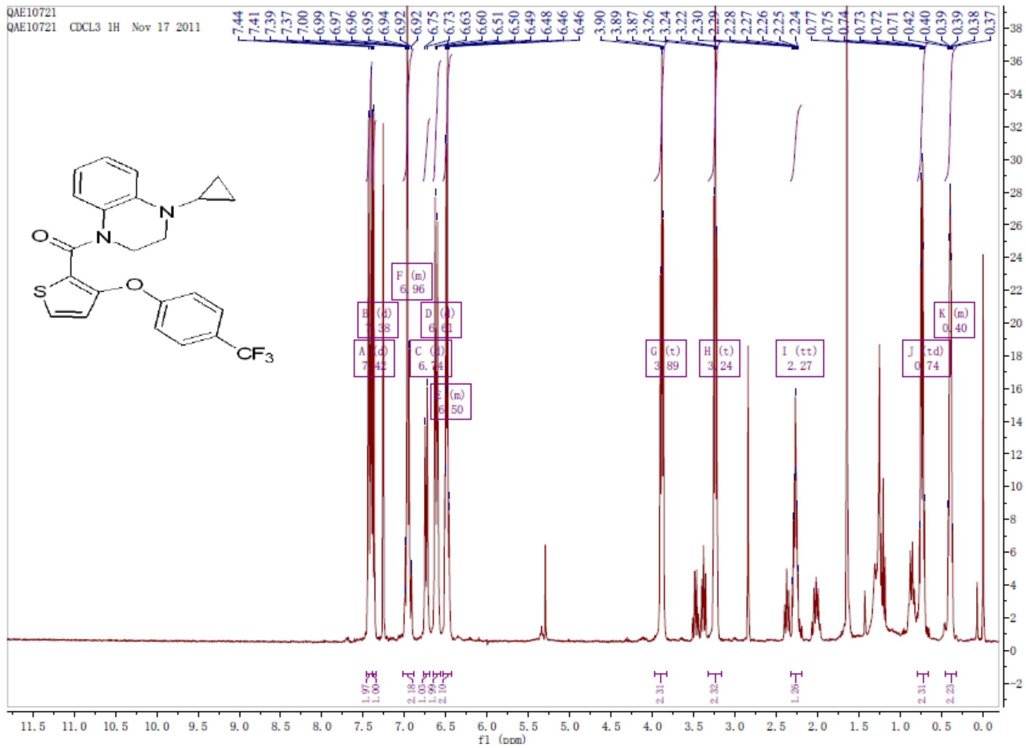

^{13}C NMR of **9g**

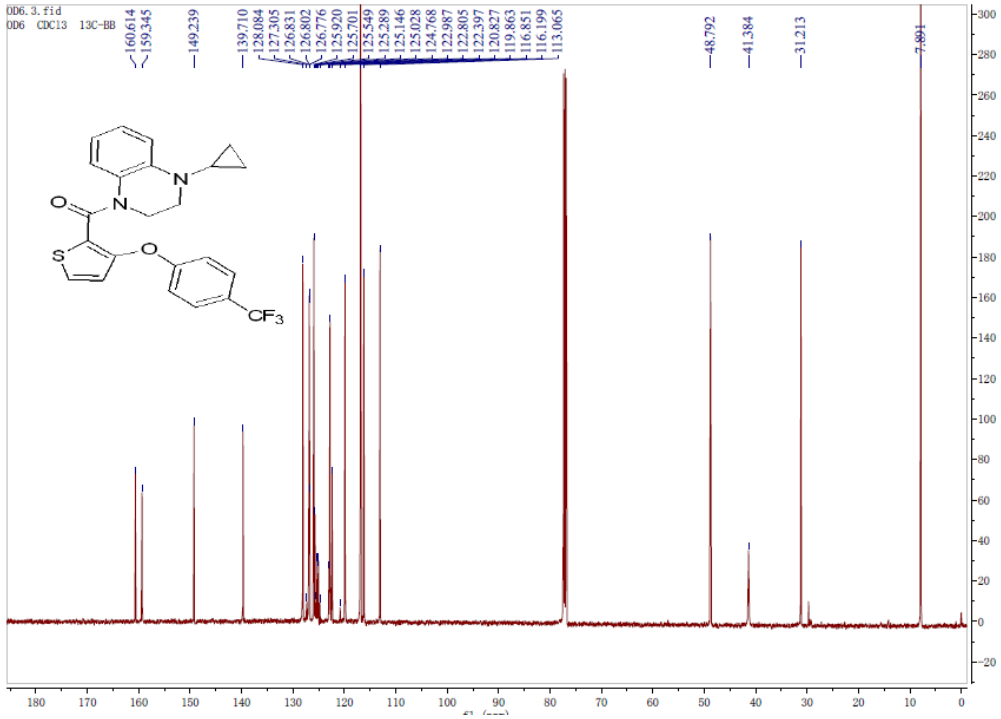

^{1}H NMR of **9h**

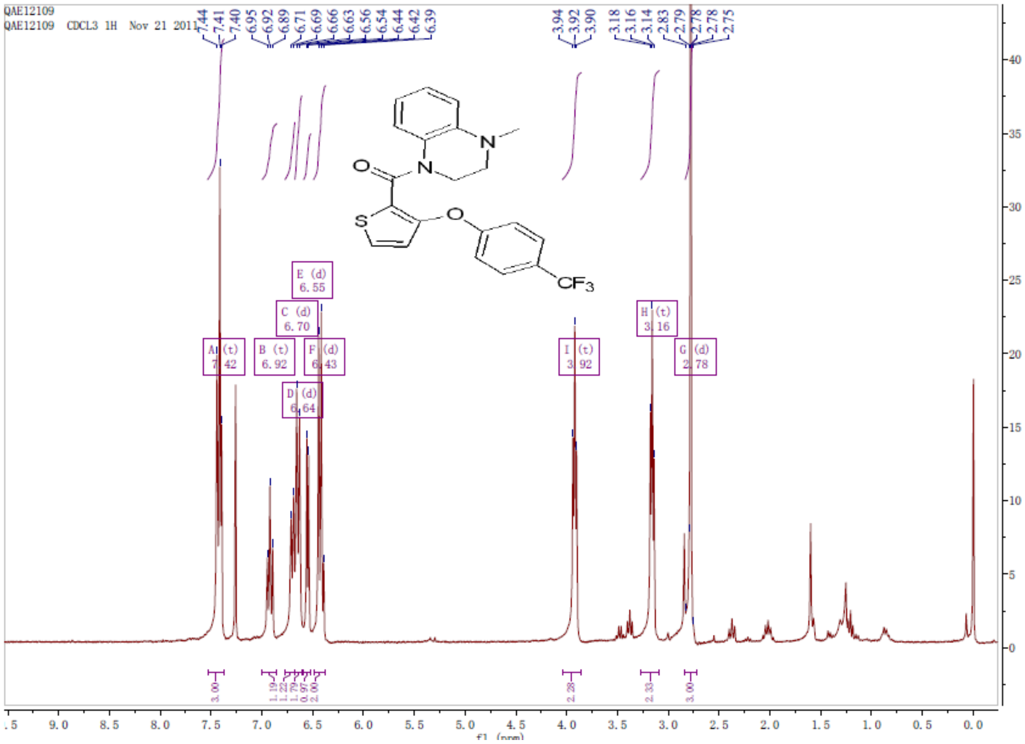

^{13}C NMR of **9h**

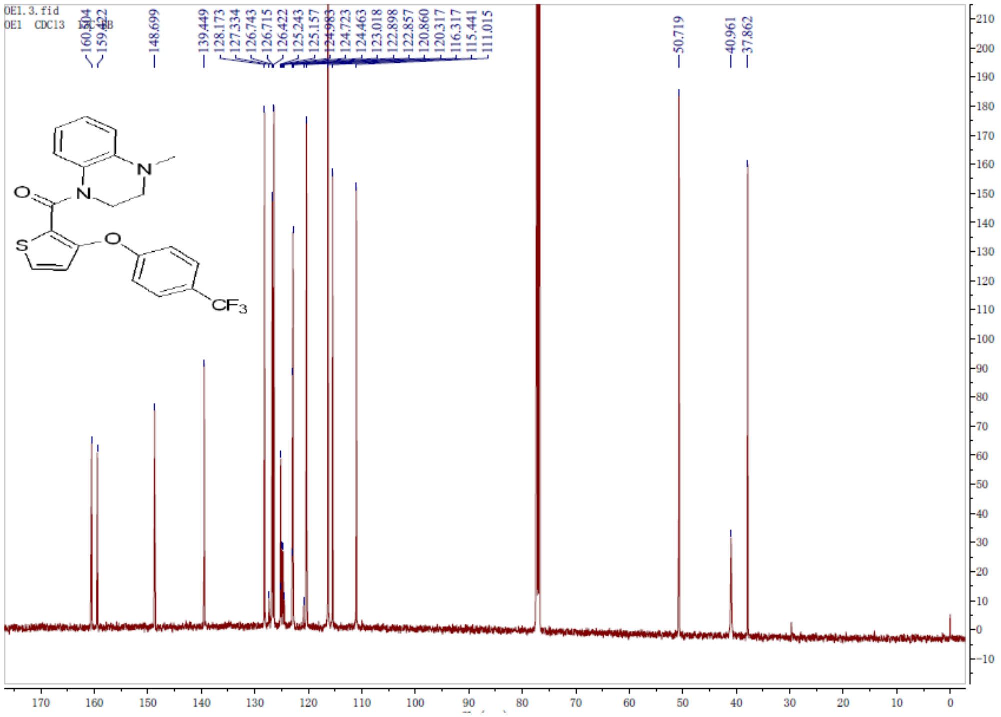

^{1}H NMR of **9i**

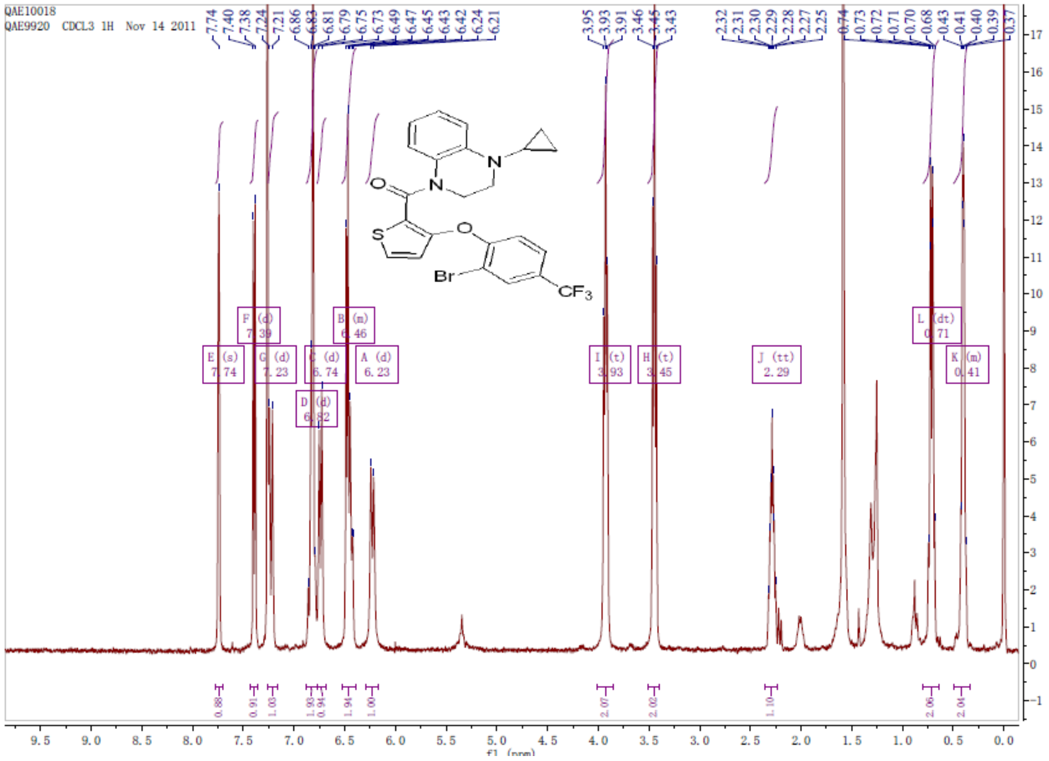

^{13}C NMR of **9i**

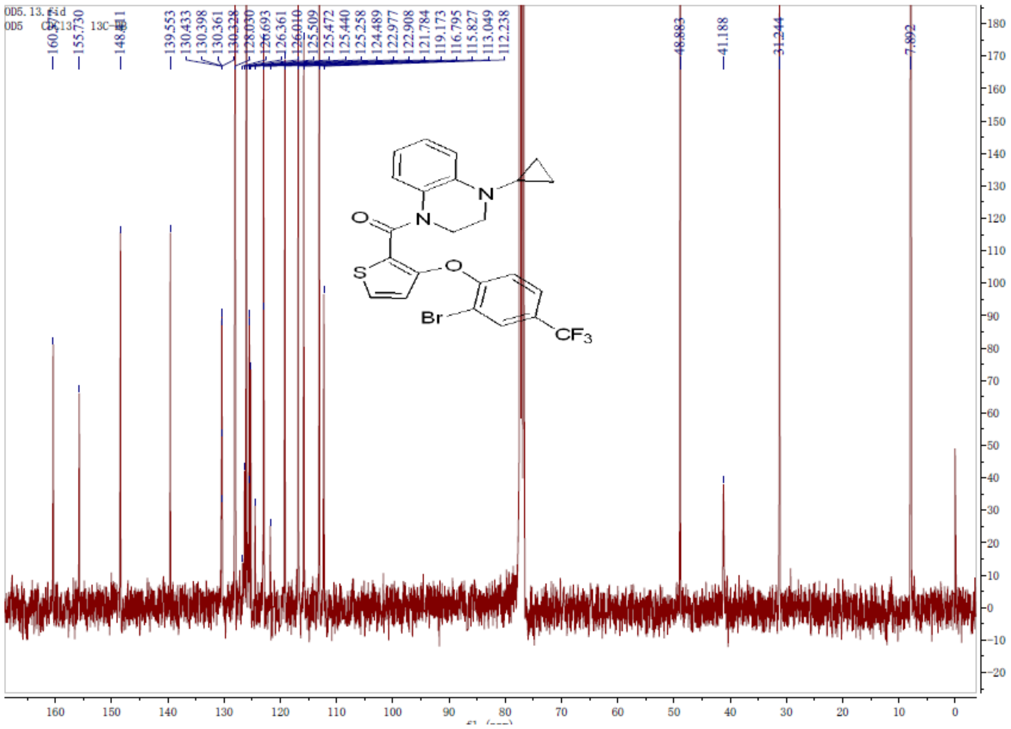

^{1}H NMR of **9j**

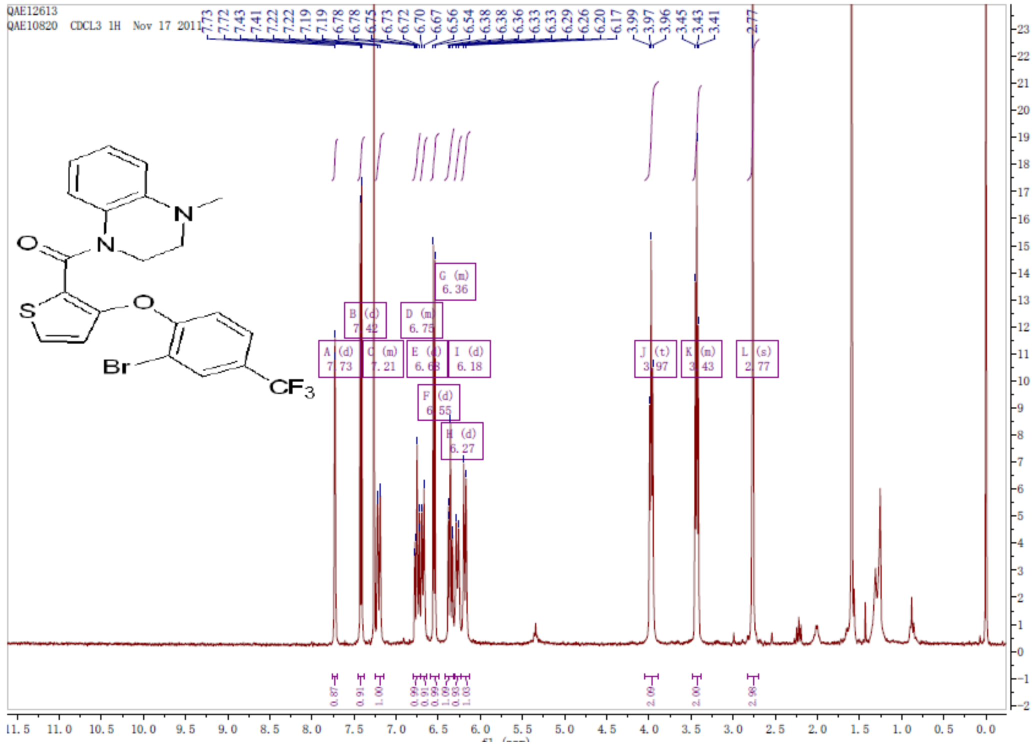

^{13}C NMR of **9j**

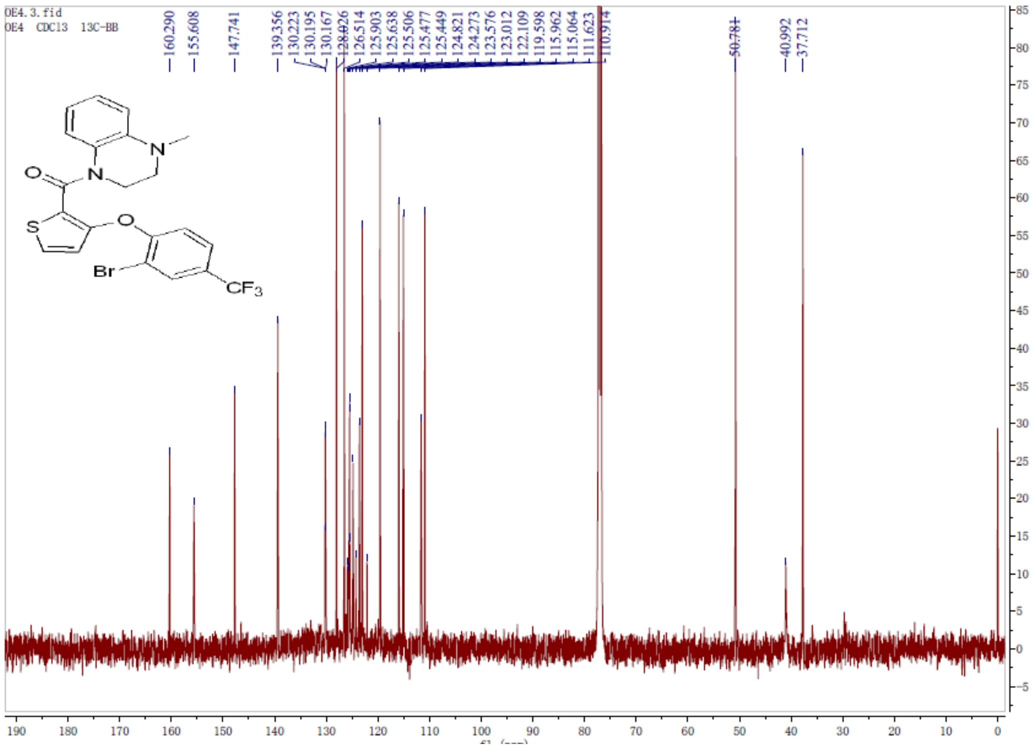

^{1}H NMR of **9k**

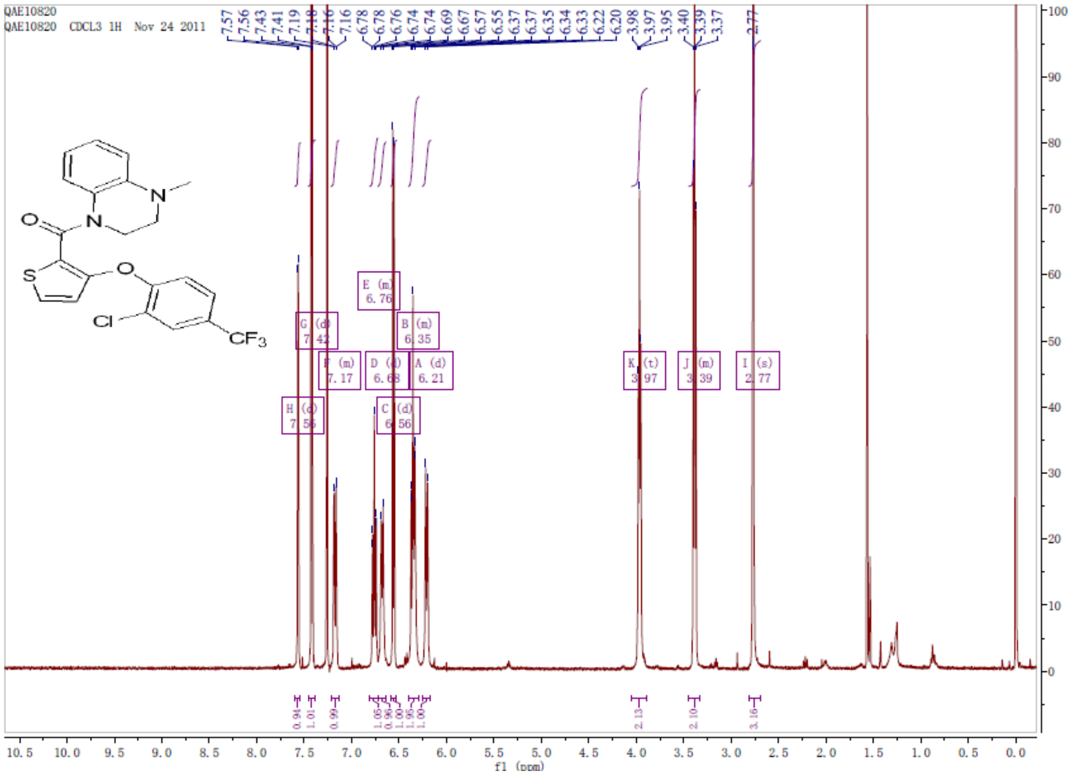

^{13}C NMR of **9k**

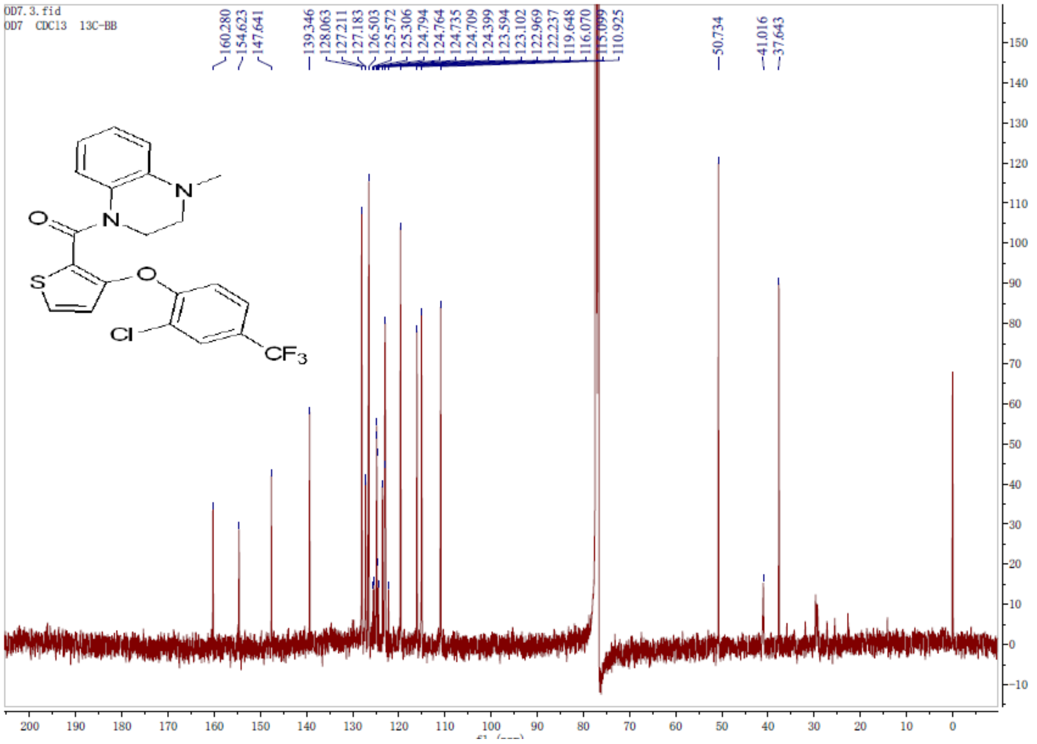

^{1}H NMR of **10**

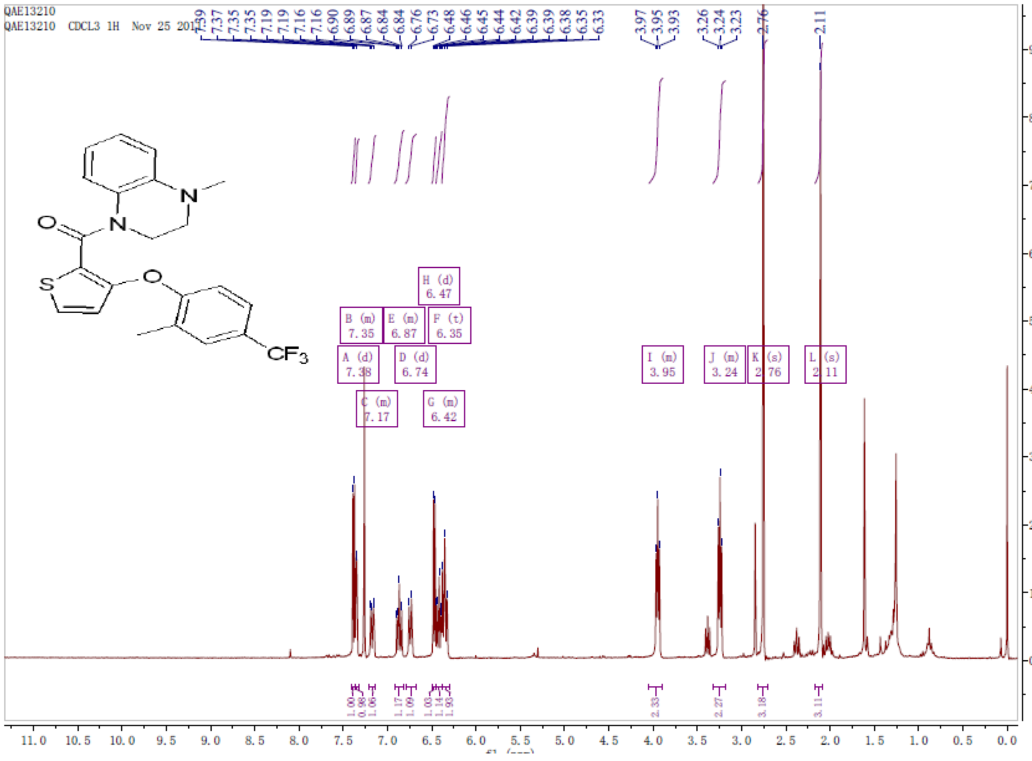

^{13}C NMR of **10**

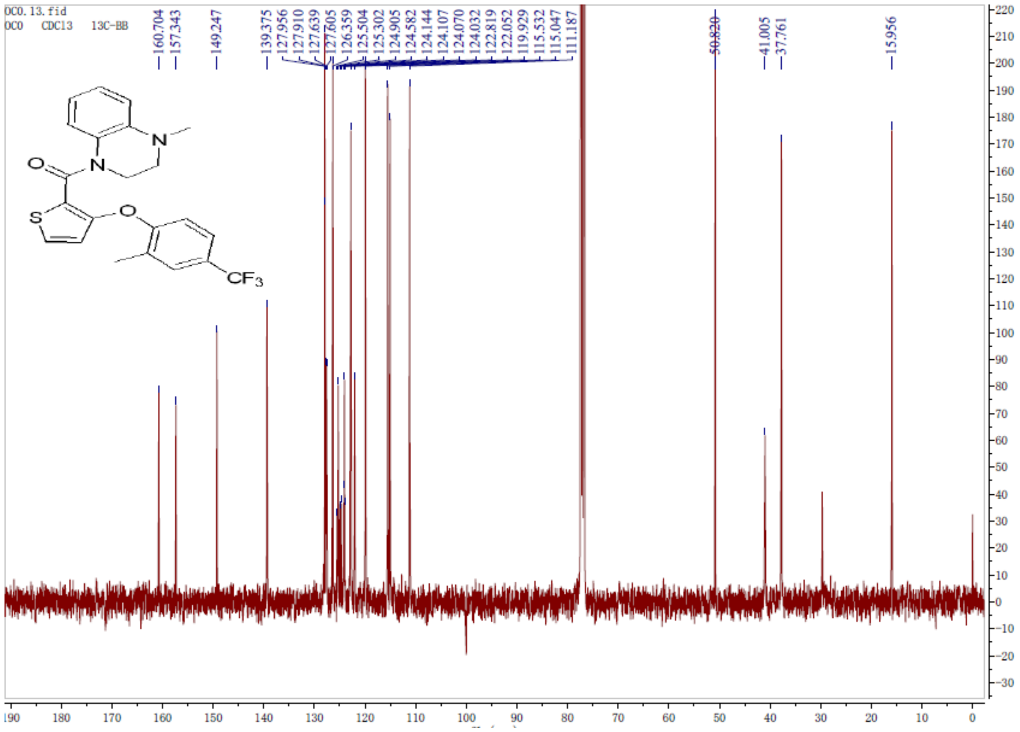

^{1}H NMR of **20**

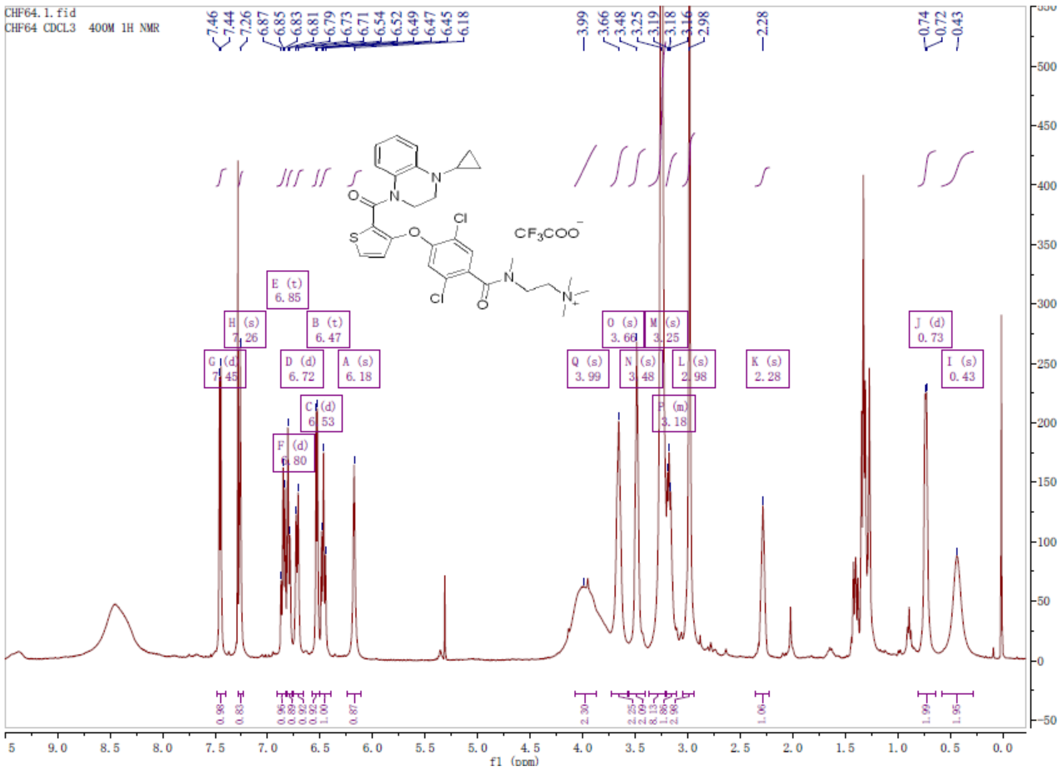

^{13}C NMR of **20**

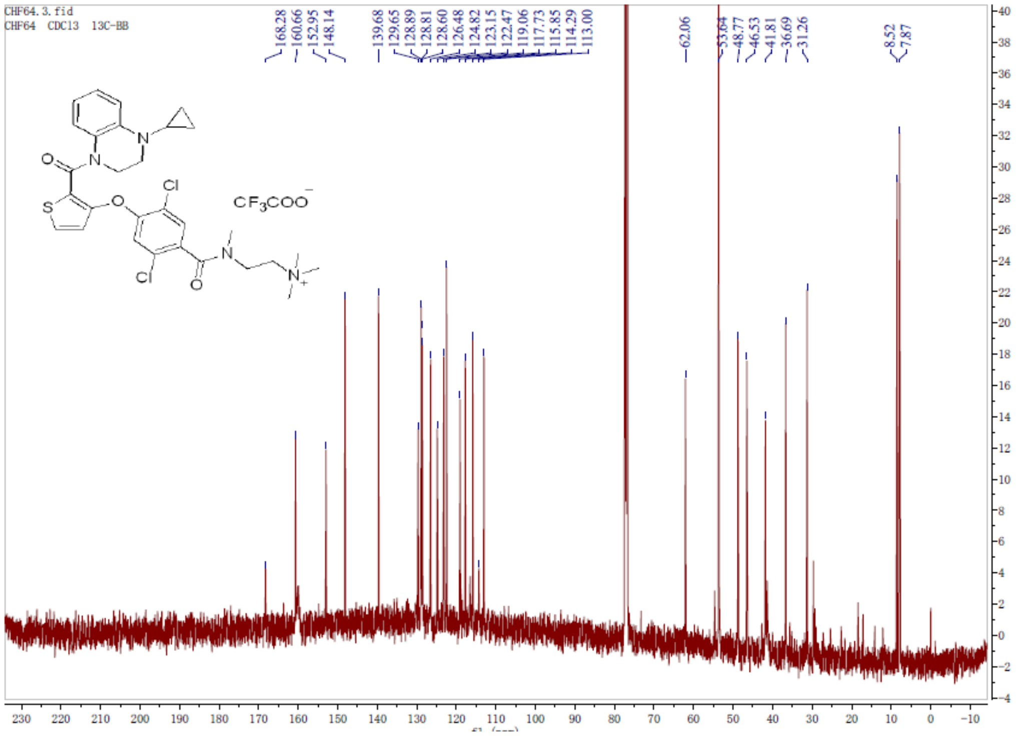

^{1}H NMR of **23**

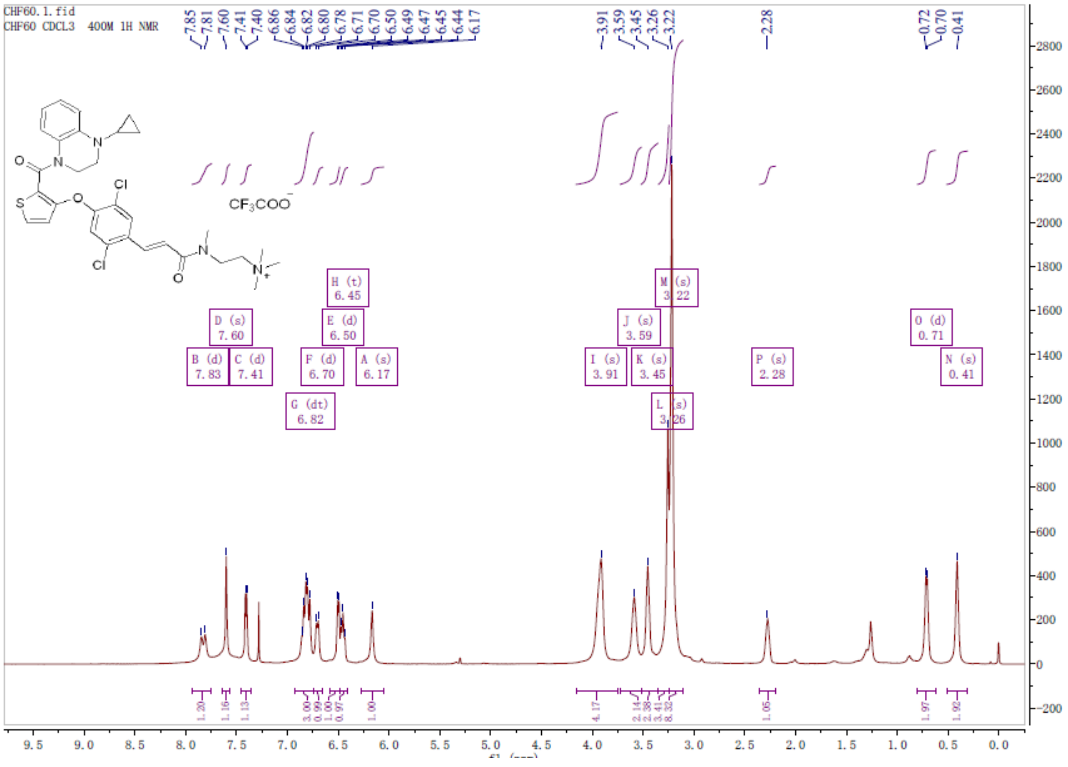

^{13}C NMR of **23**

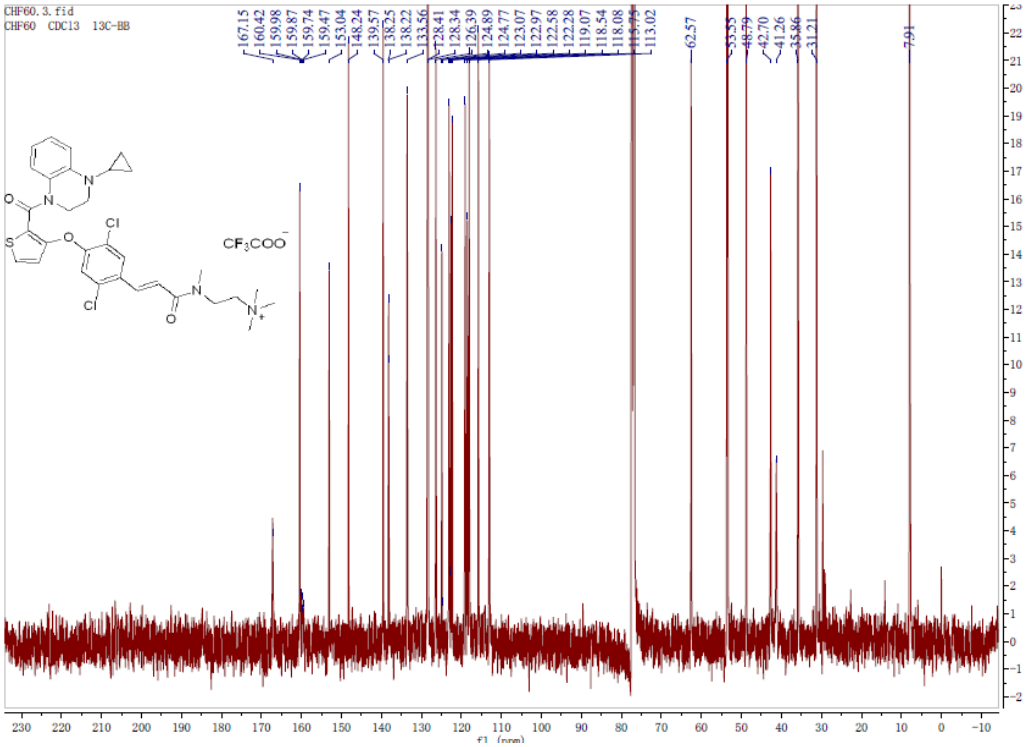

^{1}H NMR of **26a**

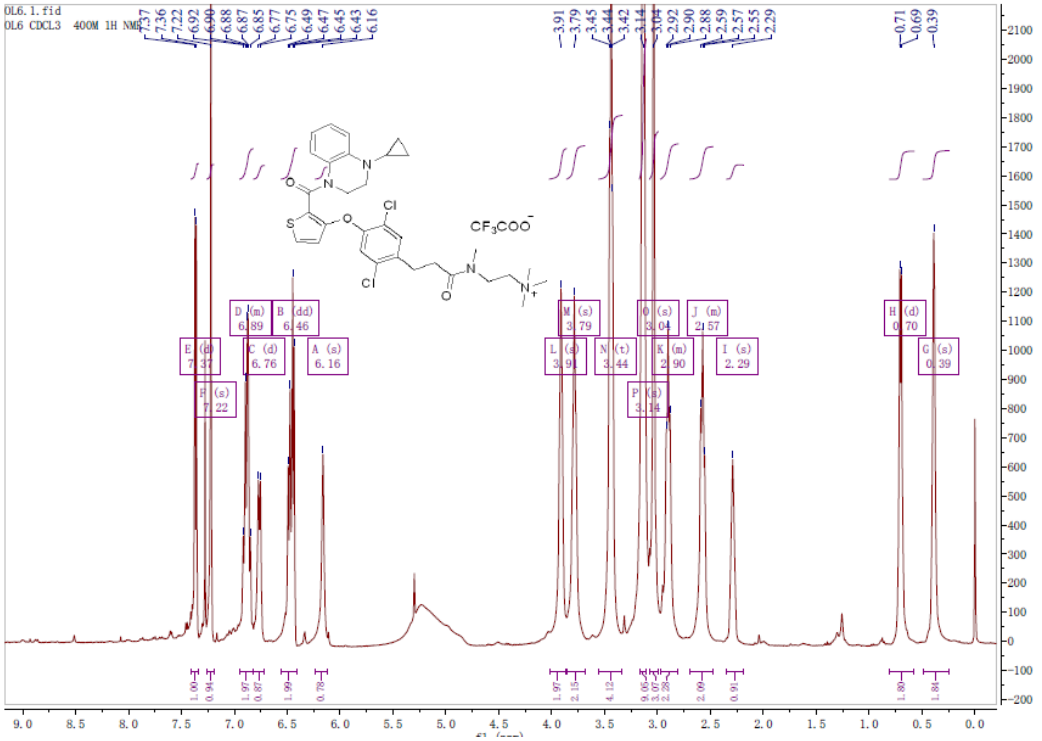

^{13}C NMR of **26a**

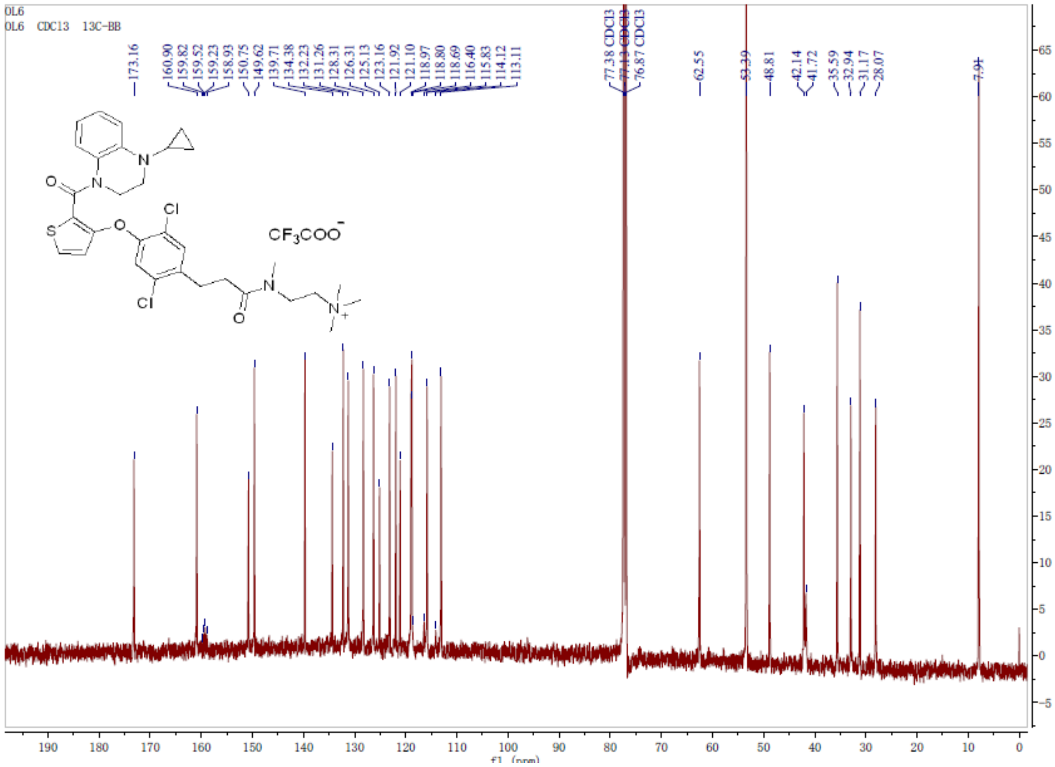

^{1}H NMR of **26b**

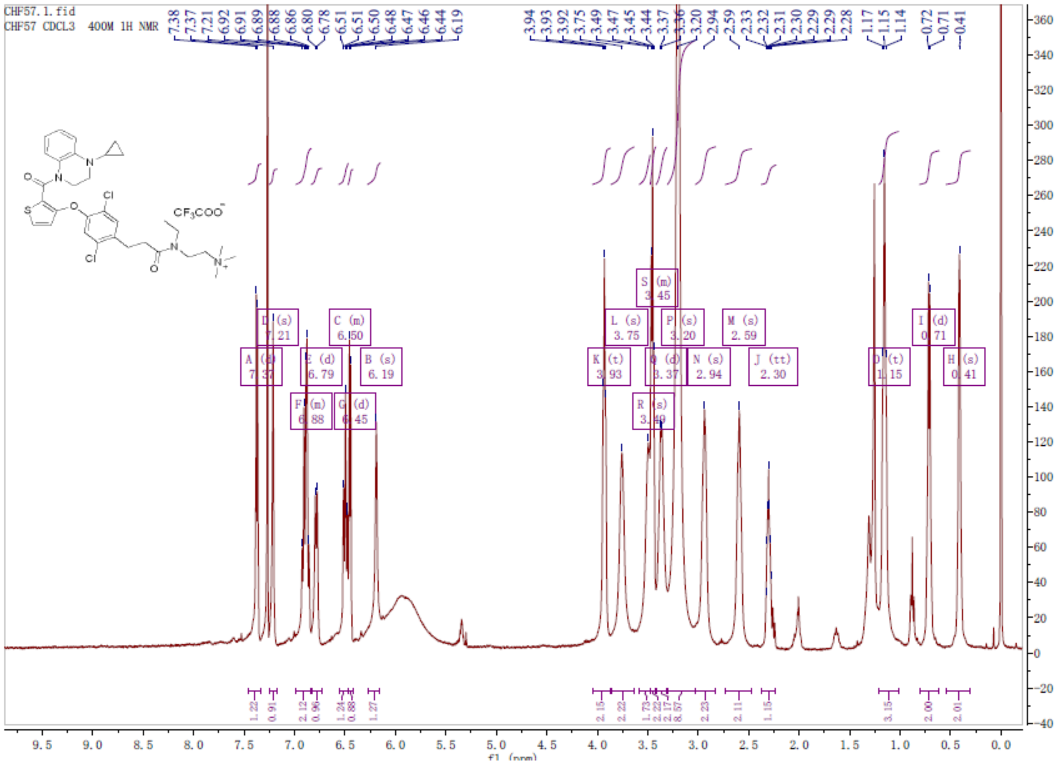

^{13}C NMR of **26b**

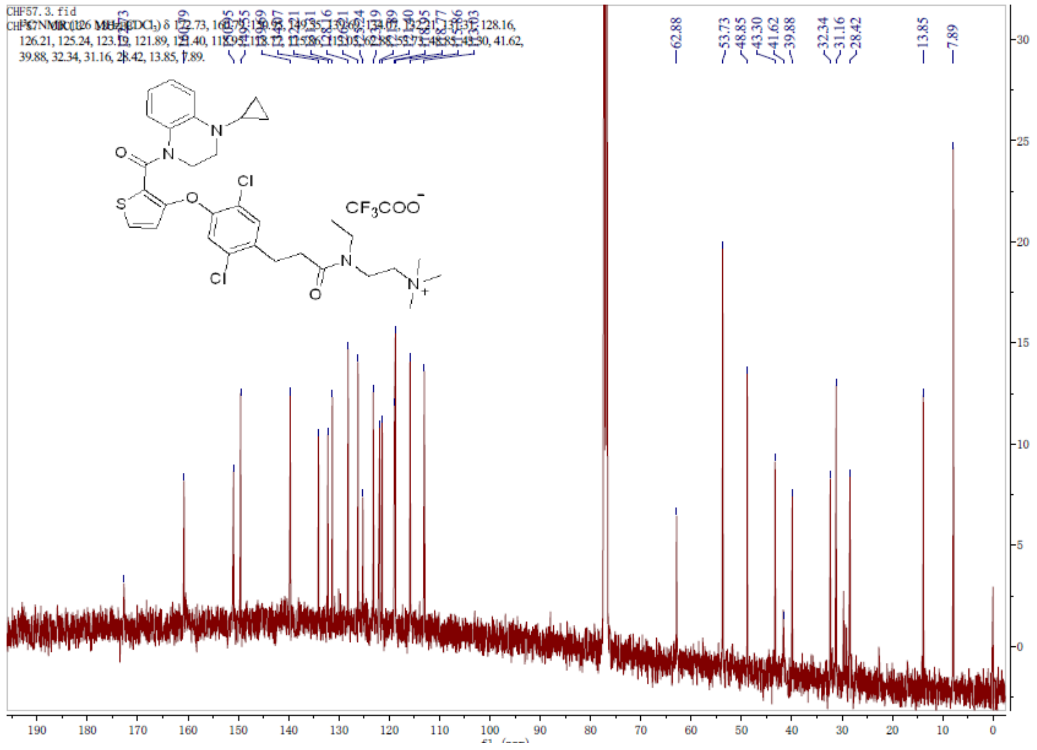

^{1}H NMR of **26c**

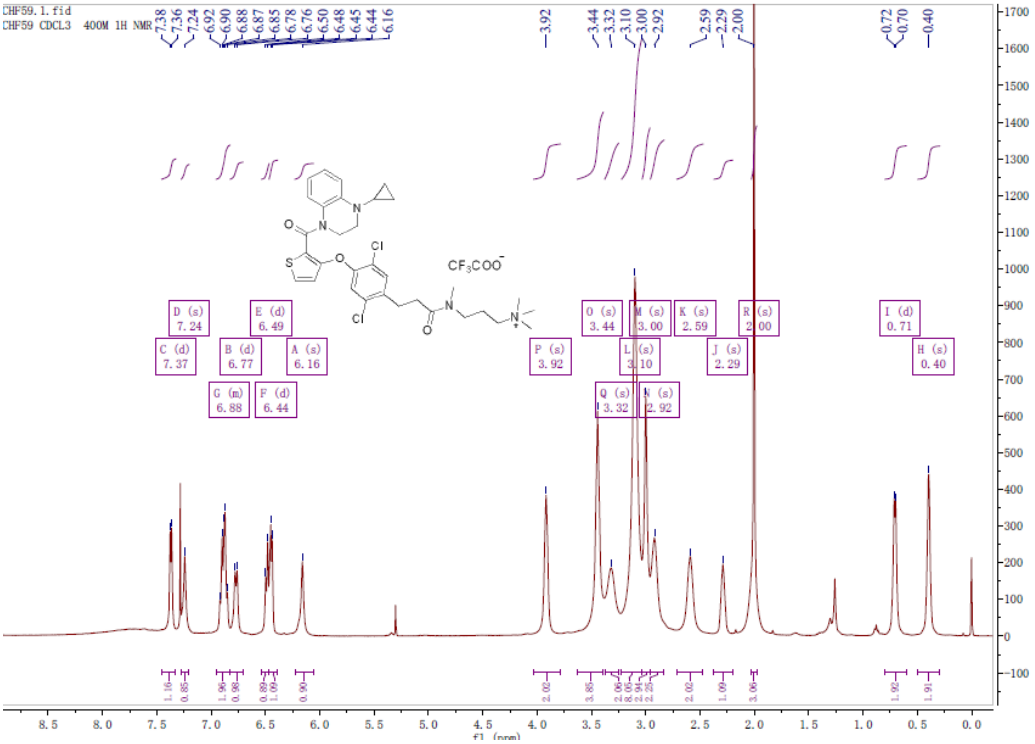

^{13}C NMR of **26c**

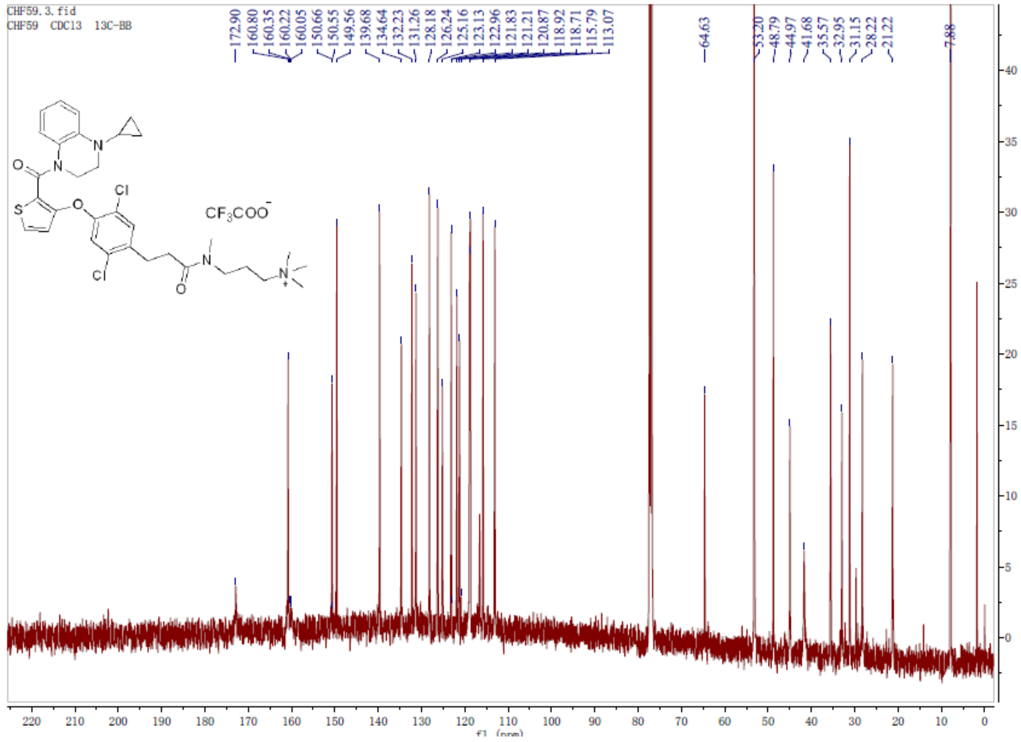

^{1}H NMR of **26d**

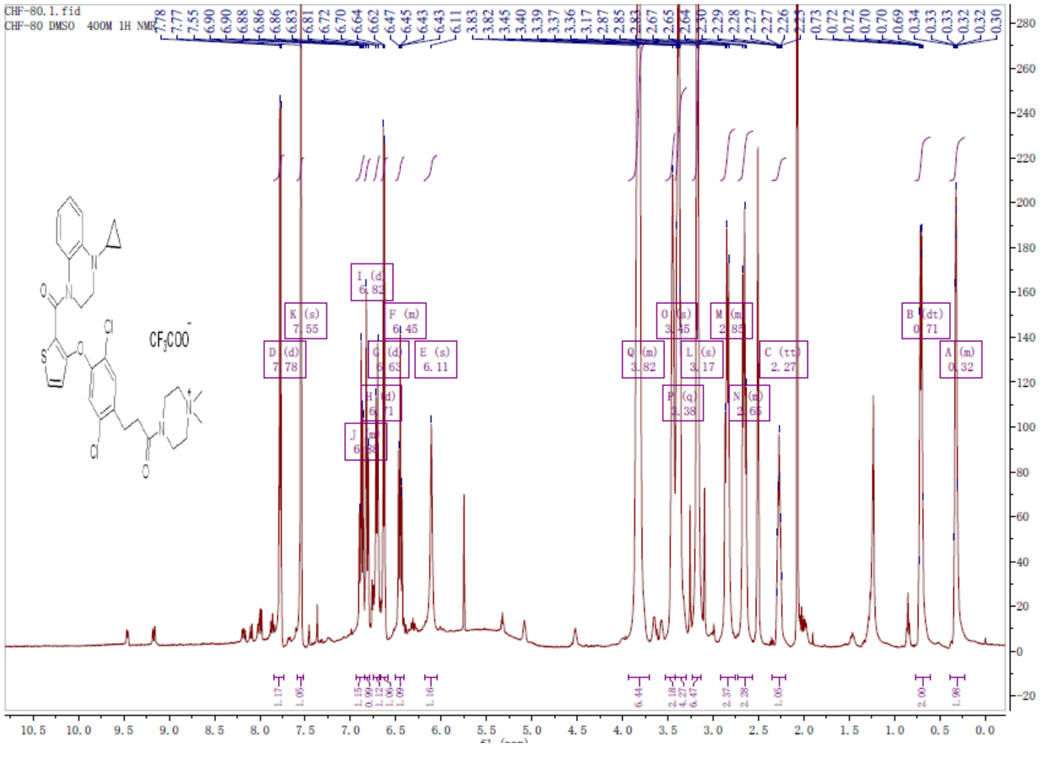

^{13}C NMR of **26d**

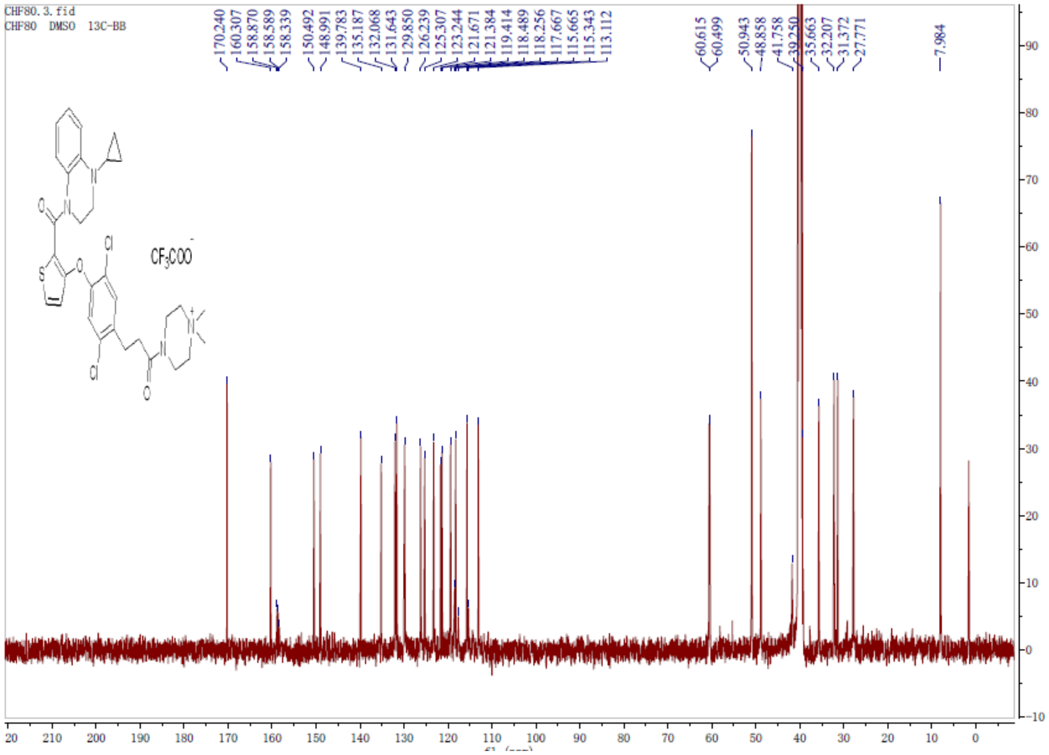

^{1}H NMR of **26e**

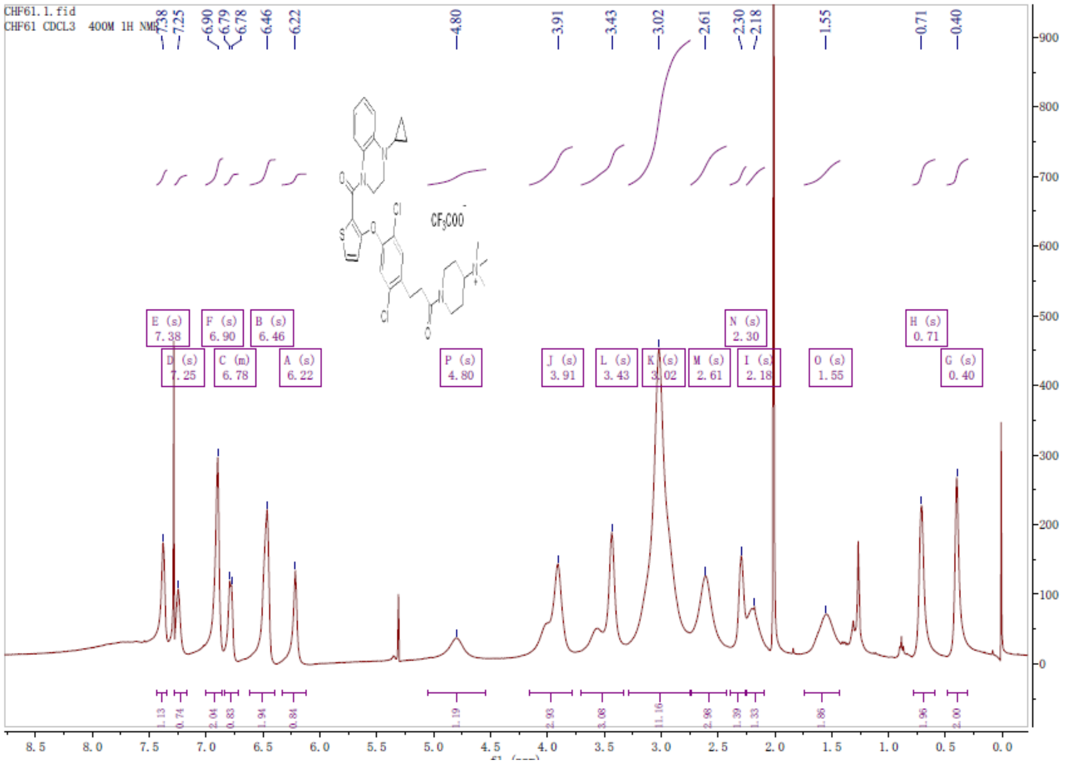

^{13}C NMR of **26e**

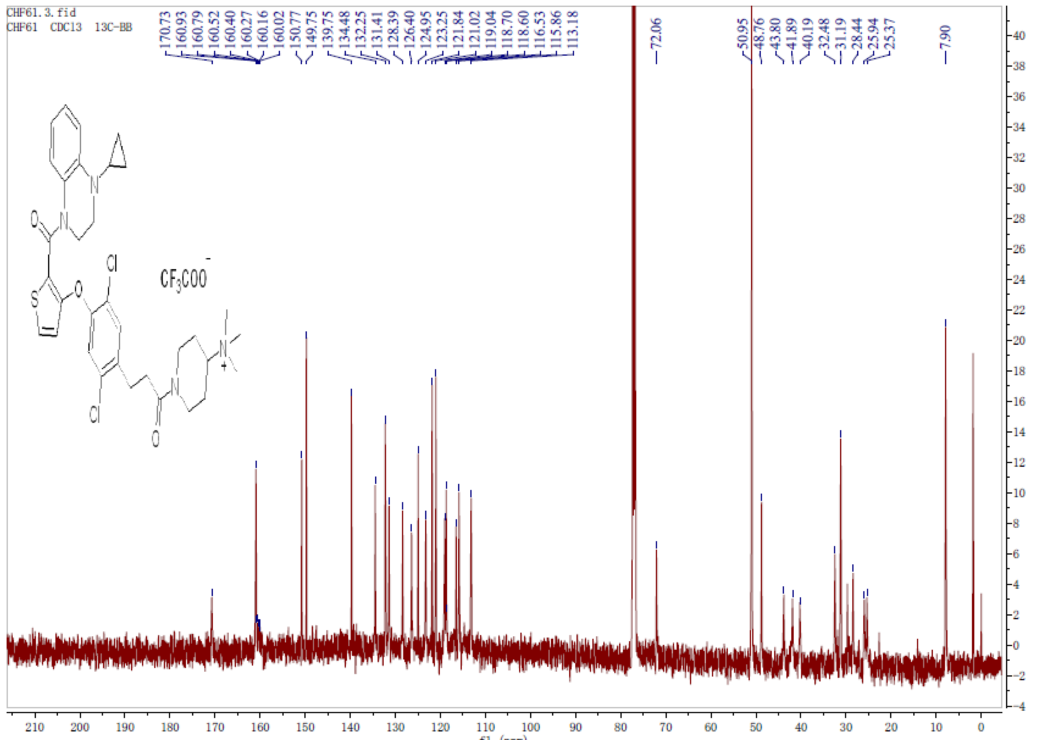

^{1}H NMR of **26f**

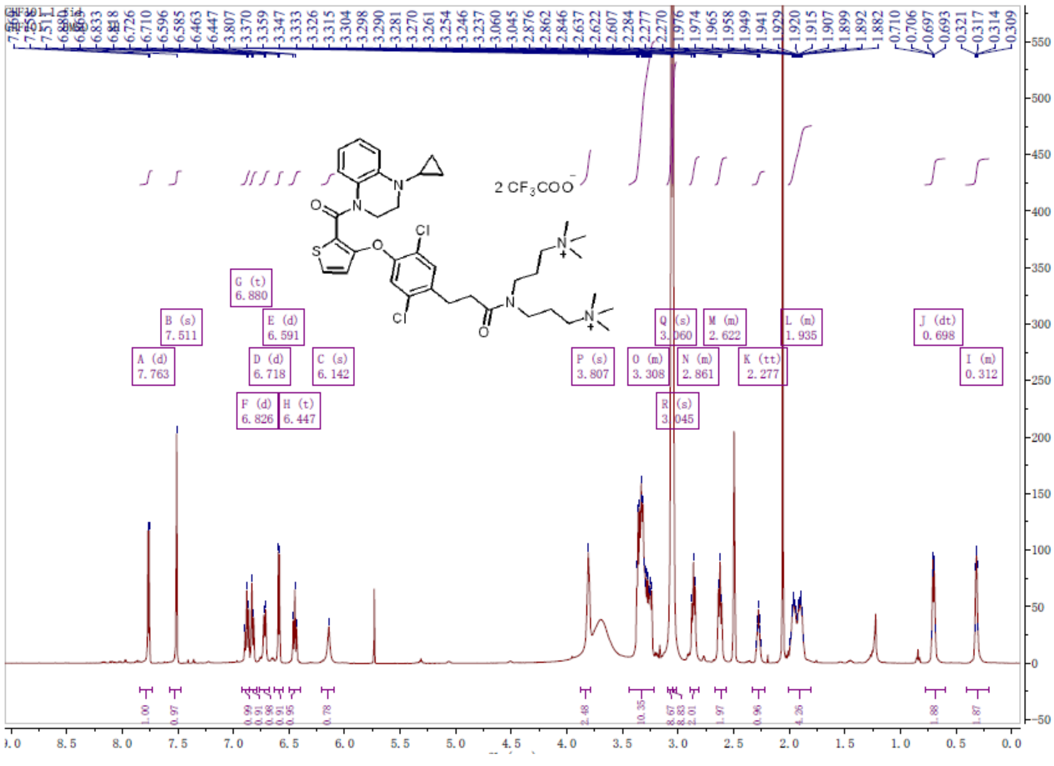

^{13}C NMR of **26f**

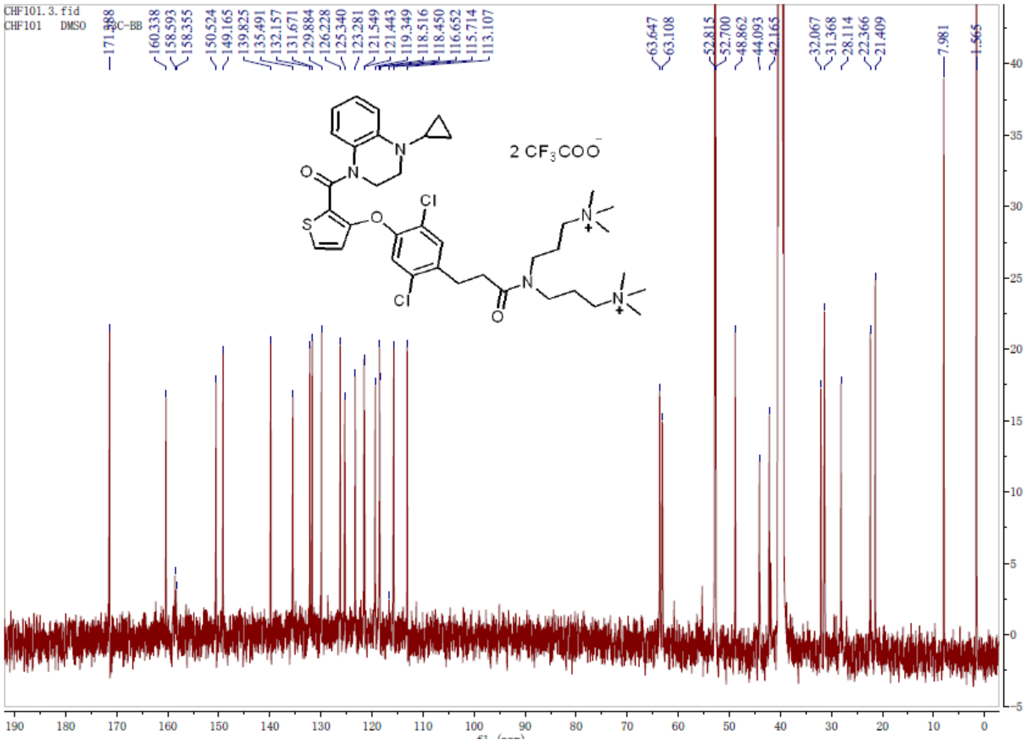

^{1}H NMR of **27a**

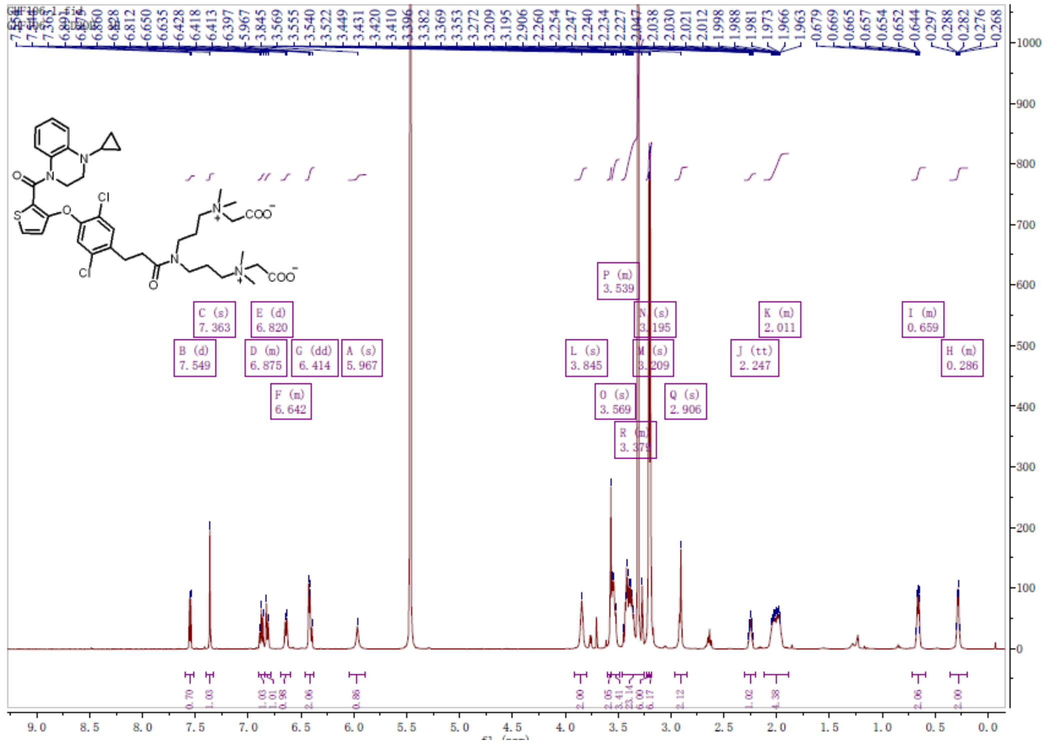

^{13}C NMR of **27a**

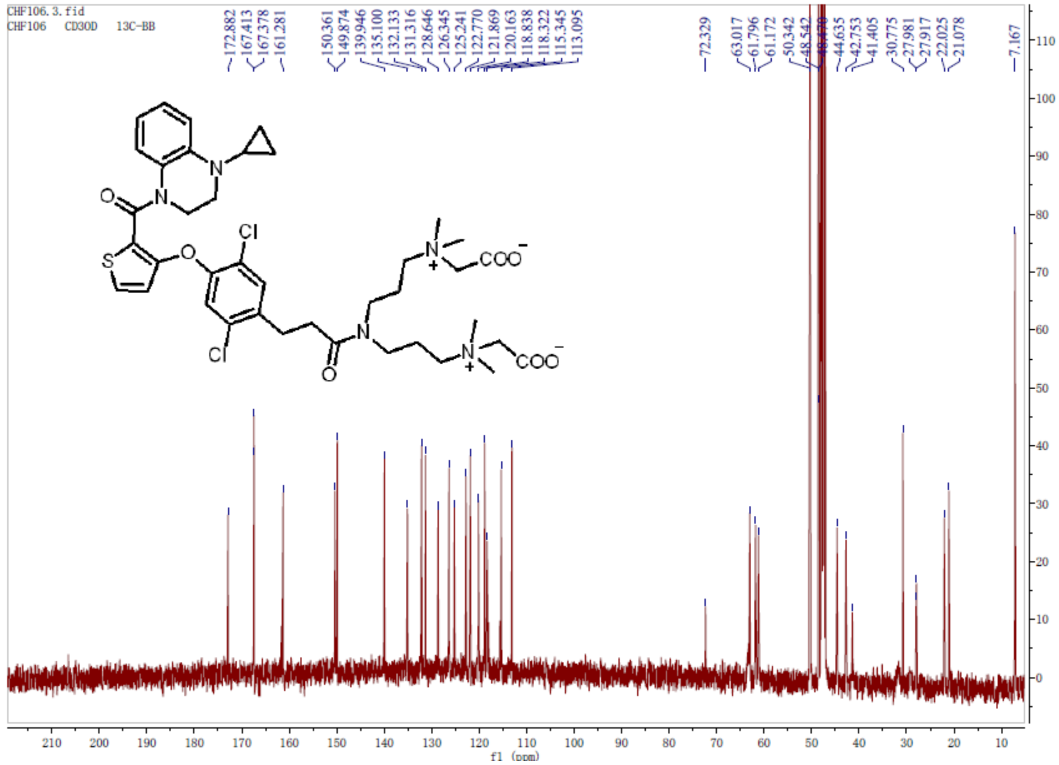

^{1}H NMR of **27b**

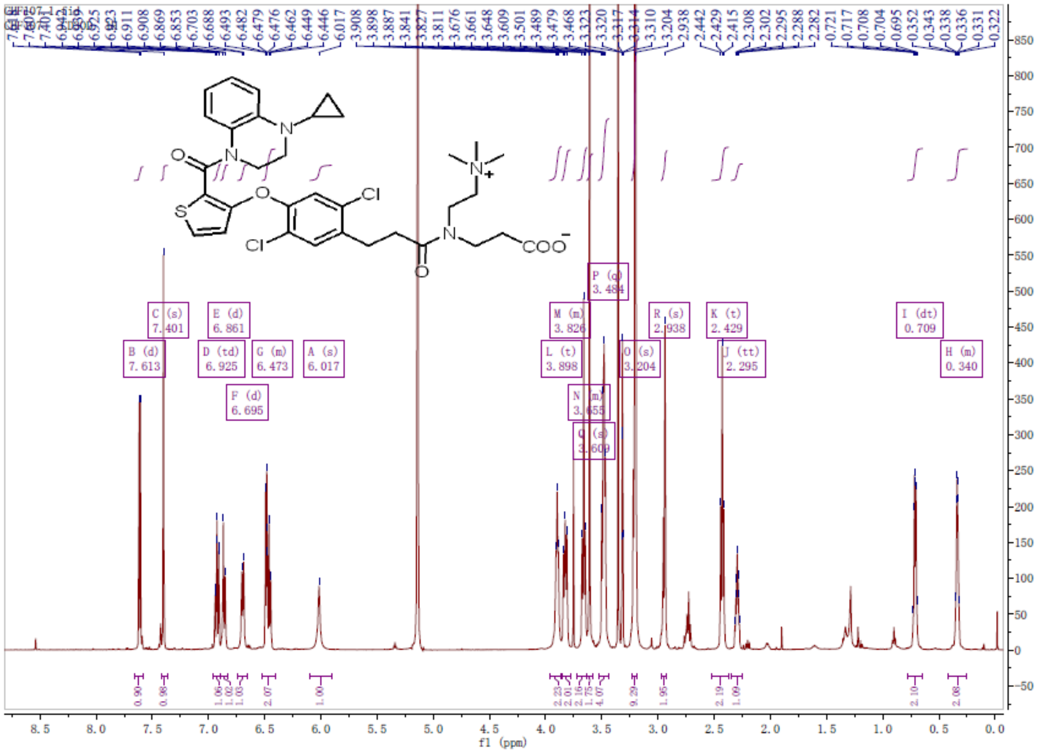

^{13}C NMR of **27b**

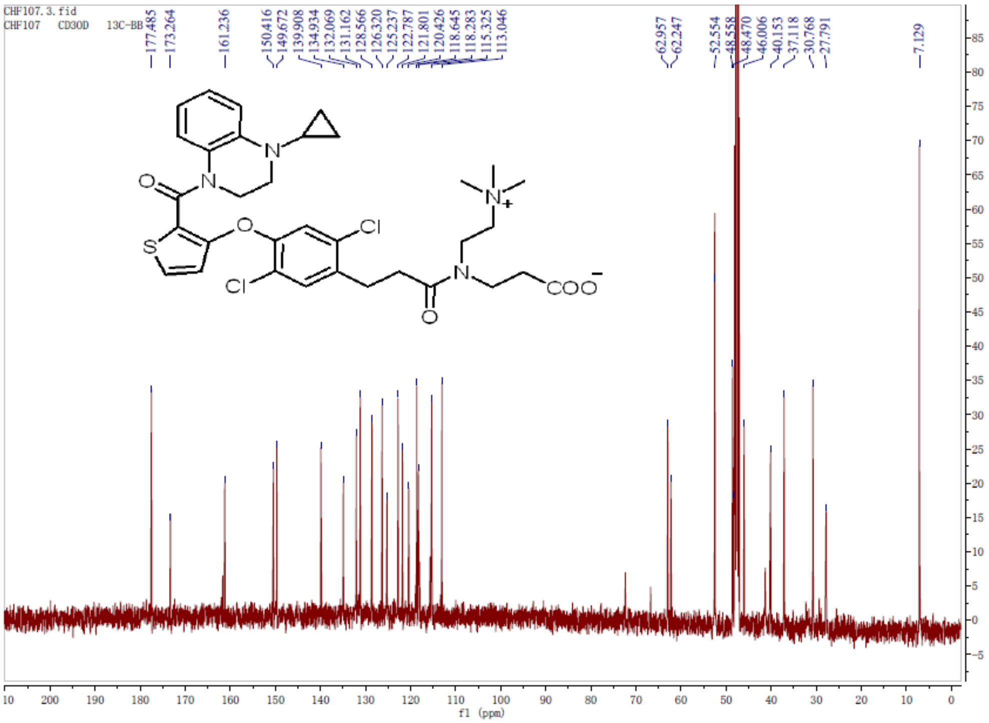

^{1}H NMR of **30**

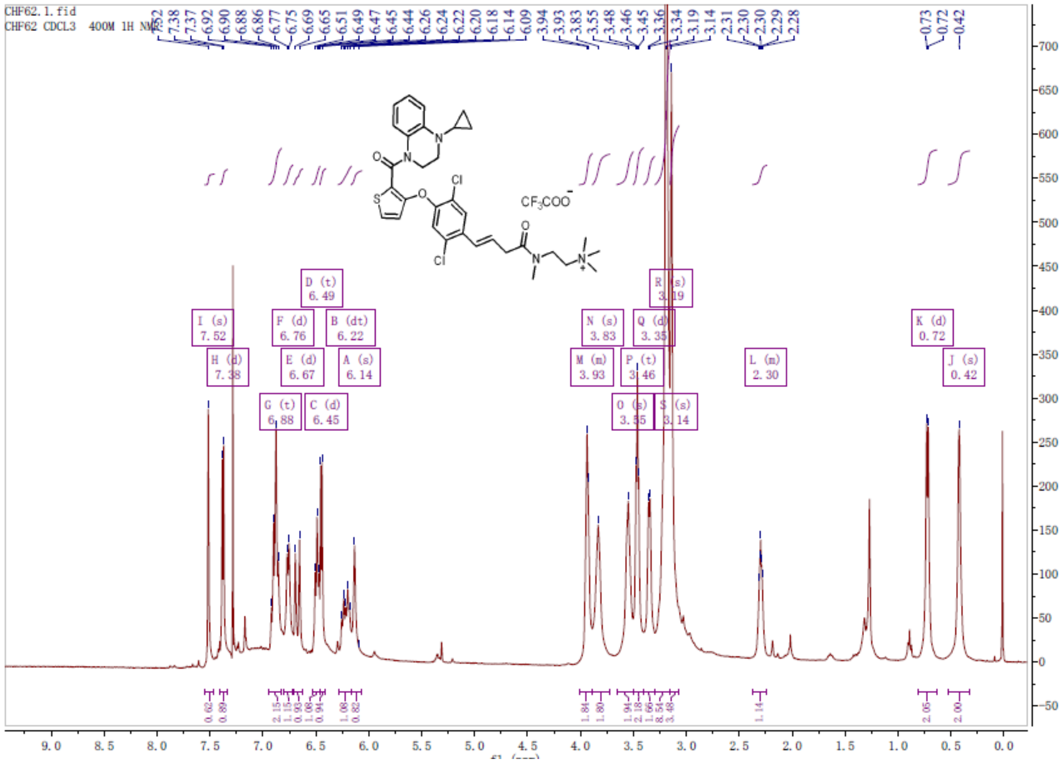

^{13}C NMR of **30**

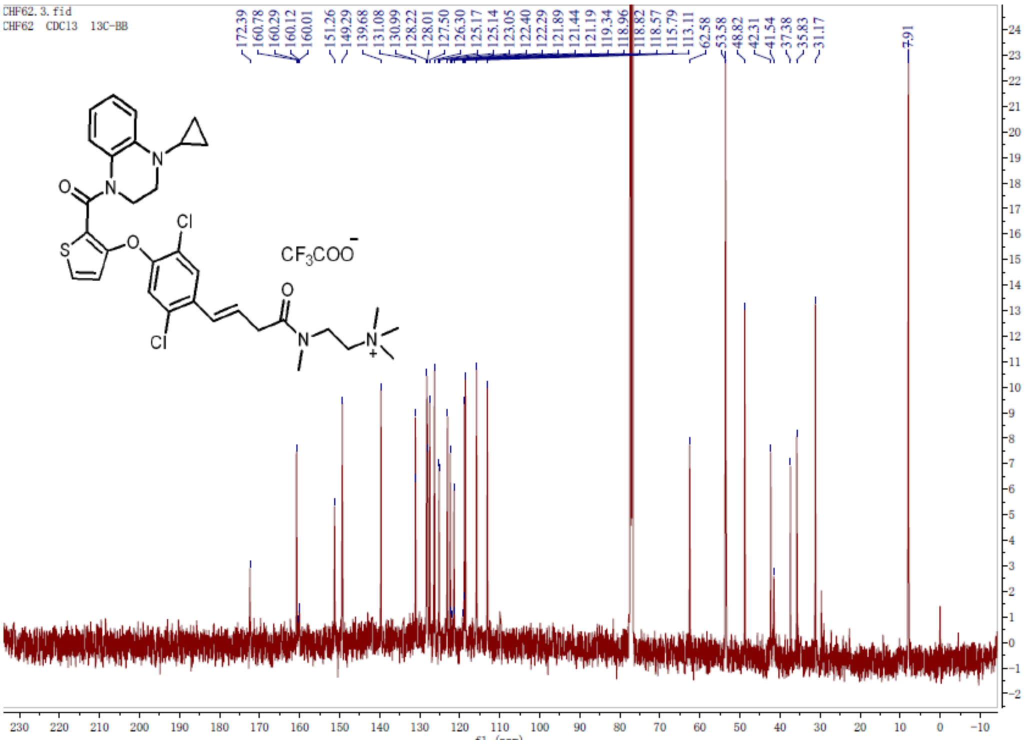

^{1}H NMR of **33**

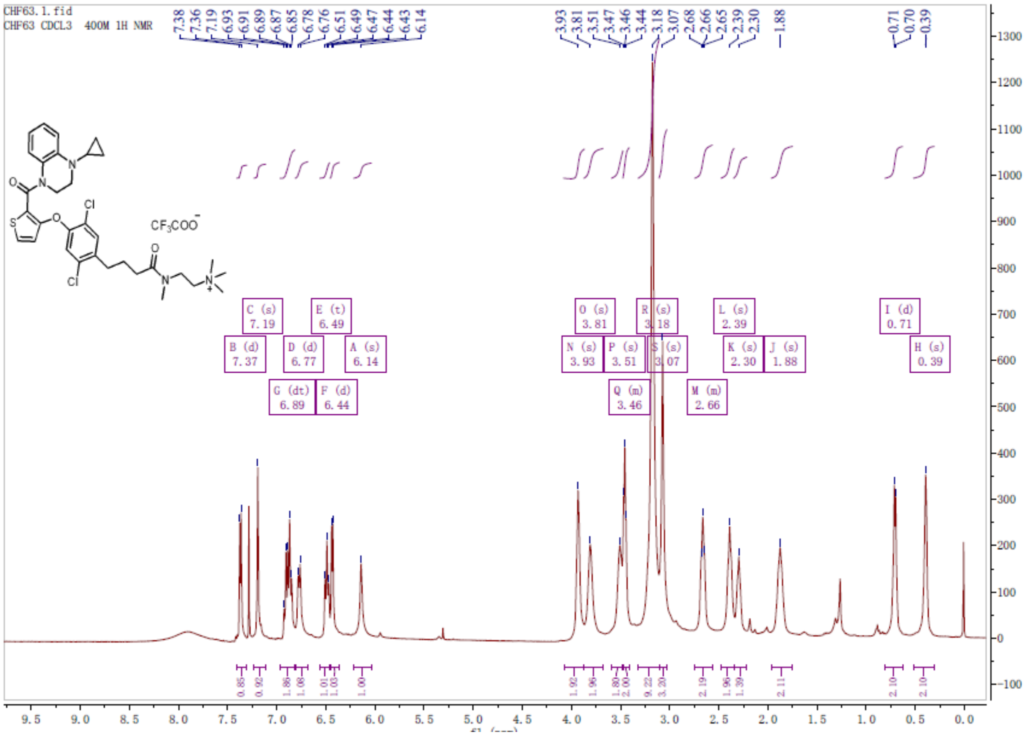

^{13}C NMR of **33**

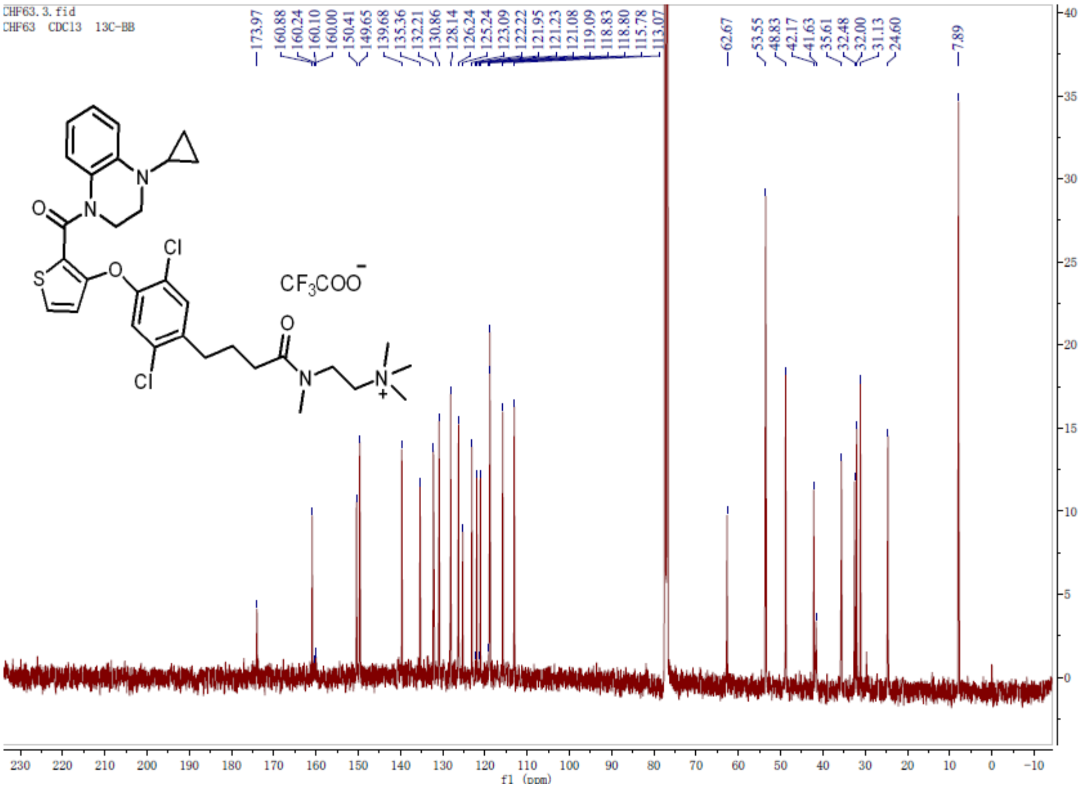

Supplement: Supplementary Information [file srep28676-s1.pdf]
